# Supplementary material for: Tuning the Nucleophilicity and Electrophilicity of Group 10 Elements through Substituent Effects: A DFT Study
Source: Int J Mol Sci. 2023 Oct 26;24(21):15597. doi: 10.3390/ijms242115597 (PMC10648789; doi:10.3390/ijms242115597)
Supplement: Supplementary file 1 [file ijms-24-15597-s001.zip › ijms-2663337-supplementary.pdf]

Supplementary Material for manuscript:

Tuning the nucleophilicity and electrophilicity of Group 10 elements through substituent effects: A DFT study

Sergi Burguera[1], Antonio Bauzá[1] and Antonio Frontera[1,*]

[1]Departament de Química, Universitat de les Illes Balears, Crta de Valldemossa Km, 7.5, 07122 Palma de mallorca (Baleares), SPAIN.
**Correspondence: toni.frontera@uib.es*

Table of contents:

**Supplementary Figure 1 (Figure S1)**	**Page 2**
**Supplementary Figure 2 (Figure S2)**	**Page 3**
**Supplementary Figure 3 (Figure S3)**	**Page 4**
**Supplementary Figure 4 (Figure S4)**	**Page 5**
**Supplementary Figure 5 (Figure S5)**	**Page 6**
**Supplementary Figure 6 (Figure S6)**	**Page 7**
**Cartesian coordinates for the MEP surface calculations**	**Pages 8-39**
**Cartesian coordinates of the optimized pyridine dimers**	**Pages 40-84**
**Cartesian coordinates of the optimized pentafluoroiodobenzene dimers**	**Pages 85-130**

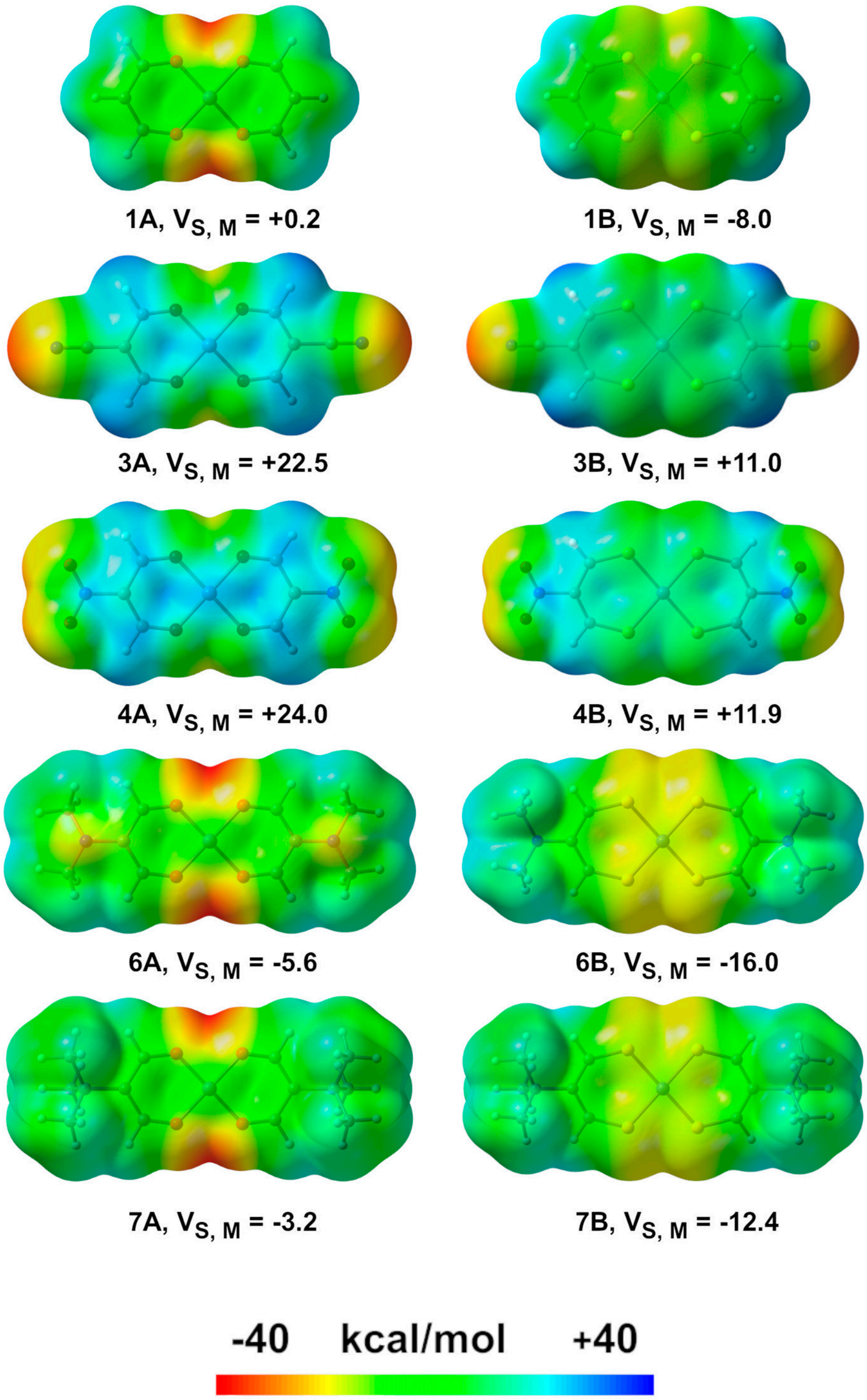

Figure S1. Molecular electrostatic potential surfaces of all the remaining Ni^{II} monomers. Isosurface = 0.001 a.u

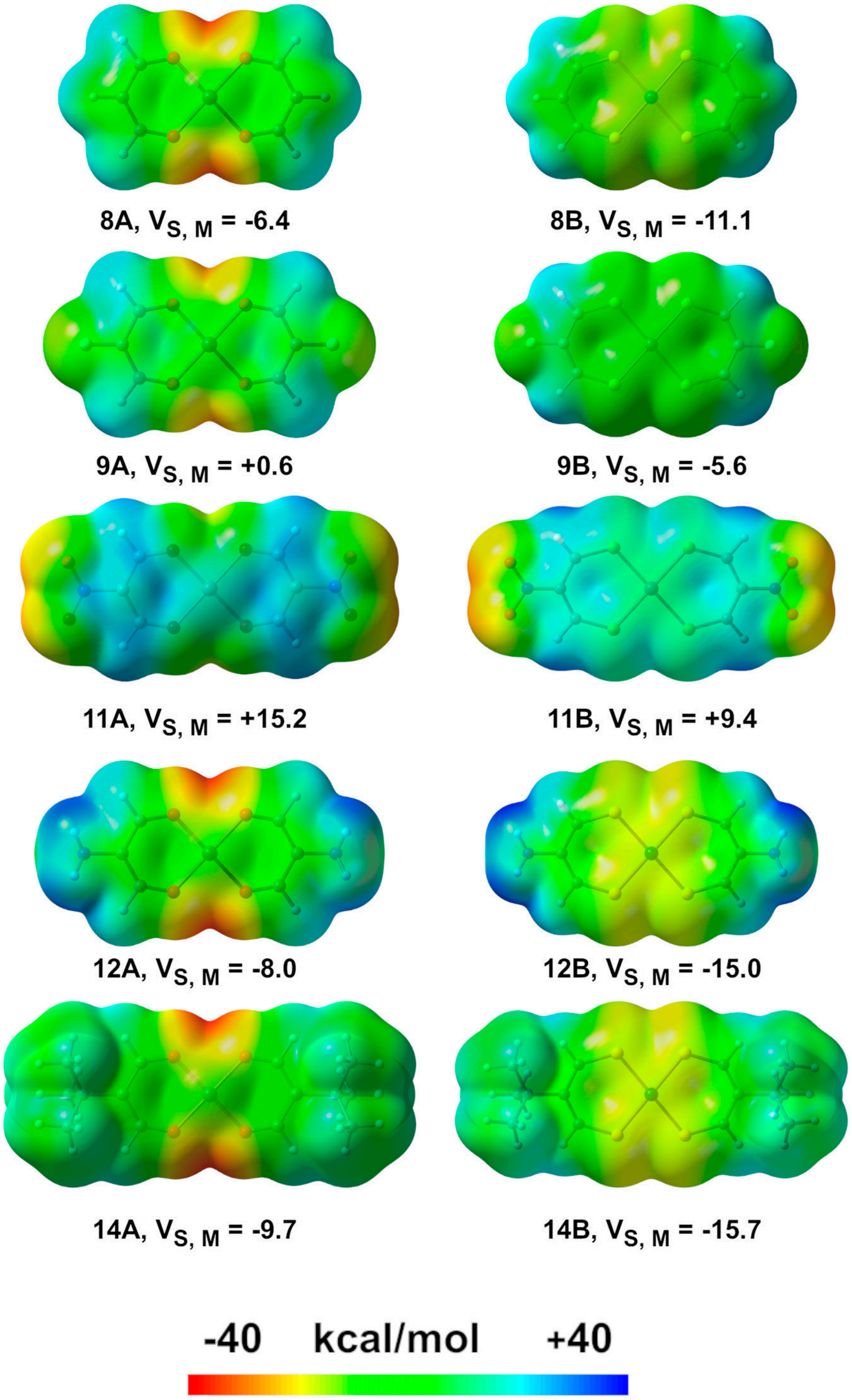

Figure S2. Molecular electrostatic potential surfaces of all the remaining Pd^{II} monomers. Isosurface = 0.001 a.u

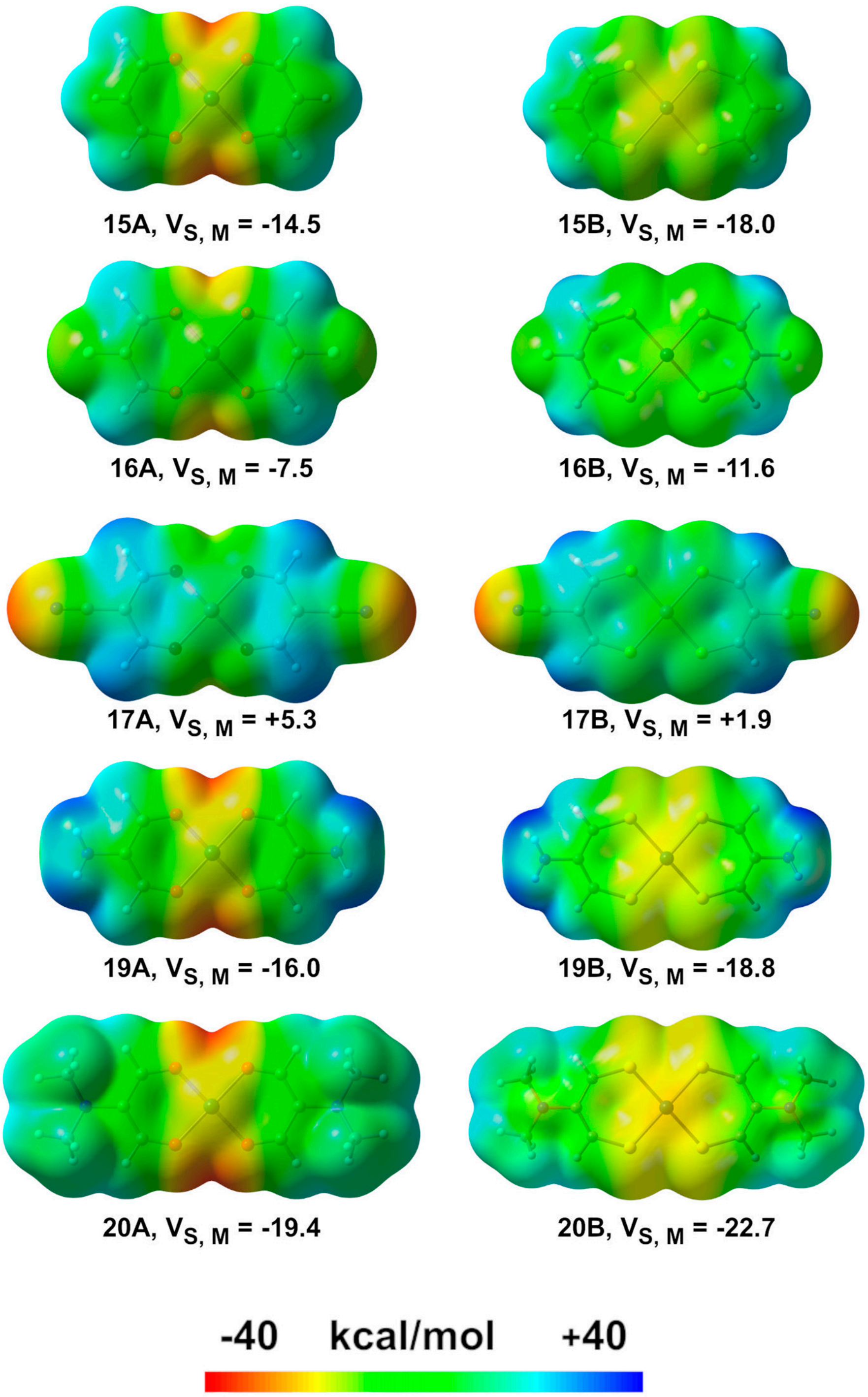

Figure S3. Molecular electrostatic potential surfaces of all the remaining Pt^{II} monomers. Isosurface = 0.001 a.u

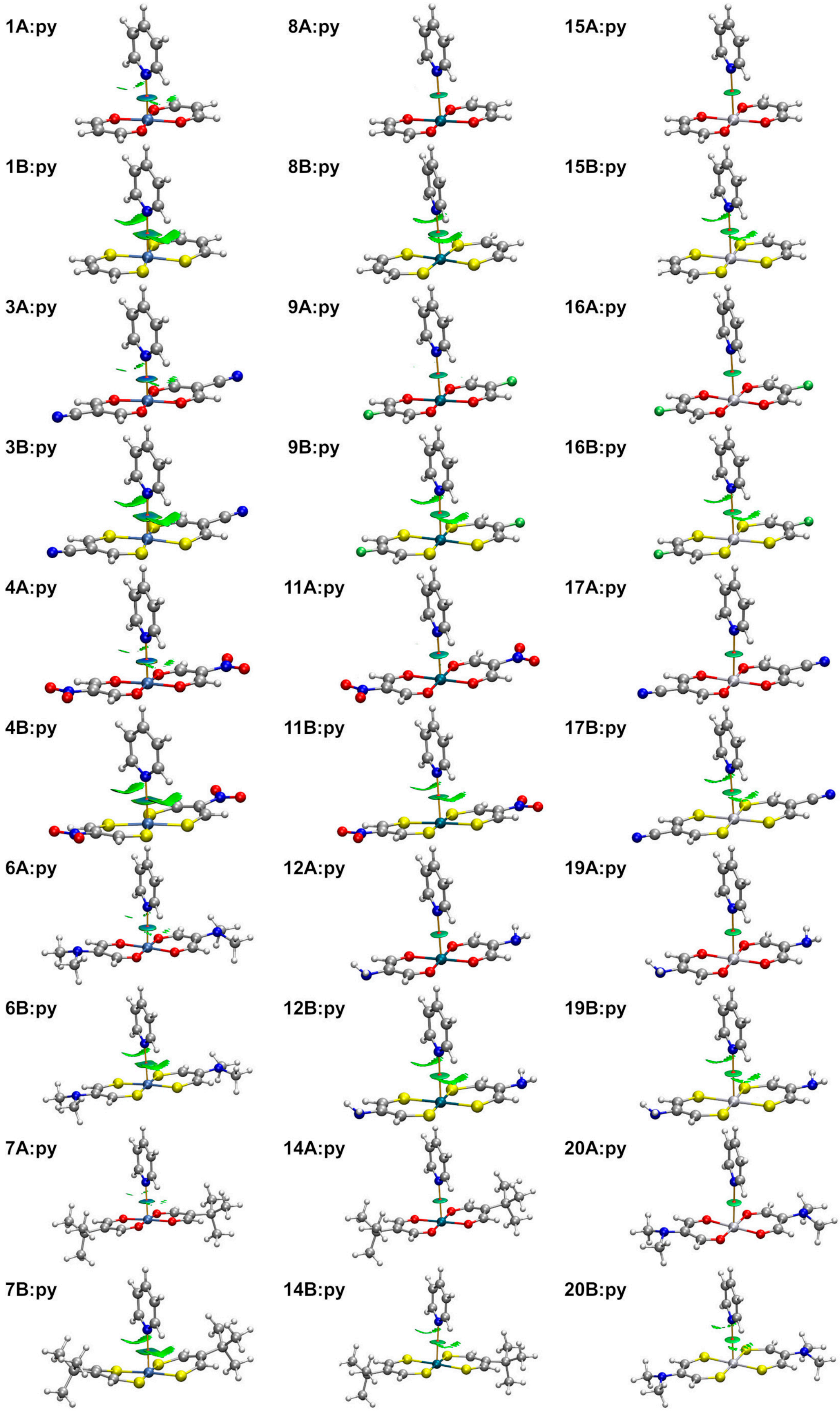

Figure S4. AIM/NCIplot of all the remaining pyridine dimers. The settings are the same as in Figure 2.

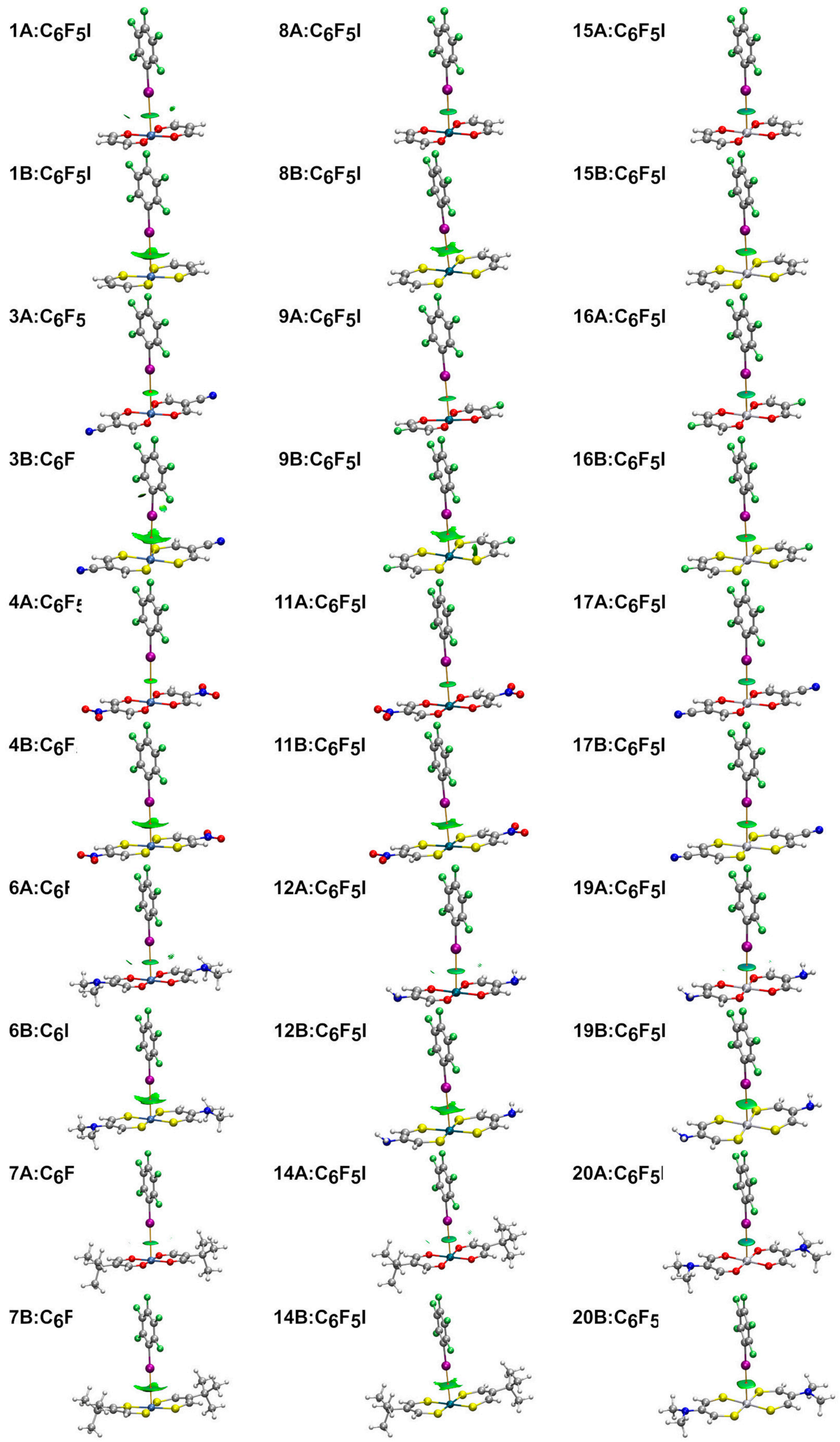

Figure S5. AIM/NCIplot of all the remaining pentafluoroiodobenzene dimers. The settings are the same as in Figure 3.

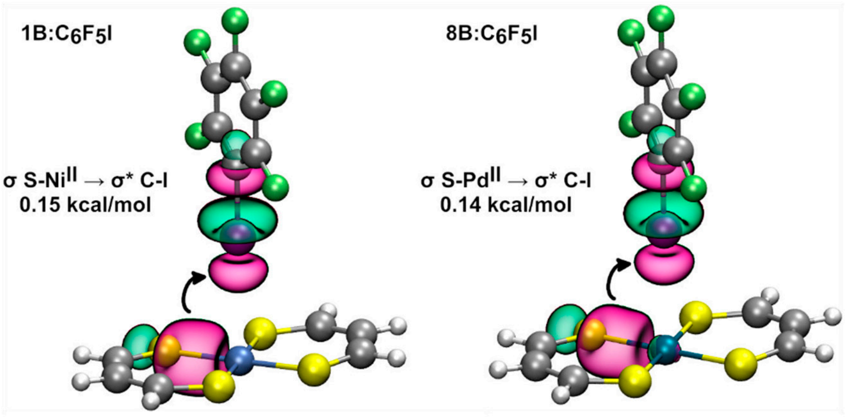

Figure S6. Alternative donor for the NBO analyses for the B set, pentafluoroiodobenzene dimers. Only one of the four σ S-M^{II} donor orbitals are shown. Settings are the same as in Figure 5.

Cartesian coordinates for the MEP surface calculations

1A

C	2.5023126	-1.2071188	0.0000000
H	3.0850711	-2.1355893	0.0000000
C	3.1898628	0.0000000	0.0000000
H	4.2704195	0.0000000	0.0000000
C	2.5023126	1.2071188	0.0000000
H	3.0850711	2.1355893	-0.0000000
O	1.2533403	-1.3659444	-0.0000000
O	1.2533403	1.3659444	0.0000000
C	-2.5023126	1.2071188	-0.0000000
H	-3.0850711	2.1355893	0.0000000
C	-3.1898628	0.0000000	0.0000000
H	-4.2704195	-0.0000000	0.0000000
C	-2.5023126	-1.2071188	0.0000000
H	-3.0850711	-2.1355893	0.0000000
O	-1.2533403	1.3659444	-0.0000000
O	-1.2533403	-1.3659444	0.0000000
Ni	0.0000000	0.0000000	0.0000000

1B

C	3.0436892	0.0000000	-1.2301293
H	3.6899236	0.0000000	-2.1055644
C	3.6742493	0.0000000	-0.0000159
H	4.7587928	0.0000000	-0.0000093
C	3.0437239	0.0000000	1.2301223
H	3.6900469	0.0000000	2.1055032
C	-3.0437239	0.0000000	1.2301223
H	-3.6900469	-0.0000000	2.1055032
C	-3.6742493	0.0000000	-0.0000159
H	-4.7587928	-0.0000000	-0.0000093
C	-3.0436892	0.0000000	-1.2301293
H	-3.6899236	0.0000000	-2.1055644
S	1.4330678	0.0000000	1.6407852

S	1.4330067	0.0000000	-1.6407189
S	-1.4330678	0.0000000	1.6407852
S	-1.4330067	0.0000000	-1.6407189
Ni	0.0000000	0.0000000	0.0000541

2A

C	2.5022981	-1.2163789	-0.0000000
H	3.1118618	-2.1262549	-0.0000000
C	3.1640484	0.0000000	0.0000000
C	2.5022981	1.2163789	0.0000000
H	3.1118618	2.1262549	0.0000000
O	1.2512385	-1.3683709	-0.0000000
O	1.2512385	1.3683709	0.0000000
C	-2.5022981	1.2163789	0.0000000
H	-3.1118618	2.1262549	0.0000000
C	-3.1640484	0.0000000	-0.0000000
C	-2.5022981	-1.2163789	0.0000000
H	-3.1118618	-2.1262549	0.0000000
O	-1.2512385	1.3683709	-0.0000000
O	-1.2512385	-1.3683709	-0.0000000
Ni	0.0000000	0.0000000	0.0000000
F	4.5130482	0.0000000	0.0000000
F	-4.5130482	0.0000000	0.0000000

2B

C	3.0379070	-1.2365004	-0.0000005
H	3.7152969	-2.0874923	-0.0000196
C	3.6414828	0.0000000	-0.0000209
C	3.0379070	1.2365004	-0.0000005
H	3.7152969	2.0874923	-0.0000196
C	-3.0379070	1.2365004	-0.0000005
H	-3.7152969	2.0874923	-0.0000196
C	-3.6414828	0.0000000	-0.0000209
C	-3.0379070	-1.2365004	-0.0000005
H	-3.7152969	-2.0874923	-0.0000196

F	4.9926236	0.0000000	-0.0000636
F	-4.9926236	0.0000000	-0.0000636
S	1.4278055	1.6452604	0.0000473
S	1.4278055	-1.6452604	0.0000473
S	-1.4278055	1.6452604	0.0000473
S	-1.4278055	-1.6452604	0.0000473
Ni	0.0000000	0.0000000	0.0000600

3A

C	2.5002775	-1.2187976	-0.0000004
H	3.0872458	-2.1430568	-0.0000235
C	3.1952160	-0.0000000	-0.0000083
C	2.5002775	1.2187976	-0.0000004
H	3.0872458	2.1430568	-0.0000235
O	1.2570244	-1.3595715	0.0000306
O	1.2570244	1.3595715	0.0000306
C	-2.5002775	1.2187976	-0.0000004
H	-3.0872458	2.1430568	-0.0000235
C	-3.1952160	0.0000000	-0.0000083
C	-2.5002775	-1.2187976	-0.0000004
H	-3.0872458	-2.1430568	-0.0000235
O	-1.2570244	1.3595715	0.0000306
O	-1.2570244	-1.3595715	0.0000306
Ni	0.0000000	0.0000000	0.0000424
C	-4.6147186	0.0000000	-0.0000161
C	4.6147186	0.0000000	-0.0000161
N	5.7673013	0.0000000	-0.0000101
N	-5.7673013	0.0000000	-0.0000101

3B

C	-1.2385552	0.0000000	3.0357390
H	-2.1101686	-0.0000000	3.6866244
C	0.0000000	0.0000000	3.6746700
C	1.2385552	0.0000000	3.0357390
H	2.1101686	-0.0000000	3.6866244

C	1.2385552	-0.0000000	-3.0357390
H	2.1101686	0.0000000	-3.6866244
C	0.0000000	0.0000000	-3.6746700
C	-1.2385552	0.0000000	-3.0357390
H	-2.1101686	0.0000000	-3.6866244
Ni	0.0000000	0.0000000	0.0000000
C	0.0000000	0.0000000	-5.1032532
C	0.0000000	0.0000000	5.1032532
N	0.0000000	0.0000000	6.2553717
N	0.0000000	-0.0000000	-6.2553717
S	1.6381804	-0.0000000	-1.4316940
S	-1.6381804	0.0000000	-1.4316940
S	-1.6381804	0.0000000	1.4316940
S	1.6381804	-0.0000000	1.4316940

4A

C	2.4998186	-1.2246449	0.0000112
H	3.0998787	-2.1373093	0.0000013
C	3.1736503	0.0000000	-0.0000167
C	2.4998186	1.2246449	0.0000112
H	3.0998787	2.1373093	0.0000013
O	1.2558258	-1.3611271	0.0000700
O	1.2558258	1.3611271	0.0000700
C	-2.4998186	1.2246449	0.0000112
H	-3.0998787	2.1373093	0.0000013
C	-3.1736503	0.0000000	-0.0000167
C	-2.4998186	-1.2246449	0.0000112
H	-3.0998787	-2.1373093	0.0000013
O	-1.2558258	1.3611271	0.0000700
O	-1.2558258	-1.3611271	0.0000700
Ni	0.0000000	0.0000000	0.0000743
N	-4.6161385	0.0000000	-0.0000537
N	4.6161385	0.0000000	-0.0000537
O	-5.1801110	-1.0760111	-0.0000659
O	-5.1801110	1.0760111	-0.0000659

O	5.1801110	-1.0760111	-0.0000659
O	5.1801110	1.0760111	-0.0000659

4B

C	3.0493339	-1.2422851	0.0000184
H	3.7258729	-2.1055124	-0.0000862
C	3.6986224	0.0000000	-0.0000320
C	3.0493339	1.2422851	0.0000184
H	3.7258729	2.1055124	-0.0000862
C	-3.0493339	1.2422851	0.0000184
H	-3.7258729	2.1055124	-0.0000862
C	-3.6986224	0.0000000	-0.0000320
C	-3.0493339	-1.2422851	0.0000184
H	-3.7258729	-2.1055124	-0.0000862
N	-5.0451802	0.0000000	-0.0001025
N	5.0451802	0.0000000	-0.0001025
O	-5.7574457	-1.0644388	-0.0001302
O	-5.7574457	1.0644388	-0.0001302
O	5.7574457	-1.0644388	-0.0001302
O	5.7574457	1.0644388	-0.0001302
S	1.4538784	1.5956497	0.0002210
S	1.4538784	-1.5956497	0.0002210
S	-1.4538784	-1.5956497	0.0002210
S	-1.4538784	1.5956497	0.0002210
Ni	0.0000000	0.0000000	0.0001769

5A

C	2.5142901	-1.1977013	0.0000148
H	3.0929940	-2.1310481	-0.0000537
C	3.2240793	0.0000000	0.0000157
C	2.5142901	1.1977013	0.0000148
H	3.0929940	2.1310481	-0.0000537
O	1.2587719	-1.3508047	0.0000765
O	1.2587719	1.3508047	0.0000765
C	-2.5142901	1.1977013	0.0000148

H	-3.0929940	2.1310481	-0.0000537
C	-3.2240793	0.0000000	0.0000157
C	-2.5142901	-1.1977013	0.0000148
H	-3.0929940	-2.1310481	-0.0000537
O	-1.2587719	1.3508047	0.0000765
O	-1.2587719	-1.3508047	0.0000765
Ni	0.0000000	0.0000000	0.0001156
N	-4.6211147	0.0000000	0.0002221
N	4.6211147	0.0000000	0.0002221
H	-5.1343131	0.8567530	-0.0001854
H	-5.1343131	-0.8567530	-0.0001854
H	5.1343131	0.8567530	-0.0001854
H	5.1343131	-0.8567530	-0.0001854

5B

C	2.9539406	-1.2670364	-0.0000006
H	3.6155834	-2.1284391	-0.0001534
C	3.5709853	0.0000000	-0.0000605
C	2.9539406	1.2670364	-0.0000006
H	3.6155834	2.1284391	-0.0001534
C	-2.9539406	1.2670364	-0.0000006
H	-3.6155834	2.1284391	-0.0001534
C	-3.5709853	0.0000000	-0.0000605
C	-2.9539406	-1.2670364	-0.0000006
H	-3.6155834	-2.1284391	-0.0001534
N	-4.9254654	-0.0000000	-0.0001855
N	4.9254654	0.0000000	-0.0001855
H	-5.4392392	0.8614810	-0.0001261
H	-5.4392392	-0.8614810	-0.0001261
H	5.4392392	0.8614810	-0.0001261
H	5.4392392	-0.8614810	-0.0001261
S	1.3257495	1.6688031	0.0003020
S	1.3257495	-1.6688031	0.0003020
S	-1.3257495	1.6688031	0.0003020
S	-1.3257495	-1.6688031	0.0003020

Ni	0.0000000	0.0000000	0.0004042

6A

C	2.5654841	-1.0691808	-0.0000142
H	3.1995152	-1.9648619	0.0015116
C	3.2020361	0.1703254	-0.0005036
C	2.4372592	1.3368686	-0.0025506
H	2.9908527	2.2833681	-0.0034916
O	1.3260101	-1.2951506	-0.0007608
O	1.1817960	1.4273288	-0.0033762
C	-2.5654841	1.0691808	-0.0000142
H	-3.1995152	1.9648619	0.0015116
C	-3.2020361	-0.1703254	-0.0005036
C	-2.4372592	-1.3368686	-0.0025506
H	-2.9908527	-2.2833681	-0.0034916
O	-1.3260101	1.2951506	-0.0007608
O	-1.1817960	-1.4273288	-0.0033762
Ni	0.0000000	0.0000000	-0.0021218
N	-4.6199539	-0.2914530	0.0014645
N	4.6199539	0.2914530	0.0014645
C	-5.2637913	0.1860319	1.2064988
H	-5.2436916	1.2847348	1.3115446
H	-6.3115112	-0.1264869	1.2115834
H	-4.7745842	-0.2468170	2.0802537
C	-5.2658476	0.1811300	-1.2043080
H	-4.7876694	-0.2660485	-2.0770171
H	-6.3171357	-0.1189908	-1.1991608
H	-5.2333188	1.2784233	-1.3206127
C	5.2637913	-0.1860319	1.2064988
H	5.2436916	-1.2847348	1.3115446
H	6.3115112	0.1264869	1.2115834
H	4.7745842	0.2468170	2.0802537
C	5.2658476	-0.1811300	-1.2043080
H	4.7876694	0.2660485	-2.0770171
H	6.3171357	0.1189908	-1.1991608

H	5.2333188	-1.2784233	-1.3206127

6B

C	3.1043671	-1.0569851	0.0023579
H	3.7938023	-1.9004886	0.0051684
C	3.6901014	0.1998437	-0.0002922
C	2.9763159	1.3878728	-0.0044868
H	3.5926426	2.2846903	-0.0068049
C	-3.1043671	1.0569851	0.0023579
H	-3.7938023	1.9004886	0.0051684
C	-3.6901014	-0.1998437	-0.0002922
C	-2.9763159	-1.3878728	-0.0044868
H	-3.5926426	-2.2846903	-0.0068049
N	-5.1178469	-0.3155783	0.0016973
N	5.1178469	0.3155783	0.0016973
C	-5.7429524	0.1896524	1.2059034
H	-5.6873528	1.2877508	1.3052356
H	-6.7992104	-0.0903693	1.2092922
H	-5.2679237	-0.2541495	2.0820461
C	-5.7452488	0.1829103	-1.2040691
H	-5.2795165	-0.2739024	-2.0785884
H	-6.8040159	-0.0873293	-1.1984783
H	-5.6799426	1.2794231	-1.3146316
C	5.7429524	-0.1896524	1.2059034
H	5.6873528	-1.2877508	1.3052356
H	6.7992104	0.0903693	1.2092922
H	5.2679237	0.2541495	2.0820461
C	5.7452488	-0.1829103	-1.2040691
H	5.2795165	0.2739024	-2.0785884
H	6.8040159	0.0873293	-1.1984783
H	5.6799426	-1.2794231	-1.3146316
S	-1.3457981	-1.7099740	-0.0061401
S	-1.5192800	1.5558389	0.0026079
S	1.5192800	-1.5558389	0.0026079
S	1.3457981	1.7099740	-0.0061401

Ni	0.0000000	0.0000000	-0.0016345

7A

C	2.5141772	-1.1927472	-0.0248462
H	3.0709599	-2.1345054	-0.0252861
C	3.2368928	-0.0000000	0.0157893
C	2.5141772	1.1927472	-0.0248462
H	3.0709599	2.1345054	-0.0252861
O	1.2626979	-1.3454106	-0.0646298
O	1.2626979	1.3454106	-0.0646298
C	-2.5141772	1.1927472	-0.0248462
H	-3.0709599	2.1345054	-0.0252861
C	-3.2368928	0.0000000	0.0157893
C	-2.5141772	-1.1927472	-0.0248462
H	-3.0709599	-2.1345054	-0.0252861
O	-1.2626979	1.3454106	-0.0646298
O	-1.2626979	-1.3454106	-0.0646298
Ni	0.0000000	0.0000000	-0.0642318
C	-4.7664383	0.0000000	0.0187309
C	4.7664383	-0.0000000	0.0187309
C	-5.3195270	1.2330603	0.7399453
H	-5.1014105	2.1613856	0.2081917
H	-6.4071852	1.1581988	0.8122081
H	-4.9165233	1.3154813	1.7524715
C	-5.3195270	-1.2330603	0.7399453
H	-6.4071852	-1.1581988	0.8122081
H	-5.1014105	-2.1613856	0.2081917
H	-4.9165233	-1.3154813	1.7524715
C	-5.2842029	0.0000000	-1.4239503
H	-6.3783035	0.0000000	-1.4459609
H	-4.9327621	0.8826841	-1.9643009
H	-4.9327621	-0.8826841	-1.9643009
C	5.2842029	0.0000000	-1.4239503
H	4.9327621	0.8826841	-1.9643009
H	6.3783035	0.0000000	-1.4459609

H	4.9327621	-0.8826841	-1.9643009
C	5.3195270	1.2330603	0.7399453
H	6.4071852	1.1581988	0.8122081
H	5.1014105	2.1613856	0.2081917
H	4.9165233	1.3154813	1.7524715
C	5.3195270	-1.2330603	0.7399453
H	4.9165233	-1.3154813	1.7524715
H	5.1014105	-2.1613856	0.2081917
H	6.4071852	-1.1581988	0.8122081

7B

C	3.0557235	-1.2102211	-0.0323160
H	3.6652285	-2.1088473	-0.0330221
C	3.7331850	-0.0000000	0.0148456
C	3.0557235	1.2102211	-0.0323160
H	3.6652285	2.1088473	-0.0330221
C	-3.0557235	1.2102211	-0.0323160
H	-3.6652285	2.1088473	-0.0330221
C	-3.7331850	0.0000000	0.0148456
C	-3.0557235	-1.2102211	-0.0323160
H	-3.6652285	-2.1088473	-0.0330221
C	-5.2719009	0.0000000	0.0283027
C	5.2719009	0.0000000	0.0283027
C	-5.8265070	1.2320386	0.7490871
H	-5.6299685	2.1601625	0.2087514
H	-6.9117229	1.1448490	0.8367667
H	-5.4109768	1.3253122	1.7554765
C	-5.8265070	-1.2320386	0.7490871
H	-6.9117229	-1.1448490	0.8367667
H	-5.6299685	-2.1601625	0.2087514
H	-5.4109768	-1.3253122	1.7554765
C	-5.7747810	0.0000000	-1.4190448
H	-6.8683834	0.0000000	-1.4481501
H	-5.4195052	0.8830558	-1.9558946
H	-5.4195052	-0.8830558	-1.9558946

C	5.7747810	0.0000000	-1.4190448
H	5.4195052	0.8830558	-1.9558946
H	6.8683834	0.0000000	-1.4481501
H	5.4195052	-0.8830558	-1.9558946
C	5.8265070	1.2320386	0.7490871
H	6.9117229	1.1448490	0.8367667
H	5.6299685	2.1601625	0.2087514
H	5.4109768	1.3253122	1.7554765
C	5.8265070	-1.2320386	0.7490871
H	5.4109768	-1.3253122	1.7554765
H	5.6299685	-2.1601625	0.2087514
H	6.9117229	-1.1448490	0.8367667
S	-1.4427635	1.6150529	-0.0953233
S	-1.4427635	-1.6150529	-0.0953233
S	1.4427635	-1.6150529	-0.0953233
S	1.4427635	1.6150529	-0.0953233
Ni	0.0000000	0.0000000	-0.0860095

8A

C	2.5849538	-1.2292134	0.0000000
H	3.2120453	-2.1285012	0.0000000
C	3.2373874	0.0000000	0.0000000
H	4.3187910	0.0000000	0.0000000
C	2.5849538	1.2292134	-0.0000000
H	3.2120453	2.1285012	-0.0000000
O	1.3477604	-1.4591216	0.0000000
O	1.3477604	1.4591216	0.0000000
C	-2.5849538	1.2292134	0.0000000
H	-3.2120453	2.1285012	-0.0000000
C	-3.2373874	-0.0000000	0.0000000
H	-4.3187910	-0.0000000	0.0000000
C	-2.5849538	-1.2292134	-0.0000000
H	-3.2120453	-2.1285012	0.0000000
O	-1.3477604	1.4591216	-0.0000000
O	-1.3477604	-1.4591216	0.0000000

Pd	0.0000000	0.0000000	0.0000000

8B

C	-3.1106263	0.0000000	-1.2433562
H	-3.7872135	0.0000000	-2.0963160
C	-3.7197453	0.0000000	0.0001513
H	-4.8046387	0.0000000	0.0001211
C	-3.1102845	0.0000000	1.2434892
H	-3.7862980	0.0000000	2.0969129
C	3.1102845	0.0000000	1.2434892
H	3.7862980	0.0000000	2.0969129
C	3.7197453	0.0000000	0.0001513
H	4.8046387	0.0000000	0.0001211
C	3.1106263	0.0000000	-1.2433562
H	3.7872135	-0.0000000	-2.0963160
S	-1.5200471	0.0000000	1.7207674
S	-1.5206117	0.0000000	-1.7214928
S	1.5200471	0.0000000	1.7207674
S	1.5206117	0.0000000	-1.7214928
Pd	0.0000000	0.0000000	-0.0005537

9A

C	2.5840070	-1.2371457	0.0000000
H	3.2377972	-2.1159870	0.0000000
C	3.2123443	0.0000000	0.0000000
C	2.5840070	1.2371457	0.0000000
H	3.2377972	2.1159870	-0.0000000
O	1.3446656	-1.4612241	-0.0000000
O	1.3446656	1.4612241	0.0000000
C	-2.5840070	1.2371457	0.0000000
H	-3.2377972	2.1159870	0.0000000
C	-3.2123443	0.0000000	0.0000000
C	-2.5840070	-1.2371457	0.0000000
H	-3.2377972	-2.1159870	-0.0000000
O	-1.3446656	1.4612241	0.0000000

O	-1.3446656	-1.4612241	0.0000000
F	4.5647432	-0.0000000	0.0000000
F	-4.5647432	0.0000000	-0.0000000
Pd	0.0000000	0.0000000	0.0000000

9B

C	3.1038595	-0.0000000	-1.2485412
H	3.8110962	0.0000000	-2.0756528
C	3.6874464	0.0000000	0.0000241
C	3.1036615	-0.0000000	1.2485906
H	3.8100209	0.0000000	2.0765412
C	-3.1036615	-0.0000000	1.2485906
H	-3.8100209	0.0000000	2.0765412
C	-3.6874464	0.0000000	0.0000241
C	-3.1038595	0.0000000	-1.2485412
H	-3.8110962	0.0000000	-2.0756528
F	5.0414153	-0.0000000	0.0001698
F	-5.0414153	0.0000000	0.0001698
S	1.5139993	0.0000000	1.7249258
S	1.5144109	-0.0000000	-1.7257887
S	-1.5139993	0.0000000	1.7249258
S	-1.5144109	0.0000000	-1.7257887
Pd	0.0000000	0.0000000	-0.0005377

10A

C	-0.49079752	-1.19631900	0.00000000
H	0.13787775	-2.09305747	0.00000000
C	0.17156623	0.04402273	0.00000000
C	-0.49079752	1.28436446	0.00000000
H	0.13787775	2.18110293	0.00000000
O	-1.72362864	-1.40543503	0.00000000
O	-1.72362864	1.49348049	0.00000000
C	-5.66413380	1.28436446	0.00000000
H	-6.29280907	2.18110293	0.00000000
C	-6.32649755	0.04402273	0.00000000

C	-5.66413380	-1.19631900	0.00000000
H	-6.29280907	-2.09305747	0.00000000
O	-4.43130268	1.49348049	0.00000000
O	-4.43130268	-1.40543503	0.00000000
Pd	-3.07746566	0.04402273	0.00000000
C	-7.74846349	0.04402273	0.00000000
C	1.59353217	0.04402273	0.00000000
N	-8.90097133	0.04402273	0.00000000
N	2.74604001	0.04402273	0.00000000

10B

C	-0.93655596	0.40785498	0.00000000
H	-0.25723208	-0.44258684	-0.00002699
C	-0.31707356	1.65944172	-0.00007514
C	-0.93655596	2.91102847	0.00000000
H	-0.25723208	3.76147029	-0.00002699
C	-7.14738762	2.91102847	0.00000000
H	-7.82671151	3.76147029	-0.00002699
C	-7.76687003	1.65944172	-0.00007514
C	-7.14738762	0.40785498	0.00000000
H	-7.82671151	-0.44258684	-0.00002699
S	-2.52081052	3.37608955	0.00013388
S	-2.52081052	-0.05720610	0.00013388
S	-5.56313307	-0.05720610	0.00013388
S	-5.56313307	3.37608955	0.00013388
Pd	-4.04197179	1.65944172	0.00013494
C	-9.19677257	1.65944172	-0.00021432
C	1.11282899	1.65944172	-0.00021432
N	-10.34896713	1.65944172	-0.00032756
N	2.26502354	1.65944172	-0.00032756

11A

C	2.5886356	-1.2443862	0.0000210
H	3.2288256	-2.1294941	-0.0000020
C	3.2323912	0.0000000	-0.0000183

C	2.5886356	1.2443862	0.0000210
H	3.2288256	2.1294941	-0.0000020
O	1.3547005	-1.4484440	0.0000804
O	1.3547005	1.4484440	0.0000804
C	-2.5886356	1.2443862	0.0000210
H	-3.2288256	2.1294941	-0.0000020
C	-3.2323912	0.0000000	-0.0000183
C	-2.5886356	-1.2443862	0.0000210
H	-3.2288256	-2.1294941	-0.0000020
O	-1.3547005	1.4484440	0.0000804
O	-1.3547005	-1.4484440	0.0000804
N	-4.6814706	0.0000000	-0.0000660
N	4.6814706	0.0000000	-0.0000660
O	-5.2473239	-1.0746222	-0.0000806
O	-5.2473239	1.0746222	-0.0000806
O	5.2473239	-1.0746222	-0.0000806
O	5.2473239	1.0746222	-0.0000806
Pd	0.0000000	0.0000000	0.0000933

11B

C	3.1214047	-1.2536875	0.0000233
H	3.8364951	-2.0895422	-0.0000749
C	3.7492493	0.0000000	-0.0000302
C	3.1214047	1.2536875	0.0000233
H	3.8364951	2.0895422	-0.0000749
C	-3.1214047	1.2536875	0.0000233
H	-3.8364951	2.0895422	-0.0000749
C	-3.7492493	0.0000000	-0.0000302
C	-3.1214047	-1.2536875	0.0000233
H	-3.8364951	-2.0895422	-0.0000749
N	-5.0995222	0.0000000	-0.0001151
N	5.0995222	0.0000000	-0.0001151
O	-5.8060740	-1.0675620	-0.0001483
O	-5.8060740	1.0675620	-0.0001483
O	5.8060740	-1.0675620	-0.0001483

O	5.8060740	1.0675620	-0.0001483
S	1.5463774	1.6810557	0.0002145
S	1.5463774	-1.6810557	0.0002145
S	-1.5463774	-1.6810557	0.0002145
S	-1.5463774	1.6810557	0.0002145
Pd	0.0000000	0.0000000	0.0002322

12A

C	2.5997393	-1.2179382	-0.0000061
H	3.2184018	-2.1252015	0.0000210
C	3.2794034	0.0000000	0.0000916
C	2.5997393	1.2179382	-0.0000061
H	3.2184018	2.1252015	0.0000210
O	1.3551492	-1.4406886	-0.0000515
O	1.3551492	1.4406886	-0.0000515
C	-2.5997393	1.2179382	-0.0000061
H	-3.2184018	2.1252015	0.0000210
C	-3.2794034	-0.0000000	0.0000916
C	-2.5997393	-1.2179382	-0.0000061
H	-3.2184018	-2.1252015	0.0000210
O	-1.3551492	1.4406886	-0.0000515
O	-1.3551492	-1.4406886	-0.0000515
N	-4.6799062	0.0000000	0.0020193
N	4.6799062	0.0000000	0.0020193
H	-5.1928616	0.8568038	-0.0010215
H	-5.1928616	-0.8568038	-0.0010215
H	5.1928616	0.8568038	-0.0010215
H	5.1928616	-0.8568038	-0.0010215
Pd	0.0000000	0.0000000	0.0000103

12B

C	3.0314564	-1.2797943	0.0000007
H	3.7215163	-2.1191905	-0.0001902
C	3.6291939	-0.0000000	-0.0000616
C	3.0314564	1.2797943	0.0000007

H	3.7215163	2.1191905	-0.0001902
C	-3.0314564	1.2797943	0.0000007
H	-3.7215163	2.1191905	-0.0001902
C	-3.6291939	0.0000000	-0.0000616
C	-3.0314564	-1.2797943	0.0000007
H	-3.7215163	-2.1191905	-0.0001902
N	-4.9853786	0.0000000	-0.0002140
N	4.9853786	0.0000000	-0.0002140
H	-5.4992454	0.8614658	-0.0001337
H	-5.4992454	-0.8614658	-0.0001337
H	5.4992454	0.8614658	-0.0001337
H	5.4992454	-0.8614658	-0.0001337
S	1.4211666	1.7545699	0.0003422
S	1.4211666	-1.7545699	0.0003422
S	-1.4211666	1.7545699	0.0003422
S	-1.4211666	-1.7545699	0.0003422
Pd	0.0000000	0.0000000	0.0004753

13A

C	2.6488937	-1.0868676	-0.0004211
H	3.3237877	-1.9523622	0.0008909
C	3.2540537	0.1707814	-0.0003687
C	2.5201653	1.3609201	-0.0021229
H	3.1175527	2.2800854	-0.0026339
O	1.4246613	-1.3817166	-0.0014549
O	1.2727205	1.5222121	-0.0031582
C	-2.6488937	1.0868676	-0.0004211
H	-3.3237877	1.9523622	0.0008909
C	-3.2540537	-0.1707814	-0.0003687
C	-2.5201653	-1.3609201	-0.0021229
H	-3.1175527	-2.2800854	-0.0026339
O	-1.4246613	1.3817166	-0.0014549
O	-1.2727205	-1.5222121	-0.0031582
N	-4.6747766	-0.2888907	0.0016583
N	4.6747766	0.2888907	0.0016583

C	-5.3202521	0.1851431	1.2066005
H	-5.3001822	1.2836477	1.3154010
H	-6.3681429	-0.1269120	1.2075513
H	-4.8330445	-0.2498570	2.0805857
C	-5.3223846	0.1794449	-1.2043525
H	-4.8448790	-0.2687355	-2.0770240
H	-6.3732513	-0.1221526	-1.1959849
H	-5.2921583	1.2765713	-1.3239305
C	5.3202521	-0.1851431	1.2066005
H	5.3001822	-1.2836477	1.3154010
H	6.3681429	0.1269120	1.2075513
H	4.8330445	0.2498570	2.0805857
C	5.3223846	-0.1794449	-1.2043525
H	4.8448790	0.2687355	-2.0770240
H	6.3732513	0.1221526	-1.1959849
H	5.2921583	-1.2765713	-1.3239305
Pd	0.0000000	-0.0000000	-0.0024724

13B

C	3.1731661	-1.0680669	0.0015412
H	3.8892083	-1.8899316	0.0042107
C	3.7408775	0.1992719	-0.0002315
C	3.0457685	1.4016012	-0.0037678
H	3.6921347	2.2777108	-0.0054463
C	-3.1731661	1.0680669	0.0015412
H	-3.8892083	1.8899316	0.0042107
C	-3.7408775	-0.1992719	-0.0002315
C	-3.0457685	-1.4016012	-0.0037678
H	-3.6921347	-2.2777108	-0.0054463
N	-5.1705571	-0.3130258	0.0018726
N	5.1705571	0.3130258	0.0018726
C	-5.7971541	0.1889152	1.2062291
H	-5.7451534	1.2871698	1.3071744
H	-6.8526194	-0.0938877	1.2088794
H	-5.3210682	-0.2544260	2.0820778

C	-5.7994493	0.1817175	-1.2041680
H	-5.3320362	-0.2743748	-2.0782349
H	-6.8572981	-0.0920691	-1.1981866
H	-5.7385087	1.2784037	-1.3163679
C	5.7971541	-0.1889152	1.2062291
H	5.7451534	-1.2871698	1.3071744
H	6.8526194	0.0938877	1.2088794
H	5.3210682	0.2544260	2.0820778
C	5.7994493	-0.1817175	-1.2041680
H	5.3320362	0.2743748	-2.0782349
H	6.8572981	0.0920691	-1.1981866
H	5.7385087	-1.2784037	-1.3163679
S	-1.4319440	-1.7920992	-0.0053274
S	-1.6112525	1.6307077	0.0008901
S	1.6112525	-1.6307077	0.0008901
S	1.4319440	1.7920992	-0.0053274
Pd	0.0000000	-0.0000000	-0.0022897

14A

C	2.6051283	-1.2119351	-0.0273263
H	3.1996819	-2.1297765	-0.0282299
C	3.2999925	0.0000000	0.0153136
C	2.6051283	1.2119351	-0.0273263
H	3.1996819	2.1297765	-0.0282299
O	1.3639733	-1.4310241	-0.0678339
O	1.3639733	1.4310241	-0.0678339
C	-2.6051283	1.2119351	-0.0273263
H	-3.1996819	2.1297765	-0.0282299
C	-3.2999925	0.0000000	0.0153136
C	-2.6051283	-1.2119351	-0.0273263
H	-3.1996819	-2.1297765	-0.0282299
O	-1.3639733	1.4310241	-0.0678339
O	-1.3639733	-1.4310241	-0.0678339
C	-4.8349496	0.0000000	0.0190403
C	4.8349496	0.0000000	0.0190403

C	-5.3919470	1.2312655	0.7419798
H	-5.1914900	2.1609748	0.2057375
H	-6.4779108	1.1460049	0.8266197
H	-4.9787587	1.3206203	1.7498645
C	-5.3919470	-1.2312655	0.7419798
H	-6.4779108	-1.1460049	0.8266197
H	-5.1914900	-2.1609748	0.2057375
H	-4.9787587	-1.3206203	1.7498645
C	-5.3501195	0.0000000	-1.4243824
H	-6.4441660	0.0000000	-1.4506817
H	-4.9970054	0.8827036	-1.9636576
H	-4.9970054	-0.8827036	-1.9636576
C	5.3501195	0.0000000	-1.4243824
H	4.9970054	0.8827036	-1.9636576
H	6.4441660	0.0000000	-1.4506817
H	4.9970054	-0.8827036	-1.9636576
C	5.3919470	1.2312655	0.7419798
H	6.4779108	1.1460049	0.8266197
H	5.1914900	2.1609748	0.2057375
H	4.9787587	1.3206203	1.7498645
C	5.3919470	-1.2312655	0.7419798
H	4.9787587	-1.3206203	1.7498645
H	5.1914900	-2.1609748	0.2057375
H	6.4779108	-1.1460049	0.8266197
Pd	0.0000000	0.0000000	-0.0671951

14B

C	3.0595321	-1.2560408	0.0566776
H	3.7051959	-2.1226121	0.0689577
C	3.6777533	0.0000000	0.0751444
C	3.0595321	1.2560408	0.0566776
H	3.7051959	2.1226121	0.0689577
C	-3.0595321	1.2560408	0.0566776
H	-3.7051959	2.1226121	0.0689577
C	-3.6777533	0.0000000	0.0751444

C	-3.0595321	-1.2560408	0.0566776
H	-3.7051959	-2.1226121	0.0689577
C	-5.2156073	0.0000000	0.0235140
C	5.2156073	-0.0000000	0.0235140
C	-5.8293832	1.2322834	0.6973900
H	-5.6257794	2.1572129	0.1563305
H	-6.9151817	1.1184702	0.7247761
H	-5.4738987	1.3465781	1.7238055
C	-5.8293832	-1.2322834	0.6973900
H	-6.9151817	-1.1184702	0.7247761
H	-5.6257794	-2.1572129	0.1563305
H	-5.4738987	-1.3465781	1.7238055
C	-5.6071319	0.0000000	-1.4629661
H	-6.6967446	-0.0000000	-1.5588878
H	-5.2159523	0.8845688	-1.9695970
H	-5.2159523	-0.8845688	-1.9695970
C	5.6071319	-0.0000000	-1.4629661
H	5.2159523	0.8845688	-1.9695970
H	6.6967446	0.0000000	-1.5588878
H	5.2159523	-0.8845688	-1.9695970
C	5.8293832	1.2322834	0.6973900
H	6.9151817	1.1184702	0.7247761
H	5.6257794	2.1572129	0.1563305
H	5.4738987	1.3465781	1.7238055
C	5.8293832	-1.2322834	0.6973900
H	5.4738987	-1.3465781	1.7238055
H	5.6257794	-2.1572129	0.1563305
H	6.9151817	-1.1184702	0.7247761
S	-1.4339709	1.7314204	0.0035514
S	-1.4339709	-1.7314204	0.0035514
S	1.4339709	-1.7314204	0.0035514
S	1.4339709	1.7314204	0.0035514
Pd	0.0000000	0.0000000	-0.0011764

15A

C	2.5808626	-1.2322053	-0.0000000
H	3.2046708	-2.1313671	-0.0000000
C	3.2257849	0.0000000	0.0000000
H	4.3073354	0.0000000	0.0000000
C	2.5808626	1.2322053	-0.0000000
H	3.2046708	2.1313671	0.0000000
O	1.3401131	-1.4667361	0.0000000
O	1.3401131	1.4667361	0.0000000
C	-2.5808626	1.2322053	0.0000000
H	-3.2046708	2.1313671	-0.0000000
C	-3.2257849	0.0000000	0.0000000
H	-4.3073354	0.0000000	0.0000000
C	-2.5808626	-1.2322053	-0.0000000
H	-3.2046708	-2.1313671	0.0000000
O	-1.3401131	1.4667361	0.0000000
O	-1.3401131	-1.4667361	0.0000000
Pt	0.0000000	0.0000000	0.0000000

15B

C	3.1730538	-1.2349258	0.0000000
H	3.7661648	-2.1687481	0.0000000
C	3.7711122	-0.0000000	0.0000000
H	4.8560049	0.0000000	0.0000000
C	3.1730538	1.2349258	-0.0000000
H	3.7661648	2.1687481	0.0000000
C	-3.1730538	1.2349258	0.0000000
H	-3.7661648	2.1687481	0.0000000
C	-3.7711122	0.0000000	0.0000000
H	-4.8560049	0.0000000	0.0000000
C	-3.1730538	-1.2349258	0.0000000
H	-3.7661648	-2.1687481	-0.0000000
Pt	0.0000000	0.0000000	0.0000000
S	1.5667886	1.7937389	-0.0000000
S	1.5667886	-1.7937389	0.0000000
S	-1.5667886	1.7937389	-0.0000000

S	-1.5667886	-1.7937389	-0.0000000

16A

C	2.5792976	-1.2399119	-0.0000000
H	3.2300493	-2.1186361	0.0000000
C	3.2009632	0.0000000	0.0000000
C	2.5792976	1.2399119	0.0000000
H	3.2300493	2.1186361	0.0000000
O	1.3365021	-1.4688983	0.0000000
O	1.3365021	1.4688983	0.0000000
C	-2.5792976	1.2399119	-0.0000000
H	-3.2300493	2.1186361	0.0000000
C	-3.2009632	-0.0000000	0.0000000
C	-2.5792976	-1.2399119	0.0000000
H	-3.2300493	-2.1186361	0.0000000
O	-1.3365021	1.4688983	0.0000000
O	-1.3365021	-1.4688983	0.0000000
F	4.5532105	0.0000000	0.0000000
F	-4.5532105	0.0000000	0.0000000
Pt	0.0000000	0.0000000	0.0000000

16B

C	3.1865625	-1.2488530	0.0000000
H	3.8306157	-2.1398172	0.0000000
C	3.7613698	-0.0000000	0.0000000
C	3.1865625	1.2488530	0.0000000
H	3.8306157	2.1398172	0.0000000
C	-3.1865625	1.2488530	0.0000000
H	-3.8306157	2.1398172	0.0000000
C	-3.7613698	0.0000000	0.0000000
C	-3.1865625	-1.2488530	0.0000000
H	-3.8306157	-2.1398172	0.0000000
F	5.1030899	0.0000000	-0.0000000
F	-5.1030899	0.0000000	0.0000000
Pt	-0.0000000	0.0000000	0.0000000

S	1.5731902	1.7805340	-0.0000000
S	1.5731902	-1.7805340	0.0000000
S	-1.5731902	1.7805340	0.0000000
S	-1.5731902	-1.7805340	-0.0000000

17A

C	-0.36253779	-0.58912386	0.00000000
H	-0.98829624	-0.58912386	-0.89651463
C	-1.01757456	-0.58912386	1.24317600
C	-0.36252985	-0.58912386	2.48633666
H	-0.98829836	-0.58912386	3.38286135
O	0.87379119	-0.58912386	-0.21405484
O	0.87378325	-0.58912386	2.70039944
C	4.80236392	-0.58912386	2.48633666
H	5.42813242	-0.58912386	3.38286135
C	5.45740863	-0.58912386	1.24317600
C	4.80237186	-0.58912386	0.00000000
H	5.42813031	-0.58912386	-0.89651463
O	3.56605082	-0.58912386	2.70039944
O	3.56604288	-0.58912386	-0.21405484
Pt	2.21991703	-0.58912386	1.24317018
C	6.88008261	-0.58912386	1.24317124
C	-2.44024854	-0.58912386	1.24317124
N	8.03244280	-0.58912386	1.24317177
N	-3.59260873	-0.58912386	1.24317177

17B

C	-1.23867079	0.01510574	0.00000000
H	-2.09016143	0.01510574	0.67705001
C	0.01419392	0.01510574	0.61599248
C	1.26705863	0.01510574	0.00000000
H	2.11854927	0.01510574	0.67705001
C	1.26707926	0.01510574	-6.18218148
H	2.11854133	0.01510574	-6.85925900
C	0.01419392	0.01510574	-6.79822582

C	-1.23869143	0.01510574	-6.18218148
H	-2.09015350	0.01510574	-6.85925900
Pt	0.01419392	0.01510574	-3.09110159
S	1.73746228	0.01510574	-1.58262045
S	-1.70907444	0.01510574	-1.58262045
S	-1.70906862	0.01510574	-4.59960494
S	1.73745645	0.01510574	-4.59960494
C	0.01419392	0.01510574	-8.22815059
C	0.01419392	0.01510574	2.04594530
N	0.01419392	0.01510574	-9.38037319
N	0.01419392	0.01510574	3.19817372

18A

C	2.5855452	-1.2466163	0.0000186
H	3.2219476	-2.1321883	0.0000011
C	3.2229364	0.0000000	-0.0000129
C	2.5855452	1.2466163	0.0000186
H	3.2219476	2.1321883	0.0000011
O	1.3480230	-1.4548580	0.0000775
O	1.3480230	1.4548580	0.0000775
C	-2.5855452	1.2466163	0.0000186
H	-3.2219476	2.1321883	0.0000011
C	-3.2229364	0.0000000	-0.0000129
C	-2.5855452	-1.2466163	0.0000186
H	-3.2219476	-2.1321883	0.0000011
O	-1.3480230	1.4548580	0.0000775
O	-1.3480230	-1.4548580	0.0000775
N	-4.6739188	0.0000000	-0.0000625
N	4.6739188	0.0000000	-0.0000625
O	-5.2390852	-1.0745686	-0.0000815
O	-5.2390852	1.0745686	-0.0000815
O	5.2390852	-1.0745686	-0.0000815
O	5.2390852	1.0745686	-0.0000815
Pt	0.0000000	0.0000000	0.0000880

18B

C	3.0820143	-1.2626984	0.0000126
H	3.7888567	-2.1001927	-0.0001209
C	3.7023978	0.0000000	-0.0000235
C	3.0820143	1.2626984	0.0000126
H	3.7888567	2.1001927	-0.0001209
C	-3.0820143	1.2626984	0.0000126
H	-3.7888567	2.1001927	-0.0001209
C	-3.7023978	0.0000000	-0.0000235
C	-3.0820143	-1.2626984	0.0000126
H	-3.7888567	-2.1001927	-0.0001209
N	-5.0458761	0.0000000	-0.0000858
N	5.0458761	0.0000000	-0.0000858
O	-5.7839816	-1.0517920	-0.0001143
O	-5.7839816	1.0517920	-0.0001143
O	5.7839816	-1.0517920	-0.0001143
O	5.7839816	1.0517920	-0.0001143
Pt	0.0000000	0.0000000	0.0002184
S	1.5091600	1.7032298	0.0002227
S	1.5091600	-1.7032298	0.0002227
S	-1.5091600	-1.7032298	0.0002227
S	-1.5091600	1.7032298	0.0002227

19A

C	2.5954382	-1.2214379	-0.0000016
H	3.2116975	-2.1278356	0.0000178
C	3.2681666	0.0000000	0.0000885
C	2.5954382	1.2214379	-0.0000016
H	3.2116975	2.1278356	0.0000178
O	1.3476196	-1.4501400	-0.0000297
O	1.3476196	1.4501400	-0.0000297
C	-2.5954382	1.2214379	-0.0000016
H	-3.2116975	2.1278356	0.0000178
C	-3.2681666	-0.0000000	0.0000885
C	-2.5954382	-1.2214379	-0.0000016

H	-3.2116975	-2.1278356	0.0000178
O	-1.3476196	1.4501400	-0.0000297
O	-1.3476196	-1.4501400	-0.0000297
N	-4.6683027	0.0000000	0.0016787
N	4.6683027	0.0000000	0.0016787
H	-5.1814828	0.8566765	-0.0008738
H	-5.1814828	-0.8566765	-0.0008738
H	5.1814828	0.8566765	-0.0008738
H	5.1814828	-0.8566765	-0.0008738
Pt	0.0000000	0.0000000	0.0000149

19B

C	3.1353458	-1.2616342	0.0000444
H	3.7828211	-2.1321913	-0.0006613
C	3.7526953	0.0000000	0.0002198
C	3.1353458	1.2616342	0.0000444
H	3.7828211	2.1321913	-0.0006613
C	-3.1353458	1.2616342	0.0000444
H	-3.7828211	2.1321913	-0.0006613
C	-3.7526953	0.0000000	0.0002198
C	-3.1353458	-1.2616342	0.0000444
H	-3.7828211	-2.1321913	-0.0006613
N	-5.1079599	0.0000000	0.0002359
N	5.1079599	0.0000000	0.0002359
H	-5.6215576	0.8614210	-0.0002623
H	-5.6215576	-0.8614210	-0.0002623
H	5.6215576	0.8614210	-0.0002623
H	5.6215576	-0.8614210	-0.0002623
Pt	-0.0000000	0.0000000	0.0001941
S	1.5033132	1.6789055	0.0006028
S	1.5033132	-1.6789055	0.0006028
S	-1.5033132	1.6789055	0.0006028
S	-1.5033132	-1.6789055	0.0006028

20A

34

C	2.6455115	-1.0900084	-0.0006135
H	3.3163927	-1.9559408	0.0005258
C	3.2436100	0.1701061	-0.0003824
C	2.5169507	1.3633812	-0.0019087
H	3.1120064	2.2818237	-0.0022801
O	1.4178192	-1.3896692	-0.0016674
O	1.2655799	1.5296674	-0.0028638
C	-2.6455115	1.0900084	-0.0006135
H	-3.3163927	1.9559408	0.0005258
C	-3.2436100	-0.1701061	-0.0003824
C	-2.5169507	-1.3633812	-0.0019087
H	-3.1120064	-2.2818237	-0.0022801
O	-1.4178192	1.3896692	-0.0016674
O	-1.2655799	-1.5296674	-0.0028638
N	-4.6650663	-0.2897568	0.0015822
N	4.6650663	0.2897568	0.0015822
C	-5.3099038	0.1849821	1.2067550
H	-5.2919477	1.2836347	1.3137951
H	-6.3570407	-0.1293308	1.2097068
H	-4.8208619	-0.2480828	2.0804621
C	-5.3120810	0.1792453	-1.2045492
H	-4.8329246	-0.2670973	-2.0771514
H	-6.3622063	-0.1245937	-1.1977261
H	-5.2835062	1.2765704	-1.3224814
C	5.3099038	-0.1849821	1.2067550
H	5.2919477	-1.2836347	1.3137951
H	6.3570407	0.1293308	1.2097068
H	4.8208619	0.2480828	2.0804621
C	5.3120810	-0.1792453	-1.2045492
H	4.8329246	0.2670973	-2.0771514
H	6.3622063	0.1245937	-1.1977261
H	5.2835062	-1.2765704	-1.3224814
Pt	0.0000000	0.0000000	-0.0024062

20B

35

C	-1.2247892	-3.1219982	-0.0000000
H	-2.0652086	-3.8133393	0.0000000
C	0.0715262	-3.6317006	-0.0000000
C	1.3356856	-3.0591950	0.0000000
H	2.2123604	-3.7042397	0.0000000
C	1.2247892	3.1219982	0.0000000
H	2.0652086	3.8133393	0.0000000
C	-0.0715262	3.6317006	0.0000000
C	-1.3356856	3.0591950	0.0000000
H	-2.2123604	3.7042397	0.0000000
N	-0.0896110	5.0449269	0.0000000
N	0.0896110	-5.0449269	0.0000000
C	0.1317362	5.7245055	1.2311975
H	1.2112825	5.7585413	1.4643318
H	-0.2370429	6.7519197	1.1799730
H	-0.3580780	5.1753710	2.0359585
C	0.1317362	5.7245055	-1.2311975
H	-0.3580780	5.1753710	-2.0359585
H	-0.2370429	6.7519197	-1.1799730
H	1.2112825	5.7585413	-1.4643318
C	-0.1317362	-5.7245055	1.2311975
H	-1.2112825	-5.7585413	1.4643318
H	0.2370429	-6.7519197	1.1799730
H	0.3580780	-5.1753710	2.0359585
C	-0.1317362	-5.7245055	-1.2311975
H	0.3580780	-5.1753710	-2.0359585
H	0.2370429	-6.7519197	-1.1799730
H	-1.2112825	-5.7585413	-1.4643318
Pt	0.0000000	0.0000000	0.0000000
S	-1.8930702	1.4218617	0.0000000
S	1.8312976	1.4985993	0.0000000
S	-1.8312976	-1.4985993	0.0000000
S	1.8930702	-1.4218617	0.0000000

21A

36

C	2.6014886	-1.2151770	-0.0262029
H	3.1934831	-2.1322692	-0.0288711
C	3.2888443	0.0000000	0.0142840
C	2.6014886	1.2151770	-0.0262029
H	3.1934831	2.1322692	-0.0288711
O	1.3568823	-1.4402774	-0.0629523
O	1.3568823	1.4402774	-0.0629523
C	-2.6014886	1.2151770	-0.0262029
H	-3.1934831	2.1322692	-0.0288711
C	-3.2888443	-0.0000000	0.0142840
C	-2.6014886	-1.2151770	-0.0262029
H	-3.1934831	-2.1322692	-0.0288711
O	-1.3568823	1.4402774	-0.0629523
O	-1.3568823	-1.4402774	-0.0629523
C	-4.8246117	0.0000000	0.0177405
C	4.8246117	0.0000000	0.0177405
C	-5.3825255	1.2305724	0.7409991
H	-5.1846418	2.1609760	0.2051560
H	-6.4681389	1.1423896	0.8256483
H	-4.9696339	1.3200579	1.7489093
C	-5.3825255	-1.2305724	0.7409991
H	-6.4681389	-1.1423896	0.8256483
H	-5.1846418	-2.1609760	0.2051560
H	-4.9696339	-1.3200579	1.7489093
C	-5.3395441	0.0000000	-1.4255115
H	-6.4335274	-0.0000000	-1.4505944
H	-4.9869081	0.8825795	-1.9651297
H	-4.9869081	-0.8825795	-1.9651297
C	5.3395441	0.0000000	-1.4255115
H	4.9869081	0.8825795	-1.9651297
H	6.4335274	0.0000000	-1.4505944
H	4.9869081	-0.8825795	-1.9651297
C	5.3825255	1.2305724	0.7409991
H	6.4681389	1.1423896	0.8256483
H	5.1846418	2.1609760	0.2051560

H	4.9696339	1.3200579	1.7489093
C	5.3825255	-1.2305724	0.7409991
H	4.9696339	-1.3200579	1.7489093
H	5.1846418	-2.1609760	0.2051560
H	6.4681389	-1.1423896	0.8256483
Pt	-0.0000000	0.0000000	-0.0620643

21B

C	-1.2234801	-0.0318454	3.1165563
H	-2.1036666	-0.0324779	3.7521013
C	-0.0000185	0.0154743	3.7737139
C	1.2234122	-0.0313906	3.1163819
H	2.1038536	-0.0321885	3.7515367
C	1.2234122	-0.0313906	-3.1163819
H	2.1038536	-0.0321885	-3.7515367
C	-0.0000185	0.0154743	-3.7737139
C	-1.2234801	-0.0318454	-3.1165563
H	-2.1036666	-0.0324779	-3.7521013
C	-0.0000683	0.0282641	-5.3164393
C	-0.0000683	0.0282641	5.3164393
C	1.2307932	0.7485147	-5.8756505
H	2.1593527	0.2049744	-5.6907692
H	1.1358750	0.8413827	-6.9596887
H	1.3302748	1.7529304	-5.4568276
C	-1.2308027	0.7488245	-5.8757345
H	-1.1357048	0.8416264	-6.9597949
H	-2.1594474	0.2053441	-5.6909228
H	-1.3300874	1.7532688	-5.4567740
C	-0.0000455	-1.4196309	-5.8170944
H	-0.0000711	-1.4508180	-6.9106769
H	0.8828713	-1.9559196	-5.4604889
H	-0.8830081	-1.9557895	-5.4605127
C	-0.0000455	-1.4196309	5.8170944
H	0.8828713	-1.9559196	5.4604889
H	-0.0000711	-1.4508180	6.9106769

H	-0.8830081	-1.9557895	5.4605127
C	1.2307932	0.7485147	5.8756505
H	1.1358750	0.8413827	6.9596887
H	2.1593527	0.2049744	5.6907692
H	1.3302748	1.7529304	5.4568276
C	-1.2308027	0.7488245	5.8757345
H	-1.3300874	1.7532688	5.4567740
H	-2.1594474	0.2053441	5.6909228
H	-1.1357048	0.8416264	6.9597949
Pt	0.0000155	-0.0873029	0.0000000
S	1.7009308	-0.0930369	-1.5254577
S	-1.7009709	-0.0938555	-1.5255337
S	-1.7009709	-0.0938555	1.5255337
S	1.7009308	-0.0930369	1.5254577

Cartesian coordinates of the optimized pyridine dimers

1A:py

C	2.5031934	-1.2102917	1.0503173
H	3.0956882	-2.1337376	1.0491747
C	3.1860078	-0.0000701	1.0101867
H	4.2663932	-0.0000013	0.9766180
C	2.5031675	1.2101626	1.0502438
H	3.0956183	2.1335843	1.0504814
O	1.2581369	-1.3768652	1.0954963
O	1.2580596	1.3767482	1.0940237
C	-2.5031559	1.2103463	1.0503182
H	-3.0956491	2.1337064	1.0492831
C	-3.1859995	0.0000206	1.0099177
H	-4.2663329	0.0000105	0.9764638
C	-2.5031756	-1.2101323	1.0502711
H	-3.0955891	-2.1335866	1.0505547
O	-1.2580915	1.3768609	1.0953821
O	-1.2579802	-1.3767609	1.0939875
Ni	0.0000166	0.0000063	1.0636826
N	-0.0000239	-0.0000094	-1.5525761
C	0.0000222	-1.1400042	-2.2324203
C	-0.0000738	1.1400176	-2.2323972
C	0.0000137	-1.1930377	-3.6184000
H	0.0000618	-2.0459892	-1.6324513
C	-0.0000722	1.1930314	-3.6184068
H	-0.0001146	2.0459699	-1.6324478
C	-0.0000333	0.0000135	-4.3238619
H	0.0000420	-2.1482240	-4.1295717
H	-0.0000949	2.1482259	-4.1295569
H	-0.0000347	0.0000056	-5.4080316

1B:py

C	1.2322571	-3.0439929	-0.8172292
H	2.1034087	-3.6961675	-0.8129296

C	-0.0000041	-3.6695682	-0.7598146
H	-0.0000021	-4.7526334	-0.6996414
C	-1.2322641	-3.0439956	-0.8172436
H	-2.1034156	-3.6961726	-0.8129557
C	-1.2322622	3.0439876	-0.8172307
H	-2.1034151	3.6961653	-0.8129447
C	-0.0000058	3.6695575	-0.7597736
H	-0.0000015	4.7526244	-0.6996149
C	1.2322569	3.0439849	-0.8172305
H	2.1034063	3.6961604	-0.8129444
S	-1.6477132	-1.4382358	-0.9134138
S	1.6477025	-1.4382333	-0.9133976
S	-1.6477105	1.4382303	-0.9134525
S	1.6477003	1.4382274	-0.9134511
Ni	-0.0000059	-0.0000037	-0.8555123
N	0.0000007	0.0000063	1.8962171
C	-1.1383845	0.0000058	2.5767119
C	1.1383904	0.0000058	2.5767046
C	-1.1926717	0.0000062	3.9628009
H	-2.0476565	0.0000040	1.9806487
C	1.1926873	0.0000063	3.9627917
H	2.0476583	0.0000040	1.9806340
C	0.0000104	0.0000067	4.6688500
H	-2.1485867	0.0000060	4.4727946
H	2.1486056	0.0000062	4.4727805
H	0.0000149	0.0000079	5.7530629

2A:py

C	2.5017161	-1.2197181	0.8889483
H	3.1217983	-2.1234543	0.8860082
C	3.1567820	0.0000670	0.8387407
C	2.5015768	1.2197578	0.8886475
H	3.1217283	2.1234503	0.8866522
O	1.2553392	-1.3805226	0.9452957
O	1.2551755	1.3806903	0.9437668

C	-2.5017276	1.2196902	0.8889527
H	-3.1218108	2.1234363	0.8861020
C	-3.1568026	-0.0000422	0.8387118
C	-2.5015836	-1.2198087	0.8886384
H	-3.1217268	-2.1234587	0.8865355
O	-1.2553536	1.3805123	0.9451974
O	-1.2551737	-1.3806972	0.9439385
Ni	-0.0000041	-0.0000131	0.9110531
F	4.5074251	0.0000601	0.7784102
F	-4.5074216	-0.0000785	0.7783310
N	0.0000092	0.0000361	-1.6812692
C	-0.0002226	-1.1402154	-2.3617217
C	0.0002394	1.1402673	-2.3617510
C	-0.0002499	-1.1930216	-3.7474185
H	-0.0004006	-2.0468065	-1.7628025
C	0.0002570	1.1930325	-3.7474537
H	0.0004262	2.0468765	-1.7628647
C	-0.0000005	-0.0000082	-4.4528812
H	-0.0004689	-2.1483873	-4.2581106
H	0.0004782	2.1483781	-4.2581784
H	-0.0000047	-0.0000224	-5.5370240

2B:py

C	3.0373418	-1.2385714	0.7068573
H	3.7216050	-2.0840738	0.7010154
C	3.6339088	-0.0000066	0.6387401
C	3.0373371	1.2385570	0.7068207
H	3.7216102	2.0840447	0.7009478
C	-3.0373170	1.2385888	0.7068523
H	-3.7216081	2.0840392	0.7010049
C	-3.6339473	-0.0000069	0.6387364
C	-3.0373191	-1.2385973	0.7068295
H	-3.7216042	-2.0840692	0.7009593
F	4.9840753	-0.0000088	0.5410651
F	-4.9840612	-0.0000054	0.5410655

S	1.4332206	1.6530275	0.8181356
S	1.4332154	-1.6530333	0.8181947
S	-1.4332300	1.6530216	0.8181677
S	-1.4332234	-1.6530328	0.8181635
Ni	0.0000011	-0.0000024	0.7551640
N	-0.0000001	-0.0000204	-1.9815179
C	-0.0000002	1.1384566	-2.6624431
C	-0.0000004	-1.1384613	-2.6624795
C	-0.0000007	1.1926435	-4.0482735
H	-0.0000003	2.0482549	-2.0671744
C	-0.0000005	-1.1925871	-4.0483160
H	-0.0000001	-2.0482869	-2.0672394
C	-0.0000006	0.0000336	-4.7543923
H	-0.0000008	2.1486081	-4.5579203
H	-0.0000007	-2.1485475	-4.5579960
H	-0.0000007	0.0000355	-5.8385395

3A:py

C	2.4993647	-1.2224071	0.8532939
H	3.0968097	-2.1408261	0.8511041
C	3.1883896	0.0000260	0.7925665
C	2.4993630	1.2223823	0.8533936
H	3.0968120	2.1408476	0.8512115
O	1.2616374	-1.3705032	0.9191147
O	1.2616420	1.3705149	0.9193011
C	-2.4991667	1.2224060	0.8528780
H	-3.0966100	2.1408359	0.8502527
C	-3.1881708	-0.0000204	0.7920367
C	-2.4991554	-1.2223791	0.8528099
H	-3.0965954	-2.1408515	0.8505124
O	-1.2614708	1.3705146	0.9192402
O	-1.2614351	-1.3705225	0.9187451
Ni	0.0000938	0.0000128	0.8758233
C	-4.6046283	0.0000002	0.7097273
C	4.6048078	-0.0000089	0.7097790

N	5.7556678	-0.0000392	0.6379007
N	-5.7555242	0.0000054	0.6384736
N	-0.0002741	0.0000485	-1.6665246
C	-0.0002272	-1.1406922	-2.3483194
C	-0.0002461	1.1407464	-2.3483840
C	-0.0001374	-1.1930235	-3.7333470
H	-0.0002393	-2.0488823	-1.7519732
C	-0.0001597	1.1930012	-3.7334185
H	-0.0002700	2.0489691	-1.7520940
C	-0.0000958	-0.0000356	-4.4387091
H	-0.0000773	-2.1484645	-4.2435261
H	-0.0001191	2.1484089	-4.2436503
H	0.0000148	-0.0000641	-5.5225945

3B:py

C	-3.0346945	1.2409112	-0.6894382
H	-3.6923434	2.1073704	-0.6787780
C	-3.6656753	-0.0000171	-0.6008708
C	-3.0346938	-1.2409515	-0.6894464
H	-3.6923400	-2.1074097	-0.6787937
C	3.0343858	-1.2409346	-0.6890021
H	3.6920749	-2.1073703	-0.6790664
C	3.6653322	-0.0000222	-0.5998666
C	3.0343874	1.2408997	-0.6889912
H	3.6920773	2.1073282	-0.6790465
Ni	-0.0001559	-0.0000117	-0.7582964
C	5.0867586	-0.0000169	-0.4613808
C	-5.0871143	-0.0000167	-0.4624774
N	-6.2334928	-0.0000111	-0.3441285
N	6.2332371	-0.0000117	-0.3438274
S	1.4392474	-1.6443542	-0.8331461
S	1.4392507	1.6443292	-0.8331181
S	-1.4395307	1.6443639	-0.8331826
S	-1.4395266	-1.6443892	-0.8331994
N	0.0003341	0.0000425	1.9309110

C	0.0003196	-1.1387212	2.6130192
C	0.0003180	1.1387950	2.6130407
C	0.0002258	-1.1924964	3.9983261
H	0.0003920	-2.0499407	2.0202047
C	0.0002238	1.1925402	3.9983499
H	0.0003894	2.0500352	2.0202603
C	0.0001397	0.0000141	4.7043774
H	0.0002346	-2.1483966	4.5077786
H	0.0002310	2.1484350	4.5078072
H	0.0000080	0.0000072	5.7883357

4A:py

C	2.4981517	-1.2281178	0.7527522
H	3.1075959	-2.1349680	0.7472545
C	3.1655526	0.0000299	0.6738250
C	2.4982122	1.2281031	0.7513337
H	3.1077765	2.1349195	0.7446541
O	1.2608539	-1.3708327	0.8388037
O	1.2608538	1.3711818	0.8366019
C	-2.4982239	1.2282520	0.7513942
H	-3.1077753	2.1350532	0.7449023
C	-3.1655835	0.0000922	0.6738871
C	-2.4981643	-1.2279733	0.7525761
H	-3.1076377	-2.1348366	0.7469288
O	-1.2608767	1.3712388	0.8366317
O	-1.2608596	-1.3707974	0.8385049
Ni	-0.0000053	0.0001654	0.7930949
N	-4.6007739	-0.0000196	0.5595954
N	4.6007516	0.0000163	0.5594726
O	-5.1661030	-1.0756466	0.5086333
O	-5.1668518	1.0755084	0.5149655
O	5.1660076	-1.0755492	0.5065132
O	5.1668481	1.0755749	0.5167711
N	0.0000032	-0.0003869	-1.7401684
C	-0.0000313	1.1406726	-2.4219308

C	0.0000521	-1.1412142	-2.4223000
C	-0.0000166	1.1929320	-3.8068635
H	-0.0000750	2.0489833	-1.8258478
C	0.0000721	-1.1930124	-3.8072498
H	0.0000771	-2.0497360	-1.8265345
C	0.0000368	0.0000624	-4.5124030
H	-0.0000439	2.1484313	-4.3167217
H	0.0001235	-2.1483675	-4.3174088
H	0.0000533	0.0002410	-5.5963214

4B:py

C	3.0259135	-1.2416402	0.6231264
H	3.7042441	-2.0900741	0.5969087
C	3.6268325	0.0001458	0.5005461
C	3.0261346	1.2419517	0.6240674
H	3.7045347	2.0903634	0.5984281
C	-3.0260688	1.2418683	0.6236384
H	-3.7044725	2.0902635	0.5977065
C	-3.6268355	0.0000556	0.5005454
C	-3.0259797	-1.2417261	0.6234898
H	-3.7043073	-2.0901768	0.5975462
N	-5.0827689	0.0000059	0.2903706
N	5.0827595	-0.0000236	0.2903324
O	-5.6412010	-1.0735874	0.2039003
O	-5.6415635	1.0735654	0.2058362
O	5.6415485	-1.0736699	0.2068160
O	5.6411910	1.0734802	0.2028110
S	1.4396465	1.6458771	0.8285016
S	1.4394024	-1.6456320	0.8268661
S	-1.4394590	-1.6456211	0.8274837
S	-1.4395790	1.6458957	0.8277354
Ni	0.0000017	0.0001997	0.7496155
N	0.0000140	-0.0000943	-1.9268406
C	0.0000149	1.1388705	-2.6090286
C	0.0000020	-1.1390993	-2.6089712

C	0.0000058	1.1924033	-3.9942510
H	0.0000219	2.0501641	-2.0164622
C	-0.0000068	-1.1927041	-3.9941912
H	-0.0000000	-2.0503722	-2.0163706
C	-0.0000040	-0.0001685	-4.7002078
H	0.0000088	2.1483401	-4.5035689
H	-0.0000196	-2.1486675	-4.5034663
H	-0.0000107	-0.0001931	-5.7841458

5A:py

C	2.5099571	-1.2007739	1.0208913
H	3.1013385	-2.1272489	1.0235356
C	3.2089265	0.0007720	0.9600296
C	2.5093706	1.2018958	1.0233708
H	3.1004109	2.1285446	1.0287425
O	1.2611265	-1.3639053	1.0830879
O	1.2604097	1.3642781	1.0837526
C	-2.5099266	1.2006339	1.0208470
H	-3.1012180	2.1271757	1.0234577
C	-3.2089533	-0.0008200	0.9599205
C	-2.5094673	-1.2020305	1.0233497
H	-3.1005932	-2.1286012	1.0288248
O	-1.2610547	1.3637080	1.0832062
O	-1.2605217	-1.3644724	1.0837159
Ni	-0.0000133	-0.0001234	1.0574603
N	-4.6354700	-0.0018165	0.9664315
N	4.6354624	0.0019559	0.9665460
H	-5.0031363	0.8152450	0.4980405
H	-5.0024963	-0.8105779	0.4833293
H	5.0022446	0.8109566	0.4837245
H	5.0032576	-0.8148406	0.4977566
N	0.0000459	0.0000033	-1.5874589
C	0.0000097	-1.1398062	-2.2671068
C	0.0000700	1.1398463	-2.2671096
C	-0.0000107	-1.1931436	-3.6533683

H	-0.0000009	-2.0456456	-1.6667437
C	0.0000664	1.1931622	-3.6534037
H	0.0000919	2.0456720	-1.6667812
C	0.0000238	-0.0000158	-4.3589084
H	-0.0000562	-2.1485062	-4.1644706
H	0.0000898	2.1484940	-4.1644991
H	0.0000165	-0.0000154	-5.4431589

5B:py

C	3.0474478	-1.2213838	0.7826846
H	3.6920405	-2.1001772	0.7859139
C	3.6971079	0.0003776	0.6836586
C	3.0456980	1.2209372	0.7862830
H	3.6893417	2.1003859	0.7926495
C	-3.0459259	1.2204704	0.7855448
H	-3.6897447	2.0998430	0.7906607
C	-3.6970333	-0.0002471	0.6835403
C	-3.0472055	-1.2218396	0.7841698
H	-3.6916087	-2.1007224	0.7887309
N	-5.1087016	0.0012325	0.6007200
N	5.1087838	0.0025151	0.6013004
H	-5.4730038	0.8252978	0.1463781
H	-5.4749405	-0.8194243	0.1419060
H	5.4729330	0.8230805	0.1404814
H	5.4756891	-0.8216632	0.1493696
S	1.4403208	1.6288809	0.9363555
S	1.4425074	-1.6324287	0.9328660
S	-1.4406615	1.6287211	0.9364734
S	-1.4422252	-1.6325310	0.9341915
Ni	0.0000197	-0.0024783	0.8750970
N	-0.0000757	0.0008508	-1.9137118
C	-0.0001508	-1.1378218	-2.5930420
C	0.0000155	1.1389417	-2.5940614
C	-0.0001974	-1.1929146	-3.9793976
H	-0.0001650	-2.0460403	-1.9950777

C	0.0000336	1.1927976	-3.9805406
H	0.0000877	2.0475167	-1.9965830
C	-0.0000995	-0.0004188	-4.6860178
H	-0.0003164	-2.1489706	-4.4893136
H	0.0001546	2.1483249	-4.4915004
H	-0.0001257	-0.0011121	-5.7703296

6A:py

C	2.5152927	-1.1991795	0.8380053
H	3.0756149	-2.1372409	0.8498626
C	3.2295133	-0.0001086	0.7256799
C	2.5153581	1.1989866	0.8380271
H	3.0757924	2.1369765	0.8500337
O	1.2664593	-1.3463649	0.9424602
O	1.2665242	1.3462499	0.9423906
C	-2.5154587	1.1991196	0.8378216
H	-3.0758137	2.1371818	0.8496331
C	-3.2296665	0.0000270	0.7255239
C	-2.5155337	-1.1990690	0.8379400
H	-3.0759435	-2.1370924	0.8500109
O	-1.2666324	1.3462841	0.9423517
O	-1.2666876	-1.3463156	0.9422747
Ni	-0.0000700	-0.0000410	0.9092916
N	-4.6373111	0.0000231	0.5202289
N	4.6371424	-0.0001373	0.5204168
C	-5.3279676	-1.2060322	0.9042580
H	-5.1618890	-1.4928961	1.9572456
H	-6.3979820	-1.0607571	0.7546222
H	-5.0308342	-2.0476286	0.2735798
C	-5.3279593	1.2059063	0.9047562
H	-5.0309398	2.0477278	0.2743391
H	-6.3979814	1.0606865	0.7552212
H	-5.1617187	1.4923971	1.9578140
C	5.3277967	1.2058338	0.9046848
H	5.1616185	1.4924975	1.9576996

H	6.3978306	1.0606071	0.7551038
H	5.0307040	2.0475116	0.2741280
C	5.3277527	-1.2061085	0.9047324
H	5.0306868	-2.0478204	0.2742036
H	6.3977752	-1.0609155	0.7552054
H	5.1615552	-1.4927662	1.9577480
N	0.0000623	0.0000253	-1.7561042
C	0.0001218	-1.1393615	-2.4364577
C	0.0000239	1.1393878	-2.4364837
C	0.0001777	-1.1929071	-3.8226870
H	0.0000855	-2.0459313	-1.8372444
C	0.0000454	1.1928861	-3.8227154
H	-0.0001400	2.0459722	-1.8372974
C	0.0001078	-0.0000159	-4.5284027
H	0.0002142	-2.1483461	-4.3336150
H	-0.0000305	2.1483128	-4.3336689
H	0.0000195	-0.0000201	-5.6126871

6B:py

C	-1.2154880	-3.0614210	-0.8187101
H	-2.1143032	-3.6664821	-0.8513192
C	0.0000061	-3.7418398	-0.7495804
C	1.2154926	-3.0614101	-0.8187195
H	2.1142904	-3.6664771	-0.8513277
C	1.2154966	3.0614119	-0.8187189
H	2.1142896	3.6664756	-0.8513275
C	-0.0000021	3.7418342	-0.7495809
C	-1.2154777	3.0614250	-0.8187104
H	-2.1143053	3.6664834	-0.8513195
N	-0.0000112	5.1457887	-0.6309987
N	-0.0000120	-5.1457852	-0.6309982
C	1.2175303	5.8465413	-0.9541790
H	1.5767950	5.6522889	-1.9777588
H	1.0441010	6.9172436	-0.8537535
H	2.0200315	5.5895067	-0.2577421

C	-1.2175063	5.8465507	-0.9541804
H	-2.0200406	5.5895031	-0.2577405
H	-1.0441041	6.9172347	-0.8537570
H	-1.5767945	5.6522873	-1.9777648
C	-1.2175073	-5.8465526	-0.9541807
H	-1.5767942	-5.6522869	-1.9777646
H	-1.0441037	-6.9172349	-0.8537568
H	-2.0200400	-5.5895027	-0.2577403
C	1.2175292	-5.8465414	-0.9541797
H	2.0200325	-5.5895069	-0.2577419
H	1.0441020	-6.9172428	-0.8537539
H	1.5767954	-5.6522891	-1.9777590
S	-1.6116591	1.4457470	-0.8798198
S	1.6116652	1.4457690	-0.8798274
S	-1.6116597	-1.4457503	-0.8798203
S	1.6116641	-1.4457684	-0.8798270
Ni	0.0000033	0.0000003	-0.8080211
N	-0.0000048	-0.0000000	2.0375199
C	-1.1378973	0.0000001	2.7184345
C	1.1378954	0.0000000	2.7184271
C	-1.1925416	0.0000000	4.1050402
H	-2.0470354	-0.0000001	2.1218888
C	1.1925532	0.0000000	4.1050366
H	2.0470248	0.0000002	2.1218885
C	-0.0000069	-0.0000000	4.8113919
H	-2.1483066	-0.0000000	4.6155656
H	2.1483016	0.0000000	4.6155582
H	0.0000020	-0.0000000	5.8958124

7A:py

C	2.5119912	-1.1957678	-0.6968037
H	3.0751500	-2.1338178	-0.7271995
C	3.2324884	0.0000252	-0.7196990
C	2.5120193	1.1957322	-0.6968033
H	3.0751385	2.1338192	-0.7271830

O	1.2639954	-1.3543474	-0.6448206
O	1.2639714	1.3543668	-0.6448187
C	-2.5119761	1.1957055	-0.6967935
H	-3.0751289	2.1338431	-0.7271834
C	-3.2324481	-0.0000227	-0.7196657
C	-2.5120279	-1.1956597	-0.6967859
H	-3.0751551	-2.1338542	-0.7272090
O	-1.2639863	1.3543632	-0.6447769
O	-1.2639823	-1.3543480	-0.6447465
Ni	0.0000060	0.0000178	-0.6031600
C	-4.7551174	0.0000107	-0.8682905
C	4.7551085	-0.0000019	-0.8682982
C	-5.3817948	1.2312784	-0.2070074
H	-5.1081540	2.1615571	-0.7084505
H	-6.4712926	1.1567656	-0.2504200
H	-5.0873960	1.3085160	0.8426043
C	-5.3817815	-1.2312849	-0.2070059
H	-6.4712991	-1.1567677	-0.2504173
H	-5.1081705	-2.1615664	-0.7084374
H	-5.0874094	-1.3085164	0.8426093
C	-5.1178692	-0.0000066	-2.3581200
H	-6.2029782	-0.0000001	-2.5013226
H	-4.7081788	0.8821971	-2.8564395
H	-4.7081848	-0.8821963	-2.8564463
C	5.1178659	-0.0000009	-2.3581207
H	4.7081823	0.8821989	-2.8564359
H	6.2029682	-0.0000006	-2.5013292
H	4.7081835	-0.8821940	-2.8564425
C	5.3817828	1.2312883	-0.2070107
H	6.4712924	1.1567669	-0.2504256
H	5.1081576	2.1615536	-0.7084543
H	5.0874023	1.3085218	0.8426038
C	5.3817895	-1.2312816	-0.2070086
H	5.0874033	-1.3085161	0.8426146
H	5.1081662	-2.1615601	-0.7084423

H	6.4712895	-1.1567661	-0.2504154
N	-0.0000009	-0.0000107	2.0418205
C	-0.0000024	-1.1397007	2.7220757
C	-0.0000011	1.1396937	2.7220871
C	-0.0000023	-1.1929761	4.1084019
H	-0.0000040	-2.0461741	2.1228433
C	-0.0000017	1.1929655	4.1083798
H	-0.0000025	2.0461521	2.1228289
C	-0.0000022	0.0000059	4.8140900
H	-0.0000024	-2.1483969	4.6193976
H	-0.0000014	2.1483983	4.6193819
H	-0.0000002	-0.0000074	5.8983723

7B:py

C	3.0239532	-1.2106691	-0.9401980
H	3.6476843	-2.0992551	-0.9071885
C	3.6626110	0.0000089	-0.7070685
C	3.0240404	1.2106902	-0.9402032
H	3.6478418	2.0992489	-0.9071831
C	-3.0239626	1.2107538	-0.9401160
H	-3.6477195	2.0993460	-0.9069884
C	-3.6625648	0.0000697	-0.7070756
C	-3.0239569	-1.2105839	-0.9403793
H	-3.6477185	-2.0991668	-0.9074826
C	-5.1586989	-0.0000098	-0.3477144
C	5.1587393	-0.0000374	-0.3477200
C	-5.5282639	1.2314939	0.4846813
H	-5.4543472	2.1612964	-0.0823866
H	-6.5651054	1.1489710	0.8193294
H	-4.8911436	1.3177101	1.3682256
C	-5.5280942	-1.2314852	0.4848154
H	-6.5649765	-1.1490698	0.8194263
H	-5.4540257	-2.1613377	-0.0821495
H	-4.8910126	-1.3174969	1.3683834
C	-5.9844698	-0.0001083	-1.6377748

H	-7.0550797	-0.0001587	-1.4132948
H	-5.7623349	0.8825247	-2.2426219
H	-5.7622552	-0.8827806	-2.2425486
C	5.9844895	-0.0000452	-1.6377996
H	5.7622850	0.8826079	-2.2425994
H	7.0551111	-0.0000479	-1.4133705
H	5.7622840	-0.8826969	-2.2426052
C	5.5281740	1.2314853	0.4847083
H	6.5650294	1.1491410	0.8193733
H	5.4541057	2.1612736	-0.0823562
H	4.8910209	1.3176069	1.3682349
C	5.5281440	-1.2315433	0.4847457
H	4.8910129	-1.3176151	1.3682869
H	5.4540548	-2.1613537	-0.0822815
H	6.5650106	-1.1491981	0.8193981
S	-1.4543360	1.6168231	-1.3107518
S	-1.4543071	-1.6165475	-1.3109851
S	1.4542889	-1.6166211	-1.3107215
S	1.4544127	1.6167858	-1.3107685
Ni	0.0000233	0.0001365	-1.2403766
N	-0.0000115	0.0000226	1.5084904
C	-0.0000414	1.1387307	2.1880526
C	0.0000359	-1.1387127	2.1879967
C	-0.0000358	1.1929124	3.5743619
H	-0.0000758	2.0466683	1.5898611
C	0.0000587	-1.1929524	3.5743084
H	0.0000559	-2.0466232	1.5897652
C	0.0000171	-0.0000471	4.2802538
H	-0.0000774	2.1486470	4.0847852
H	0.0001078	-2.1487238	4.0846827
H	0.0000226	-0.0000674	5.3645384

8A:py

C	2.5834611	-1.2309607	1.7570738
H	3.2157465	-2.1270420	1.7344072

C	3.2322230	0.0000000	1.7105393
H	4.3122273	0.0000000	1.6522808
C	2.5834611	1.2309607	1.7570738
H	3.2157465	2.1270420	1.7344072
O	1.3503996	-1.4650449	1.8269444
O	1.3503996	1.4650449	1.8269444
C	-2.5834611	1.2309607	1.7570738
H	-3.2157465	2.1270420	1.7344072
C	-3.2322230	0.0000000	1.7105393
H	-4.3122273	0.0000000	1.6522808
C	-2.5834611	-1.2309607	1.7570738
H	-3.2157465	-2.1270420	1.7344072
O	-1.3503996	1.4650449	1.8269444
O	-1.3503996	-1.4650449	1.8269444
Pd	0.0000000	0.0000000	1.8320181
N	-0.0000000	0.0000000	-1.1236612
C	0.0000000	-1.1383337	-1.8061688
C	0.0000000	1.1383337	-1.8061688
C	0.0000000	-1.1925376	-3.1926355
H	-0.0000000	-2.0477889	-1.2110898
C	-0.0000000	1.1925376	-3.1926355
H	-0.0000000	2.0477889	-1.2110898
C	-0.0000000	0.0000000	-3.8987644
H	0.0000000	-2.1482590	-3.7030805
H	0.0000000	2.1482590	-3.7030805
H	-0.0000000	0.0000000	-4.9829849

8B:py

C	-3.1080819	1.2445484	1.7942476
H	-3.7870502	2.0954668	1.7774745
C	-3.7148432	0.0000000	1.7538141
H	-4.7986749	0.0000000	1.7015533
C	-3.1080819	-1.2445484	1.7942476
H	-3.7870502	-2.0954668	1.7774745
C	3.1080819	-1.2445484	1.7942476

H	3.7870502	-2.0954668	1.7774745
C	3.7148432	0.0000000	1.7538141
H	4.7986749	0.0000000	1.7015533
C	3.1080819	1.2445484	1.7942476
H	3.7870502	2.0954668	1.7774745
S	-1.5208849	-1.7233649	1.8740330
S	-1.5208849	1.7233649	1.8740330
S	1.5208849	-1.7233649	1.8740330
S	1.5208849	1.7233649	1.8740330
Pd	0.0000000	0.0000000	1.8628171
N	0.0000000	0.0000000	-1.1895195
C	-0.0000000	1.1375611	-1.8718184
C	-0.0000000	-1.1375611	-1.8718184
C	-0.0000000	1.1925699	-3.2583407
H	0.0000000	2.0485296	-1.2782715
C	-0.0000000	-1.1925699	-3.2583407
H	0.0000000	-2.0485296	-1.2782715
C	0.0000000	0.0000000	-3.9645457
H	0.0000000	2.1485707	-3.7684546
H	0.0000000	-2.1485707	-3.7684546
H	0.0000000	0.0000000	-5.0487365

9A:py

C	2.5827108	-1.2391921	1.7542515
H	3.2429028	-2.1137158	1.7373951
C	3.2067330	0.0000000	1.7184341
C	2.5827108	1.2391921	1.7542515
H	3.2429028	2.1137158	1.7373951
O	1.3469312	-1.4685917	1.8072795
O	1.3469312	1.4685917	1.8072795
C	-2.5827108	1.2391921	1.7542515
H	-3.2429028	2.1137158	1.7373951
C	-3.2067330	0.0000000	1.7184341
C	-2.5827108	-1.2391921	1.7542515
H	-3.2429028	-2.1137158	1.7373951

O	-1.3469312	1.4685917	1.8072795
O	-1.3469312	-1.4685917	1.8072795
F	4.5601829	0.0000000	1.6604784
F	-4.5601829	0.0000000	1.6604784
Pd	0.0000000	0.0000000	1.8081643
N	0.0000000	0.0000000	-1.1171255
C	0.0000000	1.1386606	-1.7999514
C	-0.0000000	-1.1386606	-1.7999514
C	-0.0000000	1.1925711	-3.1861431
H	-0.0000000	2.0485147	-1.2055582
C	-0.0000000	-1.1925711	-3.1861431
H	-0.0000000	-2.0485147	-1.2055582
C	-0.0000000	0.0000000	-3.8921757
H	-0.0000000	2.1482976	-3.6963856
H	-0.0000000	-2.1482976	-3.6963856
H	-0.0000000	0.0000000	-4.9763157

9B:py

C	-3.1012564	1.2497393	1.7894338
H	-3.8112111	2.0743604	1.7750278
C	-3.6815075	0.0000000	1.7496564
C	-3.1012564	-1.2497393	1.7894338
H	-3.8112111	-2.0743604	1.7750278
C	3.1012564	-1.2497393	1.7894338
H	3.8112111	-2.0743604	1.7750278
C	3.6815075	0.0000000	1.7496564
C	3.1012564	1.2497393	1.7894338
H	3.8112111	2.0743604	1.7750278
F	-5.0358978	0.0000000	1.6841031
F	5.0358978	0.0000000	1.6841031
S	-1.5145509	-1.7283191	1.8648362
S	-1.5145509	1.7283191	1.8648362
S	1.5145509	-1.7283191	1.8648362
S	1.5145509	1.7283191	1.8648362
Pd	0.0000000	0.0000000	1.8507265

N	0.0000000	0.0000000	-1.1782312
C	0.0000000	1.1376152	-1.8608749
C	0.0000000	-1.1376152	-1.8608749
C	-0.0000000	1.1924696	-3.2471787
H	-0.0000000	2.0488632	-1.2677882
C	-0.0000000	-1.1924696	-3.2471787
H	-0.0000000	-2.0488632	-1.2677882
C	-0.0000000	0.0000000	-3.9534362
H	-0.0000000	2.1483457	-3.7572399
H	-0.0000000	-2.1483457	-3.7572399
H	0.0000000	0.0000000	-5.0376061

10A:py

C	-2.5838501	1.2427651	1.6292366
H	-3.2186261	2.1353673	1.6019691
C	-3.2407430	0.0000000	1.5664028
C	-2.5838501	-1.2427651	1.6292366
H	-3.2186261	-2.1353673	1.6019691
O	-1.3569310	1.4565026	1.7184744
O	-1.3569310	-1.4565026	1.7184744
C	2.5838501	-1.2427651	1.6292366
H	3.2186261	-2.1353673	1.6019691
C	3.2407430	0.0000000	1.5664028
C	2.5838501	1.2427651	1.6292366
H	3.2186261	2.1353673	1.6019691
O	1.3569310	-1.4565026	1.7184744
O	1.3569310	1.4565026	1.7184744
Pd	0.0000000	0.0000000	1.7203858
C	4.6578490	0.0000000	1.4551911
C	-4.6578490	0.0000000	1.4551911
N	5.8068787	0.0000000	1.3609370
N	-5.8068787	0.0000000	1.3609370
N	-0.0000000	0.0000000	-1.1636258
C	-0.0000000	-1.1390002	-1.8475196
C	-0.0000000	1.1390002	-1.8475196

C	0.0000000	-1.1925411	-3.2331776
H	-0.0000000	-2.0500983	-1.2551147
C	-0.0000000	1.1925411	-3.2331776
H	-0.0000000	2.0500983	-1.2551147
C	0.0000000	0.0000000	-3.9390745
H	0.0000000	-2.1480895	-3.7434133
H	0.0000000	2.1480895	-3.7434133
H	0.0000000	0.0000000	-5.0230176

10B:py

C	3.1016513	-1.2529468	1.6543657
H	3.7837278	-2.1008225	1.6321789
C	3.7170026	0.0000000	1.5923811
C	3.1016513	1.2529468	1.6543657
H	3.7837278	2.1008225	1.6321789
C	-3.1016513	1.2529468	1.6543657
H	-3.7837278	2.1008225	1.6321789
C	-3.7170026	0.0000000	1.5923811
C	-3.1016513	-1.2529468	1.6543657
H	-3.7837278	-2.1008225	1.6321789
S	1.5226604	1.7183774	1.7679672
S	1.5226604	-1.7183774	1.7679672
S	-1.5226604	-1.7183774	1.7679672
S	-1.5226604	1.7183774	1.7679672
Pd	0.0000000	0.0000000	1.7480992
C	-5.1422847	0.0000000	1.4817990
C	5.1422847	0.0000000	1.4817990
N	-6.2909825	0.0000000	1.3883968
N	6.2909825	0.0000000	1.3883968
N	0.0000000	0.0000000	-1.2190157
C	-0.0000000	1.1378955	-1.9023968
C	-0.0000000	-1.1378955	-1.9023968
C	0.0000000	1.1924583	-3.2882162
H	0.0000000	2.0502697	-1.3111325
C	0.0000000	-1.1924583	-3.2882162

H	0.0000000	-2.0502697	-1.3111325
C	0.0000000	0.0000000	-3.9943656
H	0.0000000	2.1483046	-3.7980337
H	0.0000000	-2.1483046	-3.7980337
H	0.0000000	0.0000000	-5.0783604

11A:py

C	2.5860409	-1.2465442	1.5376383
H	3.2305851	-2.1281295	1.5050272
C	3.2240361	0.0000000	1.4583665
C	2.5860409	1.2465442	1.5376383
H	3.2305851	2.1281295	1.5050272
O	1.3594302	-1.4537476	1.6478882
O	1.3594302	1.4537476	1.6478882
C	-2.5860409	1.2465442	1.5376383
H	-3.2305851	2.1281295	1.5050272
C	-3.2240361	-0.0000000	1.4583665
C	-2.5860409	-1.2465442	1.5376383
H	-3.2305851	-2.1281295	1.5050272
O	-1.3594302	1.4537476	1.6478882
O	-1.3594302	-1.4537476	1.6478882
N	-4.6636584	-0.0000000	1.3142286
N	4.6636584	0.0000000	1.3142286
O	-5.2294867	-1.0742737	1.2552473
O	-5.2294867	1.0742737	1.2552473
O	5.2294867	-1.0742737	1.2552473
O	5.2294867	1.0742737	1.2552473
Pd	0.0000000	0.0000000	1.6506351
N	0.0000000	0.0000000	-1.2268853
C	0.0000000	1.1391765	-1.9108217
C	0.0000000	-1.1391765	-1.9108217
C	0.0000000	1.1926674	-3.2963788
H	-0.0000000	2.0506279	-1.3189913
C	0.0000000	-1.1926674	-3.2963788
H	-0.0000000	-2.0506279	-1.3189913

C	0.0000000	0.0000000	-4.0022057
H	-0.0000000	2.1487130	-3.8057221
H	-0.0000000	-2.1487130	-3.8057221
H	0.0000000	0.0000000	-5.0861107

11B:py

C	3.1215000	-1.2530726	1.5814594
H	3.8403849	-2.0857546	1.5566929
C	3.7469717	-0.0000000	1.5295070
C	3.1215000	1.2530726	1.5814594
H	3.8403849	2.0857546	1.5566929
C	-3.1215000	1.2530726	1.5814594
H	-3.8403849	2.0857546	1.5566929
C	-3.7469717	0.0000000	1.5295070
C	-3.1215000	-1.2530726	1.5814594
H	-3.8403849	-2.0857546	1.5566929
N	-5.0965001	0.0000000	1.4539947
N	5.0965001	0.0000000	1.4539947
O	-5.7981758	-1.0708731	1.4180157
O	-5.7981758	1.0708731	1.4180157
O	5.7981758	-1.0708731	1.4180157
O	5.7981758	1.0708731	1.4180157
S	1.5502633	1.6821289	1.6864757
S	1.5502633	-1.6821289	1.6864757
S	-1.5502633	-1.6821289	1.6864757
S	-1.5502633	1.6821289	1.6864757
Pd	0.0000000	0.0000000	1.6470831
N	0.0000000	0.0000000	-1.3720896
C	0.0000000	1.1375364	-2.0563698
C	0.0000000	-1.1375364	-2.0563698
C	0.0000000	1.1923468	-3.4420052
H	-0.0000000	2.0506671	-1.4659705
C	0.0000000	-1.1923468	-3.4420052
H	-0.0000000	-2.0506671	-1.4659705
C	0.0000000	0.0000000	-4.1482589

H	-0.0000000	2.1482050	-3.9517004
H	-0.0000000	-2.1482050	-3.9517004
H	0.0000000	0.0000000	-5.2322211

12A:py

C	2.5948630	-1.2206631	1.6113400
H	3.2229406	-2.1223245	1.6040079
C	3.2657141	0.0000000	1.5634920
C	2.5948630	1.2206631	1.6113400
H	3.2229406	2.1223245	1.6040079
O	1.3559313	-1.4499809	1.6671744
O	1.3559313	1.4499809	1.6671744
C	-2.5948630	1.2206631	1.6113400
H	-3.2229406	2.1223245	1.6040079
C	-3.2657141	0.0000000	1.5634920
C	-2.5948630	-1.2206631	1.6113400
H	-3.2229406	-2.1223245	1.6040079
O	-1.3559313	1.4499809	1.6671744
O	-1.3559313	-1.4499809	1.6671744
N	-4.6957216	0.0000000	1.5781665
N	4.6957216	-0.0000000	1.5781665
H	-5.0648139	0.8121407	1.1022582
H	-5.0648139	-0.8121407	1.1022582
H	5.0648139	0.8121407	1.1022582
H	5.0648139	-0.8121407	1.1022582
Pd	0.0000000	0.0000000	1.6697803
N	0.0000000	0.0000000	-1.3111622
C	0.0000000	-1.1382843	-1.9934762
C	0.0000000	1.1382843	-1.9934762
C	0.0000000	-1.1926122	-3.3801290
H	0.0000000	-2.0474678	-1.3978724
C	0.0000000	1.1926122	-3.3801290
H	0.0000000	2.0474678	-1.3978724
C	-0.0000000	0.0000000	-4.0862856
H	0.0000000	-2.1483531	-3.8906487

H	-0.0000000	2.1483531	-3.8906487
H	0.0000000	0.0000000	-5.1705191

12B:py

C	-3.1165025	1.2326874	1.6393361
H	-3.7846644	2.0943804	1.6337927
C	-3.7531961	0.0000000	1.5688272
C	-3.1165025	-1.2326874	1.6393361
H	-3.7846644	-2.0943804	1.6337927
C	3.1165025	-1.2326874	1.6393361
H	3.7846644	-2.0943804	1.6337927
C	3.7531961	0.0000000	1.5688272
C	3.1165025	1.2326874	1.6393361
H	3.7846644	2.0943804	1.6337927
N	5.1691760	0.0000000	1.5242571
N	-5.1691760	-0.0000000	1.5242571
H	5.5456593	-0.8217038	1.0753348
H	5.5456593	0.8217038	1.0753348
H	-5.5456593	-0.8217038	1.0753348
H	-5.5456593	0.8217038	1.0753348
S	-1.5274388	-1.7063606	1.7523735
S	-1.5274388	1.7063606	1.7523735
S	1.5274388	-1.7063606	1.7523735
S	1.5274388	1.7063606	1.7523735
Pd	0.0000000	0.0000000	1.7377587
N	0.0000000	0.0000000	-1.3505323
C	0.0000000	1.1373642	-2.0327682
C	0.0000000	-1.1373642	-2.0327682
C	-0.0000000	1.1924564	-3.4195211
H	0.0000000	2.0478145	-1.4384043
C	-0.0000000	-1.1924564	-3.4195211
H	0.0000000	-2.0478145	-1.4384043
C	-0.0000000	0.0000000	-4.1260470
H	-0.0000000	2.1485569	-3.9294598
H	-0.0000000	-2.1485569	-3.9294598

H	-0.0000000	0.0000000	-5.2103891

13A:py

C	2.6096527	-1.2172639	1.0477602
H	3.2020150	-2.1345564	1.0411414
C	3.3021015	-0.0000000	0.9726219
C	2.6096527	1.2172639	1.0477602
H	3.2020150	2.1345564	1.0411414
O	1.3675771	-1.4283777	1.1262530
O	1.3675771	1.4283777	1.1262530
C	-2.6096527	1.2172639	1.0477602
H	-3.2020150	2.1345564	1.0411414
C	-3.3021015	0.0000000	0.9726219
C	-2.6096527	-1.2172639	1.0477602
H	-3.2020150	-2.1345564	1.0411414
O	-1.3675771	1.4283777	1.1262530
O	-1.3675771	-1.4283777	1.1262530
N	-4.7198733	0.0000000	0.8066760
N	4.7198733	0.0000000	0.8066760
C	-5.4029898	-1.2053099	1.2085307
H	-5.1899830	-1.5070443	2.2487304
H	-6.4771914	-1.0475344	1.1130262
H	-5.1485945	-2.0426450	0.5539030
C	-5.4029898	1.2053099	1.2085307
H	-5.1485945	2.0426450	0.5539030
H	-6.4771914	1.0475344	1.1130262
H	-5.1899830	1.5070443	2.2487304
C	5.4029898	1.2053099	1.2085307
H	5.1899830	1.5070443	2.2487304
H	6.4771914	1.0475344	1.1130262
H	5.1485945	2.0426450	0.5539030
C	5.4029898	-1.2053099	1.2085307
H	5.1485945	-2.0426450	0.5539030
H	6.4771914	-1.0475344	1.1130262
H	5.1899830	-1.5070443	2.2487304

Pd	0.0000000	0.0000000	1.1241412
N	0.0000000	0.0000000	-1.8697567
C	0.0000000	-1.1380014	-2.5523100
C	0.0000000	1.1380014	-2.5523100
C	-0.0000000	-1.1924491	-3.9389235
H	0.0000000	-2.0474335	-1.9571158
C	-0.0000000	1.1924491	-3.9389235
H	0.0000000	2.0474335	-1.9571158
C	0.0000000	0.0000000	-4.6451494
H	0.0000000	-2.1481098	-4.4495372
H	-0.0000000	2.1481098	-4.4495372
H	-0.0000000	0.0000000	-5.7294377

13B:py

C	3.1368931	-1.2264526	-1.0833065
H	3.7653975	-2.1096826	-1.0997510
C	3.8042806	0.0000000	-1.0492420
C	3.1368931	1.2264526	-1.0833065
H	3.7653975	2.1096826	-1.0997510
C	-3.1368931	1.2264526	-1.0833065
H	-3.7653975	2.1096826	-1.0997510
C	-3.8042806	0.0000000	-1.0492420
C	-3.1368931	-1.2264526	-1.0833065
H	-3.7653975	-2.1096826	-1.0997510
N	-5.2145432	0.0000000	-0.9752744
N	5.2145432	0.0000000	-0.9752744
C	-5.9073697	1.2174210	-1.3165219
H	-5.6644918	1.5918669	-2.3240227
H	-6.9801399	1.0343624	-1.2739308
H	-5.6953552	2.0131752	-0.5971350
C	-5.9073697	-1.2174210	-1.3165219
H	-5.6953552	-2.0131752	-0.5971350
H	-6.9801399	-1.0343624	-1.2739308
H	-5.6644918	-1.5918669	-2.3240227
C	5.9073697	-1.2174210	-1.3165219

H	5.6644918	-1.5918669	-2.3240227
H	6.9801399	-1.0343624	-1.2739308
H	5.6953552	-2.0131752	-0.5971350
C	5.9073697	1.2174210	-1.3165219
H	5.6953552	2.0131752	-0.5971350
H	6.9801399	1.0343624	-1.2739308
H	5.6644918	1.5918669	-2.3240227
S	-1.5382254	-1.6860702	-1.1038100
S	-1.5382254	1.6860702	-1.1038100
S	1.5382254	-1.6860702	-1.1038100
S	1.5382254	1.6860702	-1.1038100
Pd	0.0000000	0.0000000	-1.0756415
N	0.0000000	0.0000000	2.0758228
C	0.0000000	-1.1369671	2.7590446
C	0.0000000	1.1369671	2.7590446
C	0.0000000	-1.1921953	4.1459023
H	0.0000000	-2.0479012	2.1656097
C	0.0000000	1.1921953	4.1459023
H	-0.0000000	2.0479012	2.1656097
C	0.0000000	0.0000000	4.8525957
H	0.0000000	-2.1482224	4.6560411
H	0.0000000	2.1482224	4.6560411
H	-0.0000000	0.0000000	5.9369719

14A:py

C	2.5995009	-1.2136807	-1.0581415
H	3.2001435	-2.1279092	-1.0418291
C	3.2864879	0.0000000	-0.9589661
C	2.5995009	1.2136807	-1.0581415
H	3.2001435	2.1279092	-1.0418291
O	1.3653317	-1.4367762	-1.1744891
O	1.3653317	1.4367762	-1.1744891
C	-2.5995009	1.2136807	-1.0581415
H	-3.2001435	2.1279092	-1.0418291
C	-3.2864879	0.0000000	-0.9589661

C	-2.5995009	-1.2136807	-1.0581415
H	-3.2001435	-2.1279092	-1.0418291
O	-1.3653317	1.4367762	-1.1744891
O	-1.3653317	-1.4367762	-1.1744891
C	-4.8177294	0.0000000	-0.8540236
C	4.8177294	0.0000000	-0.8540236
C	-5.3262533	1.2298722	-0.0940369
H	-5.1599752	2.1609537	-0.6391714
H	-6.4046379	1.1442140	0.0603901
H	-4.8481801	1.3155171	0.8850617
C	-5.3262533	-1.2298722	-0.0940369
H	-6.4046379	-1.1442140	0.0603901
H	-5.1599752	-2.1609537	-0.6391714
H	-4.8481801	-1.3155171	0.8850617
C	-5.4295710	0.0000000	-2.2591028
H	-6.5228387	0.0000000	-2.2119834
H	-5.1126313	0.8824166	-2.8209740
H	-5.1126313	-0.8824166	-2.8209740
C	5.4295710	0.0000000	-2.2591028
H	5.1126313	0.8824166	-2.8209740
H	6.5228387	0.0000000	-2.2119834
H	5.1126313	-0.8824166	-2.8209740
C	5.3262533	1.2298722	-0.0940369
H	6.4046379	1.1442140	0.0603901
H	5.1599752	2.1609537	-0.6391714
H	4.8481801	1.3155171	0.8850617
C	5.3262533	-1.2298722	-0.0940369
H	4.8481801	-1.3155171	0.8850617
H	5.1599752	-2.1609537	-0.6391714
H	6.4046379	-1.1442140	0.0603901
Pd	0.0000000	0.0000000	-1.1788520
N	0.0000000	0.0000000	1.8009568
C	-0.0000000	-1.1382204	2.4832094
C	-0.0000000	1.1382204	2.4832094
C	0.0000000	-1.1926233	3.8698547

H	0.0000000	-2.0475657	1.8878656
C	0.0000000	1.1926233	3.8698547
H	0.0000000	2.0475657	1.8878656
C	0.0000000	0.0000000	4.5759944
H	-0.0000000	-2.1483883	4.3803596
H	-0.0000000	2.1483883	4.3803596
H	0.0000000	0.0000000	5.6602351

14B:py

C	3.1306041	-1.2231565	-0.8806337
H	3.7673433	-2.1024523	-0.9068028
C	3.7912657	-0.0000597	-0.9092492
C	3.1306091	1.2229574	-0.8807266
H	3.7675507	2.1020734	-0.9071198
C	-3.1305563	1.2229821	-0.8807522
H	-3.7675013	2.1020663	-0.9071666
C	-3.7912389	-0.0000866	-0.9092747
C	-3.1305498	-1.2231617	-0.8806585
H	-3.7673024	-2.1024550	-0.9068238
C	-5.3249660	0.0000549	-1.0746624
C	5.3250187	0.0000554	-1.0746373
C	-5.9697554	1.2306364	-0.4297729
H	-5.7161944	2.1592687	-0.9447717
H	-7.0571119	1.1364784	-0.4757813
H	-5.6814882	1.3288922	0.6196834
C	-5.9697479	-1.2305621	-0.4299107
H	-7.0571118	-1.1364838	-0.4758800
H	-5.7160552	-2.1590735	-0.9450361
H	-5.6814175	-1.3288789	0.6195306
C	-5.6442821	0.0000536	-2.5738727
H	-6.7254572	0.0000646	-2.7413898
H	-5.2226995	0.8826144	-3.0611780
H	-5.2227038	-0.8825258	-3.0611088
C	5.6443357	0.0000534	-2.5738444
H	5.2227594	0.8826133	-3.0611565

H	6.7255086	0.0000592	-2.7413555
H	5.2227573	-0.8825254	-3.0610838
C	5.9697955	1.2306368	-0.4297505
H	7.0571533	1.1364795	-0.4757500
H	5.7162461	2.1592700	-0.9447476
H	5.6815304	1.3289003	0.6197038
C	5.9697854	-1.2305556	-0.4298775
H	5.6814369	-1.3288720	0.6195606
H	5.7160927	-2.1590687	-0.9450030
H	7.0571443	-1.1364855	-0.4758313
S	-1.5377525	1.6932206	-0.8192582
S	-1.5377200	-1.6932152	-0.8190317
S	1.5377739	-1.6932177	-0.8190181
S	1.5377895	1.6932198	-0.8192065
Pd	0.0000209	0.0000295	-0.7961568
N	-0.0003092	-0.0000302	2.3188052
C	-0.0001851	1.1372209	3.0018610
C	-0.0001950	-1.1372110	3.0018980
C	0.0000346	1.1923899	4.3886163
H	-0.0003460	2.0484556	2.4088579
C	0.0000287	-1.1923221	4.3886784
H	-0.0003667	-2.0484957	2.4089320
C	0.0001006	0.0000265	5.0951502
H	0.0001071	2.1484526	4.8987255
H	0.0000980	-2.1483872	4.8988085
H	0.0001236	0.0000564	6.1794695

15A:py

C	2.5787321	-1.2332432	1.8687560
H	3.2058990	-2.1303564	1.8493009
C	3.2218082	-0.0000000	1.8425755
H	4.3027970	0.0000000	1.8045534
C	2.5787321	1.2332432	1.8687560
H	3.2058990	2.1303564	1.8493009
O	1.3401633	-1.4701796	1.9141864

O	1.3401633	1.4701796	1.9141864
C	-2.5787321	1.2332432	1.8687560
H	-3.2058990	2.1303564	1.8493009
C	-3.2218082	0.0000000	1.8425755
H	-4.3027970	0.0000000	1.8045534
C	-2.5787321	-1.2332432	1.8687560
H	-3.2058990	-2.1303564	1.8493009
O	-1.3401633	1.4701796	1.9141864
O	-1.3401633	-1.4701796	1.9141864
Pt	0.0000000	0.0000000	1.9266499
N	0.0000000	0.0000000	-1.2962157
C	-0.0000000	-1.1371391	-1.9801605
C	-0.0000000	1.1371391	-1.9801605
C	0.0000000	-1.1922885	-3.3669093
H	0.0000000	-2.0481981	-1.3874165
C	0.0000000	1.1922885	-3.3669093
H	0.0000000	2.0481981	-1.3874165
C	-0.0000000	0.0000000	-4.0734446
H	0.0000000	-2.1484564	-3.8768114
H	0.0000000	2.1484564	-3.8768114
H	0.0000000	0.0000000	-5.1576248

15B:py

C	3.0939948	-1.2456094	1.8945460
H	3.7700297	-2.0982729	1.8841813
C	3.6984557	0.0000000	1.8783310
H	4.7832736	0.0000000	1.8549308
C	3.0939948	1.2456094	1.8945460
H	3.7700297	2.0982729	1.8841813
C	-3.0939948	1.2456094	1.8945460
H	-3.7700297	2.0982729	1.8841813
C	-3.6984557	0.0000000	1.8783310
H	-4.7832736	0.0000000	1.8549308
C	-3.0939948	-1.2456094	1.8945460
H	-3.7700297	-2.0982729	1.8841813

Pt	0.0000000	0.0000000	1.9353188
S	1.5066634	1.7284602	1.9308765
S	1.5066634	-1.7284602	1.9308765
S	-1.5066634	1.7284602	1.9308765
S	-1.5066634	-1.7284602	1.9308765
N	0.0000000	0.0000000	-1.3409039
C	-0.0000000	1.1367656	-2.0245843
C	-0.0000000	-1.1367656	-2.0245843
C	0.0000000	1.1921884	-3.4113201
H	-0.0000000	2.0487267	-1.4325746
C	0.0000000	-1.1921884	-3.4113201
H	-0.0000000	-2.0487267	-1.4325746
C	0.0000000	0.0000000	-4.1180492
H	0.0000000	2.1484471	-3.9209980
H	0.0000000	-2.1484471	-3.9209980
H	0.0000000	0.0000000	-5.2023507

16A:py

C	2.5769300	-1.2410231	1.8565296
H	3.2313352	-2.1172406	1.8386451
C	3.1961528	0.0000000	1.8321254
C	2.5769300	1.2410231	1.8565296
H	3.2313352	2.1172406	1.8386451
O	1.3363408	-1.4728218	1.8988574
O	1.3363408	1.4728218	1.8988574
C	-2.5769300	1.2410231	1.8565296
H	-3.2313352	2.1172406	1.8386451
C	-3.1961528	-0.0000000	1.8321254
C	-2.5769300	-1.2410231	1.8565296
H	-3.2313352	-2.1172406	1.8386451
O	-1.3363408	1.4728218	1.8988574
O	-1.3363408	-1.4728218	1.8988574
F	4.5494330	0.0000000	1.7856929
F	-4.5494330	-0.0000000	1.7856929
Pt	0.0000000	0.0000000	1.9103573

N	0.0000000	0.0000000	-1.2754248
C	-0.0000000	-1.1374561	-1.9595268
C	-0.0000000	1.1374561	-1.9595268
C	0.0000000	-1.1922815	-3.3460643
H	0.0000000	-2.0487779	-1.3671019
C	0.0000000	1.1922815	-3.3460643
H	0.0000000	2.0487779	-1.3671019
C	0.0000000	0.0000000	-4.0525667
H	0.0000000	-2.1482846	-3.8559998
H	0.0000000	2.1482846	-3.8559998
H	0.0000000	0.0000000	-5.1367455

16B:py

C	3.0872773	-1.2504482	1.8817215
H	3.7938107	-2.0772490	1.8695075
C	3.6656567	0.0000000	1.8602183
C	3.0872773	1.2504482	1.8817215
H	3.7938107	2.0772490	1.8695075
C	-3.0872773	1.2504482	1.8817215
H	-3.7938107	2.0772490	1.8695075
C	-3.6656567	0.0000000	1.8602183
C	-3.0872773	-1.2504482	1.8817215
H	-3.7938107	-2.0772490	1.8695075
F	5.0210394	-0.0000000	1.8208537
F	-5.0210394	0.0000000	1.8208537
Pt	0.0000000	0.0000000	1.9311953
S	1.5004400	1.7324931	1.9275011
S	1.5004400	-1.7324931	1.9275011
S	-1.5004400	1.7324931	1.9275011
S	-1.5004400	-1.7324931	1.9275011
N	0.0000000	0.0000000	-1.3197047
C	0.0000000	-1.1369403	-2.0035460
C	0.0000000	1.1369403	-2.0035460
C	0.0000000	-1.1922539	-3.3900694
H	-0.0000000	-2.0492052	-1.4120060

C	0.0000000	1.1922539	-3.3900694
H	-0.0000000	2.0492052	-1.4120060
C	0.0000000	0.0000000	-4.0966097
H	0.0000000	-2.1483002	-3.8999588
H	0.0000000	2.1483002	-3.8999588
H	0.0000000	0.0000000	-5.1807845

17A:py

C	-2.5802706	1.2445498	1.7190321
H	-3.2095963	2.1387229	1.7002073
C	-3.2331702	0.0000000	1.6865065
C	-2.5802706	-1.2445498	1.7190321
H	-3.2095963	-2.1387229	1.7002073
O	-1.3467342	1.4609519	1.7703962
O	-1.3467342	-1.4609519	1.7703962
C	2.5802706	-1.2445498	1.7190321
H	3.2095963	-2.1387229	1.7002073
C	3.2331702	0.0000000	1.6865065
C	2.5802706	1.2445498	1.7190321
H	3.2095963	2.1387229	1.7002073
O	1.3467342	-1.4609519	1.7703962
O	1.3467342	1.4609519	1.7703962
Pt	0.0000000	0.0000000	1.7809855
C	4.6541137	-0.0000000	1.6265202
C	-4.6541137	0.0000000	1.6265202
N	5.8057860	0.0000000	1.5781557
N	-5.8057860	-0.0000000	1.5781557
N	0.0000000	0.0000000	-1.3474751
C	-0.0000000	1.1377601	-2.0323312
C	0.0000000	-1.1377601	-2.0323312
C	0.0000000	1.1922504	-3.4183777
H	0.0000000	2.0502242	-1.4417533
C	0.0000000	-1.1922504	-3.4183777
H	0.0000000	-2.0502242	-1.4417533
C	0.0000000	0.0000000	-4.1248089

H	0.0000000	2.1482842	-3.9279255
H	0.0000000	-2.1482842	-3.9279255
H	-0.0000000	0.0000000	-5.2088336

17B:py

C	3.0883639	-1.2538399	1.7407821
H	3.7672391	-2.1036887	1.7273165
C	3.7028667	0.0000000	1.7121430
C	3.0883639	1.2538399	1.7407821
H	3.7672391	2.1036887	1.7273165
C	-3.0883639	1.2538399	1.7407821
H	-3.7672391	2.1036887	1.7273165
C	-3.7028667	0.0000000	1.7121430
C	-3.0883639	-1.2538399	1.7407821
H	-3.7672391	-2.1036887	1.7273165
Pt	0.0000000	0.0000000	1.7985159
S	1.5075279	1.7235492	1.7978542
S	1.5075279	-1.7235492	1.7978542
S	-1.5075279	-1.7235492	1.7978542
S	-1.5075279	1.7235492	1.7978542
C	-5.1315833	-0.0000000	1.6597145
C	5.1315833	0.0000000	1.6597145
N	-6.2832454	0.0000000	1.6173137
N	6.2832454	0.0000000	1.6173137
N	0.0000000	0.0000000	-1.3947742
C	0.0000000	-1.1371468	-2.0792203
C	0.0000000	1.1371468	-2.0792203
C	0.0000000	-1.1922290	-3.4653459
H	0.0000000	-2.0505107	-1.4894212
C	0.0000000	1.1922290	-3.4653459
H	0.0000000	2.0505107	-1.4894212
C	0.0000000	0.0000000	-4.1718234
H	0.0000000	-2.1481569	-3.9751301
H	0.0000000	2.1481569	-3.9751301
H	0.0000000	0.0000000	-5.2558368

18A:py

C	2.5845552	-1.2481860	1.6148645
H	3.2240537	-2.1317114	1.6135729
C	3.2216213	0.0000000	1.6193279
C	2.5845552	1.2481860	1.6148645
H	3.2240537	2.1317114	1.6135729
O	1.3485795	-1.4577704	1.6124271
O	1.3485795	1.4577704	1.6124271
C	-2.5845552	1.2481860	1.6148645
H	-3.2240537	2.1317114	1.6135729
C	-3.2216213	0.0000000	1.6193279
C	-2.5845552	-1.2481860	1.6148645
H	-3.2240537	-2.1317114	1.6135729
O	-1.3485795	1.4577704	1.6124271
O	-1.3485795	-1.4577704	1.6124271
N	-4.6706288	0.0000000	1.6307112
N	4.6706288	0.0000000	1.6307112
O	-5.2380691	-1.0744246	1.6366284
O	-5.2380691	1.0744246	1.6366284
O	5.2380691	-1.0744246	1.6366284
O	5.2380691	1.0744246	1.6366284
Pt	0.0000000	0.0000000	1.6149313
N	0.0000000	0.0000000	-1.5026878
C	0.0000000	1.1382510	-2.1870228
C	0.0000000	-1.1382510	-2.1870228
C	0.0000000	1.1925673	-3.5731576
H	-0.0000000	2.0502915	-1.5957870
C	0.0000000	-1.1925673	-3.5731576
H	-0.0000000	-2.0502915	-1.5957870
C	0.0000000	0.0000000	-4.2790415
H	0.0000000	2.1478978	-4.0841641
H	0.0000000	-2.1478978	-4.0841641
H	0.0000000	0.0000000	-5.3629891

18B:py

C	3.0803077	-1.2613526	1.6572716
H	3.7914375	-2.0958361	1.6570552
C	3.6975775	0.0000000	1.5879670
C	3.0803077	1.2613526	1.6572716
H	3.7914375	2.0958361	1.6570552
C	-3.0803077	1.2613526	1.6572716
H	-3.7914375	2.0958361	1.6570552
C	-3.6975775	0.0000000	1.5879670
C	-3.0803077	-1.2613526	1.6572716
H	-3.7914375	-2.0958361	1.6570552
N	-5.0370988	0.0000000	1.4680251
N	5.0370988	0.0000000	1.4680251
O	-5.7650429	-1.0569604	1.4106662
O	-5.7650429	1.0569604	1.4106662
O	5.7650429	-1.0569604	1.4106662
O	5.7650429	1.0569604	1.4106662
Pt	0.0000000	0.0000000	1.7935393
S	1.5095929	1.7028466	1.7382761
S	1.5095929	-1.7028466	1.7382761
S	-1.5095929	-1.7028466	1.7382761
S	-1.5095929	1.7028466	1.7382761
N	0.0000000	0.0000000	-1.4774380
C	-0.0000000	1.1367913	-2.1627629
C	-0.0000000	-1.1367913	-2.1627629
C	-0.0000000	1.1921449	-3.5487376
H	0.0000000	2.0507083	-1.5735067
C	-0.0000000	-1.1921449	-3.5487376
H	0.0000000	-2.0507083	-1.5735067
C	0.0000000	0.0000000	-4.2552709
H	0.0000000	2.1481510	-4.0583039
H	0.0000000	-2.1481510	-4.0583039
H	-0.0000000	0.0000000	-5.3392690

19A:py

C	2.5898623	-1.2231084	1.7009932

H	3.2125368	-2.1256912	1.6892050
C	3.2551009	0.0000000	1.6563776
C	2.5898623	1.2231084	1.7009932
H	3.2125368	2.1256912	1.6892050
O	1.3467481	-1.4558018	1.7576883
O	1.3467481	1.4558018	1.7576883
C	-2.5898623	1.2231084	1.7009932
H	-3.2125368	2.1256912	1.6892050
C	-3.2551009	0.0000000	1.6563776
C	-2.5898623	-1.2231084	1.7009932
H	-3.2125368	-2.1256912	1.6892050
O	-1.3467481	1.4558018	1.7576883
O	-1.3467481	-1.4558018	1.7576883
N	-4.6843522	0.0000000	1.6673205
N	4.6843522	0.0000000	1.6673205
H	-5.0535722	0.8124865	1.1923221
H	-5.0535722	-0.8124865	1.1923221
H	5.0535722	0.8124865	1.1923221
H	5.0535722	-0.8124865	1.1923221
Pt	0.0000000	0.0000000	1.7701893
N	0.0000000	0.0000000	-1.4805754
C	0.0000000	-1.1371228	-2.1644736
C	0.0000000	1.1371228	-2.1644736
C	0.0000000	-1.1922672	-3.5514366
H	-0.0000000	-2.0478483	-1.5710276
C	0.0000000	1.1922672	-3.5514366
H	-0.0000000	2.0478483	-1.5710276
C	0.0000000	0.0000000	-4.2581115
H	-0.0000000	-2.1482160	-4.0617374
H	-0.0000000	2.1482160	-4.0617374
H	-0.0000000	0.0000000	-5.3423825

19B:py

C	3.1024598	-1.2341016	1.7135543
H	3.7675010	-2.0974767	1.7114779

C	3.7383396	0.0000000	1.6725062
C	3.1024598	1.2341016	1.7135543
H	3.7675010	2.0974767	1.7114779
C	-3.1024598	1.2341016	1.7135543
H	-3.7675010	2.0974767	1.7114779
C	-3.7383396	0.0000000	1.6725062
C	-3.1024598	-1.2341016	1.7135543
H	-3.7675010	-2.0974767	1.7114779
N	-5.1546828	0.0000000	1.6748902
N	5.1546828	0.0000000	1.6748902
H	-5.5469107	0.8220006	1.2402375
H	-5.5469107	-0.8220006	1.2402375
H	5.5469107	0.8220006	1.2402375
H	5.5469107	-0.8220006	1.2402375
Pt	0.0000000	0.0000000	1.7829132
S	1.5127619	1.7137549	1.7811514
S	1.5127619	-1.7137549	1.7811514
S	-1.5127619	1.7137549	1.7811514
S	-1.5127619	-1.7137549	1.7811514
N	0.0000000	0.0000000	-1.5250743
C	0.0000000	1.1367870	-2.2084756
C	0.0000000	-1.1367870	-2.2084756
C	0.0000000	1.1922633	-3.5954110
H	-0.0000000	2.0481193	-1.6154582
C	0.0000000	-1.1922633	-3.5954110
H	-0.0000000	-2.0481193	-1.6154582
C	0.0000000	0.0000000	-4.3020938
H	-0.0000000	2.1482982	-4.1055669
H	-0.0000000	-2.1482982	-4.1055669
H	0.0000000	0.0000000	-5.3863988

20A:py

C	2.6066615	-1.2196075	1.1221292
H	3.1945214	-2.1374598	1.1168304
C	3.2937829	0.0000000	1.0482543

C	2.6066615	1.2196075	1.1221292
H	3.1945214	2.1374598	1.1168304
O	1.3602399	-1.4349126	1.1972194
O	1.3602399	1.4349126	1.1972194
C	-2.6066615	1.2196075	1.1221292
H	-3.1945214	2.1374598	1.1168304
C	-3.2937829	0.0000000	1.0482543
C	-2.6066615	-1.2196075	1.1221292
H	-3.1945214	-2.1374598	1.1168304
O	-1.3602399	1.4349126	1.1972194
O	-1.3602399	-1.4349126	1.1972194
N	-4.7098066	0.0000000	0.8760091
N	4.7098066	-0.0000000	0.8760091
C	-5.3984762	-1.2062904	1.2646149
H	-5.2023597	-1.5106895	2.3072528
H	-6.4709825	-1.0483140	1.1521383
H	-5.1341706	-2.0417074	0.6114540
C	-5.3984762	1.2062904	1.2646149
H	-5.1341706	2.0417074	0.6114540
H	-6.4709825	1.0483140	1.1521383
H	-5.2023597	1.5106895	2.3072528
C	5.3984762	1.2062904	1.2646149
H	5.2023597	1.5106895	2.3072528
H	6.4709825	1.0483140	1.1521383
H	5.1341706	2.0417074	0.6114540
C	5.3984762	-1.2062904	1.2646149
H	5.1341706	-2.0417074	0.6114540
H	6.4709825	-1.0483140	1.1521383
H	5.2023597	-1.5106895	2.3072528
Pt	0.0000000	0.0000000	1.2029493
N	-0.0000000	0.0000000	-2.0584701
C	-0.0000000	-1.1368706	-2.7425790
C	-0.0000000	1.1368706	-2.7425790
C	0.0000000	-1.1921537	-4.1295065
H	-0.0000000	-2.0477685	-2.1495103

C	0.0000000	1.1921537	-4.1295065
H	-0.0000000	2.0477685	-2.1495103
C	-0.0000000	0.0000000	-4.8362224
H	-0.0000000	-2.1481341	-4.6398097
H	0.0000000	2.1481341	-4.6398097
H	0.0000000	0.0000000	-5.9205287

20B:py

C	3.1200558	-1.2282061	1.0762432
H	3.7478787	-2.1115129	1.0817487
C	3.7840786	0.0000000	1.0890229
C	3.1200558	1.2282061	1.0762432
H	3.7478787	2.1115129	1.0817487
C	-3.1200558	1.2282061	1.0762432
H	-3.7478787	2.1115129	1.0817487
C	-3.7840786	-0.0000000	1.0890229
C	-3.1200558	-1.2282061	1.0762432
H	-3.7478787	-2.1115129	1.0817487
N	-5.1968068	0.0000000	1.0907901
N	5.1968068	-0.0000000	1.0907901
C	-5.8664376	-1.2176618	1.4769124
H	-5.5483060	-1.5966784	2.4612059
H	-6.9387176	-1.0316995	1.5161268
H	-5.7123004	-2.0112778	0.7406910
C	-5.8664376	1.2176618	1.4769124
H	-5.7123004	2.0112778	0.7406910
H	-6.9387176	1.0316995	1.5161268
H	-5.5483060	1.5966784	2.4612059
C	5.8664376	1.2176618	1.4769124
H	5.5483060	1.5966784	2.4612059
H	6.9387176	1.0316995	1.5161268
H	5.7123004	2.0112778	0.7406910
C	5.8664376	-1.2176618	1.4769124
H	5.7123004	-2.0112778	0.7406910
H	6.9387176	-1.0316995	1.5161268

H	5.5483060	-1.5966784	2.4612059
Pt	0.0000000	0.0000000	1.0404672
S	-1.5242492	-1.6958120	1.0410665
S	-1.5242492	1.6958120	1.0410665
S	1.5242492	-1.6958120	1.0410665
S	1.5242492	1.6958120	1.0410665
N	0.0000000	0.0000000	-2.3162553
C	0.0000000	-1.1364877	-3.0003494
C	0.0000000	1.1364877	-3.0003494
C	0.0000000	-1.1921032	-4.3874251
H	0.0000000	-2.0482255	-2.4079676
C	0.0000000	1.1921032	-4.3874251
H	0.0000000	2.0482255	-2.4079676
C	0.0000000	0.0000000	-5.0943611
H	-0.0000000	-2.1482569	-4.8975925
H	-0.0000000	2.1482569	-4.8975925
H	-0.0000000	0.0000000	-6.1787860

21A:py

C	2.6007128	-1.2162738	-0.9404405
H	3.1947294	-2.1321800	-0.9541943
C	3.2882574	-0.0000000	-0.9539809
C	2.6007128	1.2162738	-0.9404405
H	3.1947294	2.1321800	-0.9541943
O	1.3570332	-1.4428089	-0.9137475
O	1.3570332	1.4428089	-0.9137475
C	-2.6007128	1.2162738	-0.9404405
H	-3.1947294	2.1321800	-0.9541943
C	-3.2882574	-0.0000000	-0.9539809
C	-2.6007128	-1.2162738	-0.9404405
H	-3.1947294	-2.1321800	-0.9541943
O	-1.3570332	1.4428089	-0.9137475
O	-1.3570332	-1.4428089	-0.9137475
C	-4.8189297	0.0000000	-1.0813028
C	4.8189297	0.0000000	-1.0813028

C	-5.4429297	1.2303921	-0.4146861
H	-5.1897889	2.1608006	-0.9265443
H	-6.5319862	1.1449851	-0.4405068
H	-5.1332132	1.3169207	0.6297717
C	-5.4429297	-1.2303921	-0.4146861
H	-6.5319862	-1.1449851	-0.4405068
H	-5.1897889	-2.1608006	-0.9265443
H	-5.1332132	-1.3169207	0.6297717
C	-5.1984683	0.0000000	-2.5666763
H	-6.2854282	0.0000000	-2.6953245
H	-4.7953315	0.8823729	-3.0699197
H	-4.7953315	-0.8823729	-3.0699197
C	5.1984683	0.0000000	-2.5666763
H	4.7953315	0.8823729	-3.0699197
H	6.2854282	0.0000000	-2.6953245
H	4.7953315	-0.8823729	-3.0699197
C	5.4429297	1.2303921	-0.4146861
H	6.5319862	1.1449851	-0.4405068
H	5.1897889	2.1608006	-0.9265443
H	5.1332132	1.3169207	0.6297717
C	5.4429297	-1.2303921	-0.4146861
H	5.1332132	-1.3169207	0.6297717
H	5.1897889	-2.1608006	-0.9265443
H	6.5319862	-1.1449851	-0.4405068
Pt	-0.0000000	0.0000000	-0.9119588
N	0.0000000	0.0000000	2.3748625
C	0.0000000	-1.1367868	3.0596034
C	0.0000000	1.1367868	3.0596034
C	0.0000000	-1.1920069	4.4466999
H	0.0000000	-2.0481788	2.4674204
C	0.0000000	1.1920069	4.4466999
H	0.0000000	2.0481788	2.4674204
C	0.0000000	0.0000000	5.1538257
H	0.0000000	-2.1482749	4.9566104
H	0.0000000	2.1482749	4.9566104

H	0.0000000	0.0000000	6.2382411

21B:py

C	-3.1148868	1.2244204	-0.9550725
H	-3.7504802	2.1041851	-0.9689803
C	-3.7726109	0.0000000	-0.9627605
C	-3.1148868	-1.2244204	-0.9550725
H	-3.7504802	-2.1041851	-0.9689803
C	3.1148868	-1.2244204	-0.9550725
H	3.7504802	-2.1041851	-0.9689803
C	3.7726109	0.0000000	-0.9627605
C	3.1148868	1.2244204	-0.9550725
H	3.7504802	2.1041851	-0.9689803
C	5.3110108	0.0000000	-1.0801642
C	-5.3110108	0.0000000	-1.0801642
C	5.9347323	-1.2305638	-0.4149068
H	5.6968824	-2.1593870	-0.9370758
H	7.0229416	-1.1371289	-0.4272093
H	5.6140615	-1.3276984	0.6252210
C	5.9347323	1.2305638	-0.4149068
H	7.0229416	1.1371289	-0.4272093
H	5.6968824	2.1593870	-0.9370758
H	5.6140615	1.3276984	0.6252210
C	5.6779388	0.0000000	-2.5681364
H	6.7639793	0.0000000	-2.7009112
H	5.2723226	-0.8826415	-3.0685154
H	5.2723226	0.8826415	-3.0685154
C	-5.6779388	0.0000000	-2.5681364
H	-5.2723226	-0.8826415	-3.0685154
H	-6.7639793	0.0000000	-2.7009112
H	-5.2723226	0.8826415	-3.0685154
C	-5.9347323	-1.2305638	-0.4149068
H	-7.0229416	-1.1371289	-0.4272093
H	-5.6968824	-2.1593870	-0.9370758
H	-5.6140615	-1.3276984	0.6252210

C	-5.9347323	1.2305638	-0.4149068
H	-5.6140615	1.3276984	0.6252210
H	-5.6968824	2.1593870	-0.9370758
H	-7.0229416	1.1371289	-0.4272093
Pt	0.0000000	0.0000000	-0.9299928
S	1.5234625	-1.7016942	-0.9344116
S	1.5234625	1.7016942	-0.9344116
S	-1.5234625	1.7016942	-0.9344116
S	-1.5234625	-1.7016942	-0.9344116
N	0.0000000	0.0000000	2.3982517
C	0.0000000	1.1366163	3.0823573
C	0.0000000	-1.1366163	3.0823573
C	0.0000000	1.1920968	4.4693159
H	0.0000000	2.0484865	2.4903023
C	0.0000000	-1.1920968	4.4693159
H	0.0000000	-2.0484865	2.4903023
C	0.0000000	0.0000000	5.1761713
H	-0.0000000	2.1481757	4.9794153
H	-0.0000000	-2.1481757	4.9794153
H	0.0000000	0.0000000	6.2605357

Cartesian coordinates of the optimized pentafluoroiodobenzene dimers

1A:C_6F5_I

C	1.2062052	2.4956899	3.2513326
H	2.1357008	3.0753694	3.2299275
C	-0.0000023	3.1816661	3.2036639
H	0.0000028	4.2602936	3.1419921
C	-1.2062062	2.4957013	3.2513294
H	-2.1357058	3.0753702	3.2298005
O	1.3632145	1.2477505	3.3214852
O	-1.3632261	1.2477547	3.3215171
C	-1.2062055	-2.4956914	3.2513445
H	-2.1357018	-3.0753611	3.2298267
C	-0.0000001	-3.1816693	3.2037134
H	-0.0000098	-4.2603112	3.1421068
C	1.2062044	-2.4957118	3.2512932
H	2.1357049	-3.0753874	3.2299004
O	-1.3632222	-1.2477509	3.3215117
O	1.3632177	-1.2477549	3.3212978
Ni	-0.0000063	-0.0000020	3.3334014
I	0.0000042	-0.0000098	0.0206715
C	-0.0000105	0.0000031	-2.0545093
C	-1.1917904	0.0000030	-2.7648364
C	1.1917768	0.0000054	-2.7648197
C	-1.1993187	0.0000043	-4.1502774
F	-2.3569306	0.0000012	-2.1349197
C	1.1993338	0.0000069	-4.1502568
F	2.3569047	0.0000055	-2.1348823
C	0.0000142	0.0000061	-4.8436562
F	-2.3438359	0.0000035	-4.8162767
F	2.3438663	0.0000084	-4.8162289
F	0.0000223	0.0000066	-6.1652459

1B:C_6F5_I

C	1.2300171	3.0415665	-2.8832664

H	2.1056901	3.6869578	-2.8597361
C	0.0000004	3.6704040	-2.8423372
H	-0.0000000	4.7532535	-2.7836622
C	-1.2300105	3.0415538	-2.8832868
H	-2.1056943	3.6869591	-2.8597761
C	-1.2300125	-3.0415529	-2.8832778
H	-2.1056943	-3.6869589	-2.8597590
C	0.0000029	-3.6704075	-2.8423385
H	-0.0000033	-4.7532523	-2.7836624
C	1.2300156	-3.0415626	-2.8832760
H	2.1056916	-3.6869592	-2.8597528
S	-1.6417819	1.4333186	-2.9657619
S	1.6417778	1.4333150	-2.9657343
S	-1.6417808	-1.4333190	-2.9657508
S	1.6417784	-1.4333170	-2.9657447
Ni	-0.0000036	-0.0000002	-2.9803165
I	-0.0000041	-0.0000007	0.5405828
C	-0.0000144	-0.0000001	2.6160983
C	1.1913921	0.0000001	3.3271473
C	-1.1913977	-0.0000000	3.3271586
C	1.1991872	0.0000003	4.7125550
F	2.3569007	0.0000000	2.6973982
C	-1.1991772	0.0000002	4.7125820
F	-2.3569269	-0.0000001	2.6974273
C	0.0000047	0.0000003	5.4060943
F	2.3438852	0.0000004	5.3787283
F	-2.3438631	0.0000002	5.3787636
F	0.0000211	0.0000004	6.7279583

2A:C_6F5_I

C	2.4996290	-1.2159909	3.2071301
H	3.1065488	-2.1271556	3.2072258
C	3.1617778	0.0000000	3.2078008
C	2.4996290	1.2159909	3.2071301
H	3.1065488	2.1271556	3.2072258

O	1.2478504	-1.3666900	3.2070725
O	1.2478504	1.3666900	3.2070725
C	-2.4996290	1.2159909	3.2071301
H	-3.1065488	2.1271556	3.2072258
C	-3.1617778	0.0000000	3.2078008
C	-2.4996290	-1.2159909	3.2071301
H	-3.1065488	-2.1271556	3.2072258
O	-1.2478504	1.3666900	3.2070725
O	-1.2478504	-1.3666900	3.2070725
Ni	0.0000000	-0.0000000	3.2119610
F	4.5098534	-0.0000000	3.2080453
F	-4.5098534	-0.0000000	3.2080453
I	-0.0000000	-0.0000000	-0.5387882
C	-0.0000000	-0.0000000	-2.6107007
C	0.0000000	1.1926666	-3.3206109
C	0.0000000	-1.1926666	-3.3206108
C	0.0000000	1.1994123	-4.7062434
F	0.0000000	2.3569856	-2.6902811
C	0.0000000	-1.1994123	-4.7062434
F	-0.0000000	-2.3569856	-2.6902812
C	-0.0000000	-0.0000000	-5.3992683
F	-0.0000000	2.3431079	-5.3725225
F	-0.0000000	-2.3431079	-5.3725225
F	0.0000000	0.0000000	-6.7201793

2B:C_6F5_I

C	3.0363740	-1.2364193	2.7271518
H	3.7129414	-2.0877281	2.7045269
C	3.6390543	-0.0000011	2.6923675
C	3.0363796	1.2364195	2.7271411
H	3.7129437	2.0877396	2.7045080
C	-3.0363795	1.2364182	2.7271372
H	-3.7129450	2.0877400	2.7045066
C	-3.6390556	-0.0000010	2.6923636
C	-3.0363730	-1.2364187	2.7271500

H	-3.7129432	-2.0877290	2.7045277
F	4.9874895	-0.0000035	2.6267093
F	-4.9874902	-0.0000030	2.6267047
S	1.4283120	1.6462617	2.7987504
S	1.4283198	-1.6462578	2.7987674
S	-1.4283132	1.6462612	2.7987520
S	-1.4283217	-1.6462581	2.7987704
Ni	-0.0000002	-0.0000087	2.8135459
I	-0.0000002	-0.0000228	-0.7291061
C	0.0000003	-0.0000075	-2.8038017
C	0.0000005	1.1918995	-3.5138457
C	0.0000005	-1.1919073	-3.5138586
C	0.0000008	1.1994651	-4.8992536
F	0.0000004	2.3566965	-2.8831687
C	0.0000008	-1.1994591	-4.8992645
F	0.0000004	-2.3567079	-2.8831914
C	0.0000010	0.0000066	-5.5925556
F	0.0000009	2.3437959	-5.5651342
F	0.0000008	-2.3437825	-5.5651552
F	0.0000012	0.0000117	-6.9139068

3A:C_6F5_I

C	2.4977712	-1.2184201	3.1057421
H	3.0834945	-2.1432547	3.1025119
C	3.1924662	-0.0000001	3.1050710
C	2.4977712	1.2184202	3.1057421
H	3.0834945	2.1432547	3.1025119
O	1.2540409	-1.3585421	3.1098696
O	1.2540409	1.3585421	3.1098696
C	-2.4978172	1.2184046	3.1057419
H	-3.0834918	2.1432528	3.1025117
C	-3.1924517	-0.0000000	3.1050713
C	-2.4978172	-1.2184045	3.1057419
H	-3.0834919	-2.1432528	3.1025117
O	-1.2540126	1.3585451	3.1098693

O	-1.2540127	-1.3585451	3.1098693
Ni	0.0000064	-0.0000000	3.1162740
C	-4.6120354	-0.0000000	3.1051584
C	4.6120383	0.0000000	3.1051574
N	5.7644820	0.0000000	3.1073769
N	-5.7644761	-0.0000000	3.1073769
I	0.0000004	0.0000000	-0.6378516
C	0.0000000	0.0000002	-2.7087018
C	0.0000000	1.1934906	-3.4166067
C	0.0000000	-1.1934909	-3.4166068
C	0.0000000	1.1999709	-4.8021477
F	0.0000000	2.3566997	-2.7848644
C	0.0000000	-1.1999704	-4.8021477
F	-0.0000000	-2.3566996	-2.7848644
C	0.0000001	-0.0000004	-5.4949938
F	0.0000000	2.3431665	-5.4675140
F	0.0000000	-2.3431666	-5.4675141
F	-0.0000000	0.0000000	-6.8149595

3B:C_6F5_I

C	-3.0334054	1.2384846	-2.6687851
H	-3.6836692	2.1101738	-2.6420339
C	-3.6702837	0.0000121	-2.6221268
C	-3.0334422	-1.2384969	-2.6687901
H	-3.6836628	-2.1101638	-2.6420438
C	3.0334570	-1.2384929	-2.6688149
H	3.6836749	-2.1101539	-2.6420521
C	3.6702640	-0.0000058	-2.6221205
C	3.0334261	1.2385043	-2.6688087
H	3.6836737	2.1101555	-2.6420386
Ni	0.0000085	0.0000046	-2.7779894
C	5.0963355	0.0000017	-2.5364544
C	-5.0963568	0.0000006	-2.5365244
N	-6.2463507	0.0000031	-2.4684646
N	6.2463246	0.0000027	-2.4684052

S	1.4324514	-1.6395596	-2.7616374
S	1.4324686	1.6395556	-2.7616307
S	-1.4324647	1.6395463	-2.7615437
S	-1.4324401	-1.6395529	-2.7615499
I	-0.0000020	-0.0000018	0.7885352
C	0.0000004	0.0000141	2.8617060
C	-0.0000004	-1.1928473	3.5699366
C	0.0000003	1.1928465	3.5698933
C	-0.0000012	-1.1999884	4.9553079
F	-0.0000007	-2.3563721	2.9375166
C	-0.0000005	1.1999578	4.9552967
F	0.0000004	2.3563445	2.9375266
C	-0.0000011	0.0000025	5.6481978
F	-0.0000019	-2.3436432	5.6206448
F	-0.0000007	2.3436650	5.6206445
F	-0.0000014	0.0000034	6.9686008

4A:C_6F5_I

C	2.4981927	-1.2241230	2.8853498
H	3.0976872	-2.1369978	2.8825986
C	3.1715966	0.0000091	2.8847803
C	2.4981852	1.2241131	2.8853498
H	3.0976878	2.1369990	2.8825986
O	1.2536718	-1.3603132	2.8887529
O	1.2536800	1.3603146	2.8887529
C	-2.4981902	1.2241166	2.8853498
H	-3.0976874	2.1369983	2.8825986
C	-3.1715960	0.0000027	2.8847803
C	-2.4981928	-1.2241194	2.8853498
H	-3.0976873	-2.1369981	2.8825986
O	-1.2536751	1.3603143	2.8887529
O	-1.2536722	-1.3603138	2.8887529
Ni	-0.0000002	-0.0000007	2.8951394
N	-4.6145746	-0.0000086	2.8849328
N	4.6145753	-0.0000352	2.8849328

O	-5.1777646	-1.0760823	2.8854280
O	-5.1777685	1.0760904	2.8854280
O	5.1777581	-1.0760691	2.8854279
O	5.1777743	1.0761028	2.8854280
I	-0.0000000	-0.0000000	-0.8727125
C	0.0000000	-0.0000009	-2.9431627
C	-0.0000001	1.1935547	-3.6507164
C	-0.0000000	-1.1935531	-3.6507154
C	0.0000000	1.1999788	-5.0361427
F	0.0000000	2.3564663	-3.0189220
C	0.0000000	-1.1999816	-5.0361429
F	0.0000000	-2.3564665	-3.0189220
C	-0.0000000	0.0000020	-5.7289820
F	-0.0000000	2.3432578	-5.7011429
F	-0.0000000	-2.3432571	-5.7011425
F	0.0000000	-0.0000003	-7.0487686

4B:C_6F5_I

C	3.0317772	-1.2399574	2.5276489
H	3.7051327	-2.0930801	2.5254030
C	3.6436869	0.0000061	2.5418010
C	3.0317926	1.2398271	2.5276498
H	3.7052410	2.0929351	2.5253996
C	-3.0317857	1.2398834	2.5276484
H	-3.7052425	2.0929106	2.5253982
C	-3.6436866	-0.0000885	2.5418011
C	-3.0317898	-1.2398957	2.5276504
H	-3.7051311	-2.0931047	2.5254042
N	-5.1181428	0.0000458	2.5678140
N	5.1181821	0.0000192	2.5678146
O	-5.6795677	-1.0738615	2.5794472
O	-5.6794900	1.0739974	2.5794263
O	5.6795588	-1.0738449	2.5794474
O	5.6794606	1.0739649	2.5794253
S	1.4305351	1.6434722	2.5112125

S	1.4304949	-1.6433691	2.5112033
S	-1.4304848	-1.6433801	2.5112010
S	-1.4305407	1.6434637	2.5112148
Ni	-0.0000000	0.0000796	2.5286382
I	-0.0000001	-0.0000104	-1.0544580
C	0.0000000	-0.0000114	-3.1276561
C	0.0000001	-1.1928345	-3.8360834
C	-0.0000000	1.1928298	-3.8360693
C	-0.0000000	-1.1999389	-5.2214720
F	0.0000002	-2.3565454	-3.2042771
C	-0.0000000	1.1999656	-5.2214816
F	-0.0000001	2.3565194	-3.2042443
C	-0.0000000	0.0000008	-5.9145224
F	-0.0000001	-2.3440146	-5.8861285
F	0.0000001	2.3440141	-5.8861140
F	-0.0000000	0.0000025	-7.2348955

5A:C_6F5_I

C	2.5032365	-1.1973807	3.0207686
H	3.0809815	-2.1303783	3.0067018
C	3.2069512	0.0001053	2.9555273
C	2.5031861	1.1975693	3.0208868
H	3.0808245	2.1306274	3.0072567
O	1.2512793	-1.3503081	3.1007786
O	1.2512388	1.3503542	3.1004626
C	-2.5031935	1.1975365	3.0208801
H	-3.0808379	2.1306546	3.0071347
C	-3.2069636	0.0001076	2.9555554
C	-2.5032392	-1.1973773	3.0207860
H	-3.0809993	-2.1303702	3.0068194
O	-1.2512753	1.3503521	3.1005806
O	-1.2512614	-1.3503220	3.1006344
Ni	-0.0000066	-0.0000065	3.1109007
N	-4.6286203	-0.0001068	2.9382170
N	4.6286285	0.0000391	2.9381915

H	-4.9946751	0.8143589	2.4649048
H	-4.9943097	-0.8147698	2.4648841
H	4.9946120	0.8142839	2.4645284
H	4.9944203	-0.8148477	2.4653229
I	0.0000359	0.0001337	-0.1510386
C	0.0000047	-0.0000324	-2.2286926
C	0.0000039	1.1912875	-2.9395888
C	-0.0000044	-1.1913780	-2.9395888
C	0.0000004	1.1992339	-4.3250314
F	0.0000087	2.3568820	-2.3098553
C	-0.0000061	-1.1992774	-4.3250670
F	-0.0000089	-2.3569772	-2.3099073
C	-0.0000022	-0.0000031	-5.0185927
F	0.0000015	2.3439644	-4.9910380
F	-0.0000093	-2.3439612	-4.9911076
F	-0.0000009	0.0000062	-6.3403318

5B:C_6F5_I

C	3.0461748	-1.2193614	2.7588725
H	3.6813883	-2.1045650	2.7464908
C	3.7033939	-0.0000189	2.6849967
C	3.0453448	1.2189010	2.7580256
H	3.6800241	2.1044679	2.7447076
C	-3.0454652	1.2184569	2.7577181
H	-3.6798809	2.1041945	2.7438509
C	-3.7034747	-0.0004555	2.6849028
C	-3.0460068	-1.2197125	2.7589715
H	-3.6811629	-2.1049577	2.7474662
N	-5.1115956	-0.0006559	2.6307351
N	5.1115125	0.0007772	2.6308508
H	-5.4950515	0.8276231	2.2013999
H	-5.4945651	-0.8211500	2.1862407
H	5.4940157	0.8260785	2.1949587
H	5.4953236	-0.8227780	2.1928273
S	1.4362290	1.6230822	2.8771997

S	1.4371351	-1.6244322	2.8777172
S	-1.4363495	1.6227131	2.8776875
S	-1.4368608	-1.6244855	2.8766263
Ni	0.0000702	-0.0009944	2.8790415
I	-0.0000621	0.0001245	-0.5587126
C	0.0000354	-0.0000897	-2.6377330
C	0.0000328	1.1905900	-3.3498072
C	0.0000320	-1.1909164	-3.3495640
C	-0.0000250	1.1986774	-4.7352689
F	0.0000714	2.3569397	-2.7207458
C	-0.0000261	-1.1992873	-4.7350237
F	0.0000670	-2.3571317	-2.7202577
C	-0.0000758	-0.0003739	-5.4289244
F	-0.0000391	2.3435852	-5.4017461
F	-0.0000409	-2.3443366	-5.4012654
F	-0.0001686	-0.0005087	-6.7511232

6A:C_6F5_I

C	2.5102195	-1.1956728	2.5622394
H	3.0604508	-2.1386116	2.5673824
C	3.2274126	-0.0001422	2.4451726
C	2.5102895	1.1954734	2.5623277
H	3.0605159	2.1383776	2.5675259
O	1.2583261	-1.3346879	2.6762358
O	1.2583635	1.3345099	2.6763090
C	-2.5104532	1.1955581	2.5620474
H	-3.0606173	2.1384892	2.5670655
C	-3.2276237	-0.0000373	2.4450250
C	-2.5104521	-1.1956261	2.5622703
H	-3.0606477	-2.1385713	2.5674853
O	-1.2585137	1.3345514	2.6761083
O	-1.2585825	-1.3346277	2.6763182
Ni	-0.0001088	-0.0000554	2.6728487
N	-4.6269721	0.0000528	2.2223835
N	4.6268020	-0.0001544	2.2225740

C	-5.3257663	-1.2099543	2.5784210
H	-5.1852218	-1.5062143	3.6320405
H	-6.3916311	-1.0638013	2.4040426
H	-5.0130913	-2.0447672	1.9461359
C	-5.3258366	1.2099177	2.5789048
H	-5.0133215	2.0449983	1.9468747
H	-6.3916896	1.0637834	2.4045904
H	-5.1852080	1.5058314	3.6326161
C	5.3256531	1.2097494	2.5788368
H	5.1850848	1.5058256	3.6325138
H	6.3915075	1.0636148	2.4045110
H	5.0130843	2.0446940	1.9466543
C	5.3255724	-1.2100912	2.5789201
H	5.0129936	-2.0450190	1.9467325
H	6.3914594	-1.0639584	2.4046242
H	5.1849724	-1.5061422	3.6325825
I	-0.0000530	-0.0001721	-0.6127513
C	0.0000305	0.0000179	-2.6907759
C	0.0000123	-1.1911621	-3.4023415
C	0.0000864	1.1912509	-3.4022691
C	0.0000541	-1.1990298	-4.7879049
F	-0.0000568	-2.3571936	-2.7733793
C	0.0001367	1.1991817	-4.7878379
F	0.0000793	2.3572652	-2.7732418
C	0.0001191	0.0001027	-5.4814923
F	0.0000219	-2.3439069	-5.4543088
F	0.0001931	2.3440829	-5.4541880
F	0.0001557	0.0001416	-6.8035905

6B:C_6F5_I

C	3.0593667	-1.2147779	-2.4377648
H	3.6595017	-2.1175939	-2.4429659
C	3.7444991	-0.0002004	-2.4213524
C	3.0594584	1.2144721	-2.4377921
H	3.6596658	2.1171942	-2.4430159

C	-3.0595634	1.2146436	-2.4376258
H	-3.6597305	2.1173908	-2.4427286
C	-3.7447062	-0.0000113	-2.4214434
C	-3.0596345	-1.2146558	-2.4380048
H	-3.6598293	-2.1174312	-2.4434682
N	-5.1475792	0.0000576	-2.3765239
N	5.1473725	-0.0002876	-2.3763110
C	-5.8335706	1.2222414	-2.7149055
H	-5.5727192	1.6055199	-3.7139924
H	-6.9070167	1.0413032	-2.6907752
H	-5.6294369	2.0093088	-1.9839835
C	-5.8336269	-1.2220130	-2.7149383
H	-5.6294725	-2.0091614	-1.9840608
H	-6.9070664	-1.0410755	-2.6907470
H	-5.5728546	-1.6052760	-3.7140640
C	5.8334073	-1.2223769	-2.7146369
H	5.5726723	-1.6057124	-3.7137576
H	6.9068271	-1.0414701	-2.6903991
H	5.6291693	-2.0095100	-1.9837249
C	5.8334666	1.2219119	-2.7144399
H	5.6292351	2.0088783	-1.9834115
H	6.9068858	1.0409323	-2.6902016
H	5.5727569	1.6053615	-3.7134851
S	-1.4420306	-1.6078160	-2.4414366
S	-1.4419850	1.6077341	-2.4411232
S	1.4417415	-1.6078728	-2.4413952
S	1.4418828	1.6076721	-2.4413828
Ni	-0.0000989	-0.0000343	-2.4415634
I	-0.0000701	-0.0003593	1.0601418
C	0.0000212	-0.0001132	3.1386207
C	0.0000036	-1.1907431	3.8512285
C	0.0000628	1.1906904	3.8509424
C	0.0000290	-1.1987405	5.2367404
F	-0.0000228	-2.3574363	3.2229139
C	0.0001615	1.1990063	5.2364605

F	0.0000394	2.3572292	3.2223706
C	0.0001408	0.0002119	5.9303944
F	-0.0000464	-2.3438578	5.9035063
F	0.0002682	2.3442736	5.9029684
F	0.0001745	0.0003641	7.2529267

7A:C_6F5_I

C	2.5076713	-1.1917807	-2.5392161
H	3.0615320	-2.1345942	-2.5362797
C	3.2311065	-0.0000217	-2.5216538
C	2.5076872	1.1918071	-2.5392127
H	3.0615296	2.1345857	-2.5362814
O	1.2548394	-1.3431709	-2.5575645
O	1.2548218	1.3431687	-2.5575688
C	-2.5076773	1.1917952	-2.5392151
H	-3.0615319	2.1345922	-2.5362845
C	-3.2311088	0.0000042	-2.5216521
C	-2.5076780	-1.1918023	-2.5392154
H	-3.0615266	-2.1345853	-2.5362882
O	-1.2548304	1.3431704	-2.5575666
O	-1.2548288	-1.3431688	-2.5575661
Ni	0.0000012	-0.0000029	-2.5617067
C	-4.7595876	-0.0000048	-2.5702807
C	4.7595881	0.0000016	-2.5702818
C	-5.3379992	1.2335156	-1.8703653
H	-5.1045328	2.1615020	-2.3964957
H	-6.4272748	1.1568149	-1.8341406
H	-4.9688842	1.3179045	-0.8453081
C	-5.3380128	-1.2335069	-1.8703644
H	-6.4272658	-1.1568163	-1.8341408
H	-5.1045293	-2.1615069	-2.3964994
H	-4.9688810	-1.3179048	-0.8453068
C	-5.2177909	0.0000060	-4.0331588
H	-6.3098032	-0.0000012	-4.1021585
H	-4.8434715	0.8827141	-4.5578888

H	-4.8434689	-0.8827200	-4.5578913
C	5.2177912	0.0000031	-4.0331600
H	4.8434697	0.8827148	-4.5578899
H	6.3098021	-0.0000015	-4.1021603
H	4.8434678	-0.8827195	-4.5578914
C	5.3380042	1.2335129	-1.8703664
H	6.4272736	1.1568184	-1.8341428
H	5.1045306	2.1615038	-2.3964972
H	4.9688857	1.3179029	-0.8453077
C	5.3380096	-1.2335086	-1.8703651
H	4.9688842	-1.3179027	-0.8453077
H	5.1045288	-2.1615058	-2.3964998
H	6.4272684	-1.1568193	-1.8341428
I	-0.0000004	-0.0000013	0.7345957
C	-0.0000007	0.0000025	2.8114968
C	-0.0000008	-1.1913233	3.5226369
C	-0.0000007	1.1913134	3.5226332
C	-0.0000008	-1.1991522	4.9080866
F	-0.0000011	-2.3570148	2.8934993
C	-0.0000007	1.1991597	4.9080881
F	-0.0000009	2.3570254	2.8934982
C	-0.0000007	-0.0000112	5.6015124
F	-0.0000010	-2.3438774	5.5742749
F	-0.0000007	2.3438849	5.5742814
F	-0.0000006	0.0000012	6.9232727

7B:C_6F5_I

C	3.0414722	-1.2150360	-2.4425565
H	3.6504598	-2.1135158	-2.4124211
C	3.7107327	-0.0063798	-2.3175354
C	3.0464105	1.2040978	-2.4530970
H	3.6594799	2.1001215	-2.4322661
C	-3.0414299	1.2150526	-2.4424294
H	-3.6503780	2.1135529	-2.4121659
C	-3.7107328	0.0064139	-2.3175441

C	-3.0464441	-1.2040776	-2.4532401
H	-3.6595584	-2.1000851	-2.4325561
C	-5.2392017	0.0074668	-2.1421917
C	5.2392111	-0.0074463	-2.1421908
C	-5.7117574	1.2432591	-1.3714543
H	-5.5728641	2.1691751	-1.9330513
H	-6.7815547	1.1584042	-1.1681219
H	-5.1911245	1.3393101	-0.4154648
C	-5.7107366	-1.2214794	-1.3595577
H	-6.7806898	-1.1359654	-1.1571102
H	-5.5707292	-2.1526887	-1.9119664
H	-5.1897727	-1.3078107	-0.4028965
C	-5.8922135	0.0010192	-3.5281368
H	-6.9823254	0.0010022	-3.4409983
H	-5.5958371	0.8815892	-4.1036436
H	-5.5954248	-0.8843392	-4.0960653
C	5.8922064	-0.0010822	-3.5281418
H	5.5954930	0.8843009	-4.0960801
H	6.9823231	-0.0011310	-3.4410107
H	5.5957669	-0.8816320	-4.1036280
C	5.7107866	1.2215036	-1.3596046
H	6.7807470	1.1359810	-1.1571667
H	5.5708134	2.1527049	-1.9120703
H	5.1898359	1.3078801	-0.4029514
C	5.7117234	-1.2432270	-1.3714019
H	5.1910911	-1.3392462	-0.4154205
H	5.5728028	-2.1691637	-1.9329612
H	6.7815237	-1.1583851	-1.1680803
S	-1.4400690	1.6176423	-2.6485856
S	-1.4466850	-1.6116160	-2.6598845
S	1.4401083	-1.6175525	-2.6487804
S	1.4466552	1.6117130	-2.6596756
Ni	0.0000059	0.0000471	-2.6497417
I	-0.0000656	-0.0001980	0.7704788
C	-0.0000406	-0.0000986	2.8490855

C	-0.1079706	-1.1857911	3.5613629
C	0.1079279	1.1856556	3.5612446
C	-0.1075788	-1.1940692	4.9467373
F	-0.2155125	-2.3476539	2.9335745
C	0.1075902	1.1940833	4.9466140
F	0.2154352	2.3474665	2.9333246
C	0.0000140	0.0000451	5.6407380
F	-0.2100058	-2.3353688	5.6115592
F	0.2100451	2.3354500	5.6113147
F	0.0000414	0.0001015	6.9629201

8A:C_6F5_I

C	-2.5829911	1.2286493	2.7975251
H	-3.2075079	2.1289953	2.7809208
C	-3.2344082	-0.0000000	2.7667804
H	-4.3145852	0.0000000	2.7222959
C	-2.5829911	-1.2286493	2.7975251
H	-3.2075079	-2.1289953	2.7809208
O	-1.3458692	1.4578519	2.8451151
O	-1.3458692	-1.4578519	2.8451151
C	2.5829911	-1.2286493	2.7975251
H	3.2075079	-2.1289953	2.7809208
C	3.2344082	0.0000000	2.7667804
H	4.3145852	-0.0000000	2.7222959
C	2.5829911	1.2286493	2.7975251
H	3.2075079	2.1289953	2.7809208
O	1.3458692	-1.4578519	2.8451151
O	1.3458692	1.4578519	2.8451151
Pd	0.0000000	0.0000000	2.8557893
I	-0.0000000	0.0000000	-0.5392565
C	-0.0000000	0.0000000	-2.6170800
C	-0.0000000	-1.1915018	-3.3278214
C	-0.0000000	1.1915018	-3.3278214
C	0.0000000	-1.1993281	-4.7132385
F	0.0000000	-2.3569756	-2.6980116

C	0.0000000	1.1993281	-4.7132385
F	0.0000000	2.3569756	-2.6980116
C	0.0000000	0.0000000	-5.4064629
F	-0.0000000	-2.3438682	-5.3795534
F	0.0000000	2.3438682	-5.3795534
F	-0.0000000	0.0000000	-6.7281367

8B:C_6F5_I

C	3.1068934	1.2432872	2.8222445
H	3.7823956	2.0966184	2.7954738
C	3.7144119	0.0000000	2.7817092
H	4.7974922	0.0000000	2.7210038
C	3.1068934	-1.2432872	2.8222445
H	3.7823956	-2.0966184	2.7954738
C	-3.1068934	-1.2432872	2.8222445
H	-3.7823956	-2.0966184	2.7954738
C	-3.7144119	0.0000000	2.7817092
H	-4.7974922	0.0000000	2.7210038
C	-3.1068934	1.2432872	2.8222445
H	-3.7823956	2.0966184	2.7954738
S	1.5197157	-1.7230203	2.9072411
S	1.5197157	1.7230203	2.9072411
S	-1.5197157	-1.7230203	2.9072411
S	-1.5197157	1.7230203	2.9072411
Pd	0.0000000	0.0000000	2.9231217
I	0.0000000	0.0000000	-0.5799227
C	0.0000000	0.0000000	-2.6581167
C	0.0000000	-1.1910470	-3.3695031
C	0.0000000	1.1910470	-3.3695031
C	0.0000000	-1.1991273	-4.7549589
F	0.0000000	-2.3569476	-2.7399195
C	0.0000000	1.1991273	-4.7549589
F	0.0000000	2.3569476	-2.7399195
C	0.0000000	0.0000000	-5.4485696
F	-0.0000000	-2.3439474	-5.4212122

F	-0.0000000	2.3439474	-5.4212122
F	0.0000000	0.0000000	-6.7705887

9A:C_6F5_I

C	2.5818690	-1.2366121	2.8128890
H	3.2333475	-2.1164536	2.7910869
C	3.2097008	0.0000000	2.7787036
C	2.5818690	1.2366121	2.8128890
H	3.2333475	2.1164536	2.7910869
O	1.3430166	-1.4600615	2.8687164
O	1.3430166	1.4600615	2.8687164
C	-2.5818690	1.2366121	2.8128890
H	-3.2333475	2.1164536	2.7910869
C	-3.2097008	0.0000000	2.7787036
C	-2.5818690	-1.2366121	2.8128890
H	-3.2333475	-2.1164536	2.7910869
O	-1.3430166	1.4600615	2.8687164
O	-1.3430166	-1.4600615	2.8687164
F	4.5594761	0.0000000	2.7171476
F	-4.5594761	-0.0000000	2.7171476
Pd	-0.0000000	0.0000000	2.8805220
I	0.0000000	0.0000000	-0.5620489
C	-0.0000000	0.0000000	-2.6378585
C	-0.0000000	1.1921528	-3.3472870
C	-0.0000000	-1.1921528	-3.3472870
C	0.0000000	1.1996171	-4.7327179
F	-0.0000000	2.3567302	-2.7164970
C	0.0000000	-1.1996171	-4.7327179
F	-0.0000000	-2.3567302	-2.7164970
C	0.0000000	0.0000000	-5.4258353
F	0.0000000	2.3437467	-5.3986240
F	0.0000000	-2.3437467	-5.3986240
F	-0.0000000	0.0000000	-6.7469988

9B:C_6F5_I

C	3.1008745	1.2484419	-2.8375133
H	3.8069796	2.0761801	-2.8133816
C	3.6836844	0.0000000	-2.8053424
C	3.1008745	-1.2484419	-2.8375133
H	3.8069796	-2.0761801	-2.8133816
C	-3.1008745	-1.2484419	-2.8375133
H	-3.8069796	-2.0761801	-2.8133816
C	-3.6836844	-0.0000000	-2.8053424
C	-3.1008745	1.2484419	-2.8375133
H	-3.8069796	2.0761801	-2.8133816
F	5.0350782	0.0000000	-2.7413778
F	-5.0350782	0.0000000	-2.7413778
S	1.5136041	-1.7270698	-2.9073164
S	1.5136041	1.7270698	-2.9073164
S	-1.5136041	-1.7270698	-2.9073164
S	-1.5136041	1.7270698	-2.9073164
Pd	0.0000000	0.0000000	-2.9233842
I	0.0000000	0.0000000	0.6005376
C	0.0000000	0.0000000	2.6776468
C	0.0000000	-1.1916131	3.3879741
C	0.0000000	1.1916131	3.3879741
C	-0.0000000	-1.1994393	4.7734084
F	-0.0000000	-2.3567604	2.7574450
C	-0.0000000	1.1994393	4.7734084
F	-0.0000000	2.3567604	2.7574450
C	0.0000000	0.0000000	5.4667257
F	0.0000000	-2.3438505	5.4394424
F	0.0000000	2.3438505	5.4394424
F	0.0000000	0.0000000	6.7882200

10A:C_6F5_I

C	-2.5835875	1.2398685	2.6573521
H	-3.2105395	2.1371388	2.6316432
C	-3.2440699	0.0000000	2.6098645
C	-2.5835875	-1.2398685	2.6573521

H	-3.2105395	-2.1371388	2.6316432
O	-1.3523280	1.4489866	2.7297879
O	-1.3523280	-1.4489866	2.7297879
C	2.5835875	-1.2398685	2.6573521
H	3.2105395	-2.1371388	2.6316432
C	3.2440699	0.0000000	2.6098645
C	2.5835875	1.2398685	2.6573521
H	3.2105395	2.1371388	2.6316432
O	1.3523280	-1.4489866	2.7297879
O	1.3523280	1.4489866	2.7297879
Pd	0.0000000	0.0000000	2.7446181
C	4.6635764	-0.0000000	2.5244667
C	-4.6635764	-0.0000000	2.5244667
N	5.8139135	0.0000000	2.4558123
N	-5.8139135	-0.0000000	2.4558123
I	0.0000000	0.0000000	-0.7529363
C	0.0000000	0.0000000	-2.8264413
C	0.0000000	-1.1933205	-3.5337595
C	0.0000000	1.1933205	-3.5337595
C	0.0000000	-1.2002333	-4.9191524
F	-0.0000000	-2.3564211	-2.9011272
C	0.0000000	1.2002333	-4.9191524
F	-0.0000000	2.3564211	-2.9011272
C	0.0000000	0.0000000	-5.6117701
F	0.0000000	-2.3436232	-5.5844681
F	0.0000000	2.3436232	-5.5844681
F	-0.0000000	0.0000000	-6.9318758

10B:C_6F5_I

C	3.1011599	-1.2513943	2.6623344
H	3.7796883	-2.1019204	2.6323240
C	3.7184620	0.0000000	2.6137840
C	3.1011599	1.2513943	2.6623344
H	3.7796883	2.1019204	2.6323240
C	-3.1011599	1.2513943	2.6623344

H	-3.7796883	2.1019204	2.6323240
C	-3.7184620	0.0000000	2.6137840
C	-3.1011599	-1.2513943	2.6623344
H	-3.7796883	-2.1019204	2.6323240
S	1.5205800	1.7183621	2.7603749
S	1.5205800	-1.7183621	2.7603749
S	-1.5205800	-1.7183621	2.7603749
S	-1.5205800	1.7183621	2.7603749
Pd	0.0000000	0.0000000	2.7790506
C	-5.1452802	0.0000000	2.5187310
C	5.1452802	0.0000000	2.5187310
N	-6.2947779	0.0000000	2.4413533
N	6.2947779	0.0000000	2.4413533
I	0.0000000	0.0000000	-0.7623131
C	0.0000000	0.0000000	-2.8375986
C	0.0000000	1.1925065	-3.5461801
C	0.0000000	-1.1925065	-3.5461801
C	0.0000000	1.1999328	-4.9315526
F	-0.0000000	2.3564860	-2.9141583
C	0.0000000	-1.1999328	-4.9315526
F	0.0000000	-2.3564860	-2.9141583
C	0.0000000	0.0000000	-5.6244361
F	0.0000000	2.3437390	-5.5969158
F	0.0000000	-2.3437390	-5.5969158
F	0.0000000	0.0000000	-6.9449589

11A:C_6F5_I

C	2.5844656	-1.2438433	2.5030668
H	3.2230512	-2.1293848	2.4720034
C	3.2250266	0.0000000	2.4420832
C	2.5844656	1.2438433	2.5030668
H	3.2230512	2.1293848	2.4720034
O	1.3533490	-1.4479268	2.5939452
O	1.3533490	1.4479268	2.5939452
C	-2.5844656	1.2438433	2.5030668

H	-3.2230512	2.1293848	2.4720034
C	-3.2250266	0.0000000	2.4420832
C	-2.5844656	-1.2438433	2.5030668
H	-3.2230512	-2.1293848	2.4720034
O	-1.3533490	1.4479268	2.5939452
O	-1.3533490	-1.4479268	2.5939452
N	-4.6701669	0.0000000	2.3276522
N	4.6701669	0.0000000	2.3276522
O	-5.2333160	-1.0747286	2.2821698
O	-5.2333160	1.0747286	2.2821698
O	5.2333160	-1.0747286	2.2821698
O	5.2333160	1.0747286	2.2821698
Pd	0.0000000	0.0000000	2.6103373
I	0.0000000	0.0000000	-0.8827550
C	0.0000000	0.0000000	-2.9562777
C	0.0000000	1.1934909	-3.6632996
C	0.0000000	-1.1934909	-3.6632996
C	-0.0000000	1.2003116	-5.0487069
F	0.0000000	2.3562915	-3.0303839
C	-0.0000000	-1.2003116	-5.0487069
F	-0.0000000	-2.3562915	-3.0303839
C	-0.0000000	0.0000000	-5.7412079
F	-0.0000000	2.3435080	-5.7141503
F	0.0000000	-2.3435080	-5.7141503
F	0.0000000	0.0000000	-7.0612277

11B:C_6F5_I

C	3.1194531	-1.2538093	2.4966433
H	3.8343371	-2.0895541	2.4754000
C	3.7455869	-0.0000000	2.4543405
C	3.1194531	1.2538093	2.4966433
H	3.8343371	2.0895541	2.4754000
C	-3.1194531	1.2538093	2.4966433
H	-3.8343371	2.0895541	2.4754000
C	-3.7455869	0.0000000	2.4543405

C	-3.1194531	-1.2538093	2.4966433
H	-3.8343371	-2.0895541	2.4754000
N	-5.0946098	0.0000000	2.3785733
N	5.0946098	-0.0000000	2.3785733
O	-5.7962141	-1.0682083	2.3412148
O	-5.7962141	1.0682083	2.3412148
O	5.7962141	-1.0682083	2.3412148
O	5.7962141	1.0682083	2.3412148
S	1.5475115	1.6826528	2.5691856
S	1.5475115	-1.6826528	2.5691856
S	-1.5475115	-1.6826528	2.5691856
S	-1.5475115	1.6826528	2.5691856
Pd	0.0000000	0.0000000	2.5821335
I	0.0000000	0.0000000	-0.8905485
C	0.0000000	0.0000000	-2.9762134
C	-0.0000000	1.1924782	-3.6830479
C	-0.0000000	-1.1924782	-3.6830479
C	0.0000000	1.2004236	-5.0684167
F	0.0000000	2.3559875	-3.0487716
C	0.0000000	-1.2004236	-5.0684167
F	0.0000000	-2.3559875	-3.0487716
C	-0.0000000	0.0000000	-5.7608974
F	0.0000000	2.3437854	-5.7342120
F	0.0000000	-2.3437854	-5.7342120
F	0.0000000	0.0000000	-7.0811801

12A:C_6F5_I

C	2.5927201	-1.2180465	2.5005043
H	3.2120892	-2.1240421	2.4821370
C	3.2647494	0.0000000	2.4264882
C	2.5927201	1.2180465	2.5005043
H	3.2120892	2.1240421	2.4821370
O	1.3526683	-1.4417748	2.5930659
O	1.3526683	1.4417748	2.5930659
C	-2.5927201	1.2180465	2.5005043

H	-3.2120892	2.1240421	2.4821370
C	-3.2647494	0.0000000	2.4264882
C	-2.5927201	-1.2180465	2.5005043
H	-3.2120892	-2.1240421	2.4821370
O	-1.3526683	1.4417748	2.5930659
O	-1.3526683	-1.4417748	2.5930659
N	-4.6907486	-0.0000000	2.3902313
N	4.6907486	-0.0000000	2.3902313
H	-5.0472008	0.8130904	1.9070055
H	-5.0472008	-0.8130904	1.9070055
H	5.0472008	0.8130904	1.9070055
H	5.0472008	-0.8130904	1.9070055
Pd	-0.0000000	0.0000000	2.6045948
I	0.0000000	0.0000000	-0.7560857
C	0.0000000	0.0000000	-2.8366081
C	-0.0000000	1.1910259	-3.5479969
C	-0.0000000	-1.1910259	-3.5479969
C	-0.0000000	1.1991689	-4.9335430
F	-0.0000000	2.3570338	-2.9185451
C	-0.0000000	-1.1991689	-4.9335430
F	0.0000000	-2.3570338	-2.9185451
C	0.0000000	0.0000000	-5.6271097
F	-0.0000000	2.3437273	-5.5999860
F	0.0000000	-2.3437273	-5.5999860
F	0.0000000	0.0000000	-6.9489389

12B:C_6F5_I

C	3.1163823	-1.2313661	2.5299218
H	3.7799110	-2.0962842	2.5147132
C	3.7557660	-0.0000000	2.4606950
C	3.1163823	1.2313661	2.5299218
H	3.7799110	2.0962842	2.5147132
C	-3.1163823	1.2313661	2.5299218
H	-3.7799110	2.0962842	2.5147132
C	-3.7557660	0.0000000	2.4606950

C	-3.1163823	-1.2313661	2.5299218
H	-3.7799110	-2.0962842	2.5147132
N	-5.1675326	0.0000000	2.4108024
N	5.1675326	-0.0000000	2.4108024
H	-5.5491929	0.8232082	1.9698568
H	-5.5491929	-0.8232082	1.9698568
H	5.5491929	0.8232082	1.9698568
H	5.5491929	-0.8232082	1.9698568
S	1.5273010	1.7047039	2.6456850
S	1.5273010	-1.7047039	2.6456850
S	-1.5273010	1.7047039	2.6456850
S	-1.5273010	-1.7047039	2.6456850
Pd	0.0000000	0.0000000	2.6526816
I	0.0000000	0.0000000	-0.8277667
C	0.0000000	0.0000000	-2.9081790
C	-0.0000000	1.1905489	-3.6203188
C	-0.0000000	-1.1905489	-3.6203188
C	0.0000000	1.1989618	-5.0057946
F	0.0000000	2.3570647	-2.9912934
C	0.0000000	-1.1989618	-5.0057946
F	0.0000000	-2.3570647	-2.9912934
C	-0.0000000	0.0000000	-5.6996146
F	0.0000000	2.3440717	-5.6720678
F	0.0000000	-2.3440717	-5.6720678
F	0.0000000	0.0000000	-7.0218742

13A:C_6F5_I

C	2.6081207	-1.2145358	1.7504963
H	3.1942859	-2.1348577	1.7476156
C	3.3007192	-0.0000000	1.6497360
C	2.6081207	1.2145358	1.7504963
H	3.1942859	2.1348577	1.7476156
O	1.3646066	-1.4212068	1.8526200
O	1.3646066	1.4212068	1.8526200
C	-2.6081207	1.2145358	1.7504963

H	-3.1942859	2.1348577	1.7476156
C	-3.3007192	-0.0000000	1.6497360
C	-2.6081207	-1.2145358	1.7504963
H	-3.1942859	-2.1348577	1.7476156
O	-1.3646066	1.4212068	1.8526200
O	-1.3646066	-1.4212068	1.8526200
N	-4.7082083	0.0000000	1.4435793
N	4.7082083	0.0000000	1.4435793
C	-5.4068735	-1.2084481	1.8073318
H	-5.2419602	-1.5161058	2.8541501
H	-6.4754986	-1.0509411	1.6641215
H	-5.1219206	-2.0408354	1.1588707
C	-5.4068735	1.2084481	1.8073318
H	-5.1219206	2.0408354	1.1588707
H	-6.4754986	1.0509411	1.6641215
H	-5.2419602	1.5161058	2.8541501
C	5.4068735	1.2084481	1.8073318
H	5.2419602	1.5161058	2.8541501
H	6.4754986	1.0509411	1.6641215
H	5.1219206	2.0408354	1.1588707
C	5.4068735	-1.2084481	1.8073318
H	5.1219206	-2.0408354	1.1588707
H	6.4754986	-1.0509411	1.6641215
H	5.2419602	-1.5161058	2.8541501
Pd	0.0000000	0.0000000	1.8525048
I	0.0000000	0.0000000	-1.5235524
C	0.0000000	0.0000000	-3.6036089
C	0.0000000	1.1907450	-4.3154589
C	0.0000000	-1.1907450	-4.3154589
C	-0.0000000	1.1988073	-5.7009552
F	-0.0000000	2.3571143	-3.6867151
C	-0.0000000	-1.1988073	-5.7009552
F	-0.0000000	-2.3571143	-3.6867151
C	0.0000000	0.0000000	-6.3947177
F	0.0000000	2.3436457	-6.3674508

F	0.0000000	-2.3436457	-6.3674508
F	0.0000000	0.0000000	-7.7169204

13B:C_6F5$_I$

C	3.1216763	-1.2232161	-1.9243021
H	3.7544394	-2.1037175	-1.9118124
C	3.7650320	0.0000000	-1.7189861
C	3.1216763	1.2232161	-1.9243021
H	3.7544394	2.1037175	-1.9118124
C	-3.1216763	1.2232161	-1.9243021
H	-3.7544394	2.1037175	-1.9118124
C	-3.7650320	0.0000000	-1.7189861
C	-3.1216763	-1.2232161	-1.9243021
H	-3.7544394	-2.1037175	-1.9118124
N	-5.1190412	0.0000000	-1.3445046
N	5.1190412	0.0000000	-1.3445046
C	-5.8720475	1.2210742	-1.4787534
H	-5.9148027	1.6001074	-2.5123766
H	-6.8926822	1.0415353	-1.1435593
H	-5.4637656	2.0104863	-0.8413017
C	-5.8720475	-1.2210742	-1.4787534
H	-5.4637656	-2.0104863	-0.8413017
H	-6.8926822	-1.0415353	-1.1435593
H	-5.9148027	-1.6001074	-2.5123766
C	5.8720475	-1.2210742	-1.4787534
H	5.9148027	-1.6001074	-2.5123766
H	6.8926822	-1.0415353	-1.1435593
H	5.4637656	-2.0104863	-0.8413017
C	5.8720475	1.2210742	-1.4787534
H	5.4637656	2.0104863	-0.8413017
H	6.8926822	1.0415353	-1.1435593
H	5.9148027	1.6001074	-2.5123766
S	-1.5464910	-1.6793465	-2.2074273
S	-1.5464910	1.6793465	-2.2074273
S	1.5464910	-1.6793465	-2.2074273

S	1.5464910	1.6793465	-2.2074273
Pd	0.0000000	0.0000000	-2.1856608
I	0.0000000	0.0000000	1.2717260
C	0.0000000	0.0000000	3.3535653
C	0.0000000	-1.1902183	4.0664147
C	0.0000000	1.1902183	4.0664147
C	-0.0000000	-1.1986933	5.4519272
F	-0.0000000	-2.3574181	3.4383442
C	-0.0000000	1.1986933	5.4519272
F	0.0000000	2.3574181	3.4383442
C	0.0000000	0.0000000	6.1458413
F	0.0000000	-2.3438364	6.1188792
F	0.0000000	2.3438364	6.1188792
F	0.0000000	0.0000000	7.4685103

14A:C_6F5_I

C	2.5982749	-1.2112020	1.6102028
H	3.1918625	-2.1288385	1.5929959
C	3.2874739	0.0000000	1.5159874
C	2.5982749	1.2112020	1.6102028
H	3.1918625	2.1288385	1.5929959
O	1.3606077	-1.4306970	1.7221995
O	1.3606077	1.4306970	1.7221995
C	-2.5982749	1.2112020	1.6102028
H	-3.1918625	2.1288385	1.5929959
C	-3.2874739	0.0000000	1.5159874
C	-2.5982749	-1.2112020	1.6102028
H	-3.1918625	-2.1288385	1.5929959
O	-1.3606077	1.4306970	1.7221995
O	-1.3606077	-1.4306970	1.7221995
C	-4.8186075	-0.0000000	1.4153138
C	4.8186075	0.0000000	1.4153138
C	-5.3269972	-1.2309286	0.6576391
H	-5.1596248	-2.1614210	1.2037541
H	-6.4055829	-1.1461018	0.5058855
H	-4.8519350	-1.3177083	-0.3227486

C	-5.3269972	1.2309286	0.6576391
H	-6.4055829	1.1461018	0.5058855
H	-5.1596248	2.1614210	1.2037541
H	-4.8519350	1.3177083	-0.3227486
C	-5.4223241	0.0000000	2.8236376
H	-6.5153309	0.0000000	2.7811085
H	-5.1034911	-0.8826080	3.3841088
H	-5.1034911	0.8826080	3.3841088
C	5.4223241	0.0000000	2.8236376
H	5.1034911	-0.8826080	3.3841088
H	6.5153309	0.0000000	2.7811085
H	5.1034911	0.8826080	3.3841088
C	5.3269972	-1.2309286	0.6576391
H	6.4055829	-1.1461018	0.5058855
H	5.1596248	-2.1614210	1.2037541
H	4.8519350	-1.3177083	-0.3227486
C	5.3269972	1.2309286	0.6576391
H	4.8519350	1.3177083	-0.3227486
H	5.1596248	2.1614210	1.2037541
H	6.4055829	1.1461018	0.5058855
Pd	0.0000000	0.0000000	1.7311828
I	0.0000000	0.0000000	-1.5929447
C	0.0000000	0.0000000	-3.6740121
C	0.0000000	1.1908827	-4.3855713
C	0.0000000	-1.1908827	-4.3855713
C	0.0000000	1.1990286	-5.7710586
F	0.0000000	2.3571051	-3.7563186
C	0.0000000	-1.1990286	-5.7710586
F	0.0000000	-2.3571051	-3.7563186
C	0.0000000	0.0000000	-6.4646787
F	-0.0000000	2.3437466	-6.4376280
F	-0.0000000	-2.3437466	-6.4376280
F	0.0000000	0.0000000	-7.7866368

14B:C_6F5$_I$

C	3.1177866	-1.2211162	-1.6912357
H	3.7557010	-2.0995733	-1.6575365
C	3.7664294	0.0000000	-1.5551453
C	3.1177866	1.2211162	-1.6912357
H	3.7557010	2.0995733	-1.6575365
C	-3.1177866	1.2211162	-1.6912357
H	-3.7557010	2.0995733	-1.6575365
C	-3.7664294	0.0000000	-1.5551453
C	-3.1177866	-1.2211162	-1.6912357
H	-3.7557010	-2.0995733	-1.6575365
C	-5.2961575	0.0000000	-1.3608207
C	5.2961575	0.0000000	-1.3608207
C	-5.7617893	1.2311071	-0.5772287
H	-5.6419093	2.1602941	-1.1377355
H	-6.8268632	1.1380374	-0.3538887
H	-5.2239464	1.3281649	0.3690416
C	-5.7617893	-1.2311071	-0.5772287
H	-6.8268632	-1.1380374	-0.3538887
H	-5.6419093	-2.1602941	-1.1377355
H	-5.2239464	-1.3281649	0.3690416
C	-5.9662240	0.0000000	-2.7383782
H	-7.0552000	0.0000000	-2.6382121
H	-5.6766118	0.8829346	-3.3137933
H	-5.6766118	-0.8829346	-3.3137933
C	5.9662240	0.0000000	-2.7383782
H	5.6766118	0.8829346	-3.3137933
H	7.0552000	0.0000000	-2.6382121
H	5.6766118	-0.8829346	-3.3137933
C	5.7617893	1.2311071	-0.5772287
H	6.8268632	1.1380374	-0.3538887
H	5.6419093	2.1602941	-1.1377355
H	5.2239464	1.3281649	0.3690416
C	5.7617893	-1.2311071	-0.5772287
H	5.2239464	-1.3281649	0.3690416
H	5.6419093	-2.1602941	-1.1377355

H	6.8268632	-1.1380374	-0.3538887
S	-1.5391485	1.6926145	-1.9130475
S	-1.5391485	-1.6926145	-1.9130475
S	1.5391485	-1.6926145	-1.9130475
S	1.5391485	1.6926145	-1.9130475
Pd	0.0000000	0.0000000	-1.9187611
I	0.0000000	0.0000000	1.5422222
C	0.0000000	0.0000000	3.6223682
C	-0.0000000	-1.1906538	4.3343672
C	-0.0000000	1.1906538	4.3343672
C	0.0000000	-1.1990035	5.7198213
F	-0.0000000	-2.3571044	3.7052656
C	0.0000000	1.1990035	5.7198213
F	-0.0000000	2.3571044	3.7052656
C	0.0000000	0.0000000	6.4135131
F	0.0000000	-2.3439505	6.3863794
F	0.0000000	2.3439505	6.3863794
F	0.0000000	0.0000000	7.7358003

15A:C_6F5_I

C	-2.5783735	1.2319438	2.7740111
H	-3.2000842	2.1318221	2.7599454
C	-3.2218161	-0.0000000	2.7438199
H	-4.3023607	0.0000000	2.7027076
C	-2.5783735	-1.2319438	2.7740111
H	-3.2000842	-2.1318221	2.7599454
O	-1.3377714	1.4668291	2.8194615
O	-1.3377714	-1.4668291	2.8194615
C	2.5783735	-1.2319438	2.7740111
H	3.2000842	-2.1318221	2.7599454
C	3.2218161	0.0000000	2.7438199
H	4.3023607	0.0000000	2.7027076
C	2.5783735	1.2319438	2.7740111
H	3.2000842	2.1318221	2.7599454
O	1.3377714	-1.4668291	2.8194615

O	1.3377714	1.4668291	2.8194615
Pt	0.0000000	0.0000000	2.8213419
I	0.0000000	0.0000000	-0.4969235
C	0.0000000	0.0000000	-2.5833132
C	0.0000000	-1.1903034	-3.2950791
C	0.0000000	1.1903034	-3.2950791
C	-0.0000000	-1.1991257	-4.6805441
F	-0.0000000	-2.3570367	-2.6657173
C	-0.0000000	1.1991257	-4.6805441
F	-0.0000000	2.3570367	-2.6657173
C	0.0000000	0.0000000	-5.3740636
F	0.0000000	-2.3439308	-5.3473535
F	0.0000000	2.3439308	-5.3473535
F	0.0000000	0.0000000	-6.6963808

15B:C_6F5_I

C	-3.0938258	1.2448219	2.8411328
H	-3.7681642	2.0985713	2.8203415
C	-3.6976846	-0.0000000	2.8062116
H	-4.7813112	0.0000000	2.7548974
C	-3.0938258	-1.2448219	2.8411328
H	-3.7681642	-2.0985713	2.8203415
C	3.0938258	-1.2448219	2.8411328
H	3.7681642	-2.0985713	2.8203415
C	3.6976846	0.0000000	2.8062116
H	4.7813112	0.0000000	2.7548974
C	3.0938258	1.2448219	2.8411328
H	3.7681642	2.0985713	2.8203415
Pt	0.0000000	0.0000000	2.9199078
S	-1.5081416	-1.7305136	2.9125663
S	-1.5081416	1.7305136	2.9125663
S	1.5081416	-1.7305136	2.9125663
S	1.5081416	1.7305136	2.9125663
I	0.0000000	0.0000000	-0.6034129
C	0.0000000	0.0000000	-2.6836134
C	-0.0000000	-1.1906752	-3.3954882

C	-0.0000000	1.1906752	-3.3954882
C	0.0000000	-1.1990550	-4.7809439
F	0.0000000	-2.3570317	-2.7661399
C	0.0000000	1.1990550	-4.7809439
F	0.0000000	2.3570317	-2.7661399
C	0.0000000	0.0000000	-5.4746294
F	0.0000000	-2.3440049	-5.4473409
F	0.0000000	2.3440049	-5.4473409
F	0.0000000	0.0000000	-6.7968069

16A:C_6F5_I

C	2.5780191	-1.2394351	2.7930575
H	3.2269467	-2.1189656	2.7781754
C	3.1994118	0.0000000	2.7656919
C	2.5780191	1.2394351	2.7930575
H	3.2269467	2.1189656	2.7781754
O	1.3354002	-1.4683257	2.8359000
O	1.3354002	1.4683257	2.8359000
C	-2.5780191	1.2394351	2.7930575
H	-3.2269467	2.1189656	2.7781754
C	-3.1994118	0.0000000	2.7656919
C	-2.5780191	-1.2394351	2.7930575
H	-3.2269467	-2.1189656	2.7781754
O	-1.3354002	1.4683257	2.8359000
O	-1.3354002	-1.4683257	2.8359000
F	4.5493354	0.0000000	2.7182382
F	-4.5493354	0.0000000	2.7182382
Pt	0.0000000	0.0000000	2.8386014
I	0.0000000	0.0000000	-0.5269061
C	0.0000000	0.0000000	-2.6098637
C	0.0000000	1.1910130	-3.3205685
C	0.0000000	-1.1910130	-3.3205685
C	-0.0000000	1.1993484	-4.7059928
F	-0.0000000	2.3569317	-2.6905887
C	-0.0000000	-1.1993484	-4.7059928

F	0.0000000	-2.3569317	-2.6905887
C	0.0000000	0.0000000	-5.3994800
F	0.0000000	2.3441512	-5.3716753
F	0.0000000	-2.3441512	-5.3716753
F	-0.0000000	0.0000000	-6.7210930

16B:C_6F5_I

C	3.0877007	-1.2496097	2.8508301
H	3.7921793	-2.0780756	2.8297604
C	3.6669582	-0.0000000	2.8166884
C	3.0877007	1.2496097	2.8508301
H	3.7921793	2.0780756	2.8297604
C	-3.0877007	1.2496097	2.8508301
H	-3.7921793	2.0780756	2.8297604
C	-3.6669582	0.0000000	2.8166884
C	-3.0877007	-1.2496097	2.8508301
H	-3.7921793	-2.0780756	2.8297604
F	5.0184917	-0.0000000	2.7514432
F	-5.0184917	0.0000000	2.7514432
Pt	0.0000000	0.0000000	2.9283820
S	1.5021925	1.7337821	2.9209304
S	1.5021925	-1.7337821	2.9209304
S	-1.5021925	1.7337821	2.9209304
S	-1.5021925	-1.7337821	2.9209304
I	0.0000000	0.0000000	-0.6169340
C	0.0000000	0.0000000	-2.6956892
C	0.0000000	1.1912006	-3.4065392
C	0.0000000	-1.1912006	-3.4065392
C	0.0000000	1.1993236	-4.7919541
F	-0.0000000	2.3567719	-2.7762686
C	0.0000000	-1.1993236	-4.7919541
F	0.0000000	-2.3567719	-2.7762686
C	0.0000000	0.0000000	-5.4853809
F	-0.0000000	2.3438446	-5.4580883
F	0.0000000	-2.3438446	-5.4580883

F	-0.0000000	0.0000000	-6.8070245

17A:C_6F5_I

C	-2.5803457	1.2428024	2.6434172
H	-3.2050689	2.1396127	2.6266679
C	-3.2340544	-0.0000000	2.6094708
C	-2.5803457	-1.2428024	2.6434172
H	-3.2050689	-2.1396127	2.6266679
O	-1.3446615	1.4574507	2.6943829
O	-1.3446615	-1.4574507	2.6943829
C	2.5803457	-1.2428024	2.6434172
H	3.2050689	-2.1396127	2.6266679
C	3.2340544	0.0000000	2.6094708
C	2.5803457	1.2428024	2.6434172
H	3.2050689	2.1396127	2.6266679
O	1.3446615	-1.4574507	2.6943829
O	1.3446615	1.4574507	2.6943829
Pt	0.0000000	0.0000000	2.7000150
C	4.6556852	0.0000000	2.5497750
C	-4.6556852	0.0000000	2.5497750
N	5.8068954	0.0000000	2.5024809
N	-5.8068954	-0.0000000	2.5024809
I	0.0000000	0.0000000	-0.7373144
C	0.0000000	0.0000000	-2.8159541
C	-0.0000000	-1.1924707	-3.5242692
C	-0.0000000	1.1924707	-3.5242692
C	0.0000000	-1.2000767	-4.9096793
F	-0.0000000	-2.3564187	-2.8918788
C	0.0000000	1.2000767	-4.9096793
F	0.0000000	2.3564187	-2.8918788
C	-0.0000000	0.0000000	-5.6023849
F	0.0000000	-2.3435865	-5.5755801
F	0.0000000	2.3435865	-5.5755801
F	-0.0000000	0.0000000	-6.9228723

17B:C_6F5$_I$

C	3.0888922	-1.2528379	2.6850864
H	3.7661444	-2.1039768	2.6648971
C	3.7033889	0.0000000	2.6504161
C	3.0888922	1.2528379	2.6850864
H	3.7661444	2.1039768	2.6648971
C	-3.0888922	1.2528379	2.6850864
H	-3.7661444	2.1039768	2.6648971
C	-3.7033889	0.0000000	2.6504161
C	-3.0888922	-1.2528379	2.6850864
H	-3.7661444	-2.1039768	2.6648971
Pt	0.0000000	0.0000000	2.7636807
S	1.5085799	1.7256742	2.7540626
S	1.5085799	-1.7256742	2.7540626
S	-1.5085799	-1.7256742	2.7540626
S	-1.5085799	1.7256742	2.7540626
C	-5.1319088	0.0000000	2.5836649
C	5.1319088	-0.0000000	2.5836649
N	-6.2827637	0.0000000	2.5302745
N	6.2827637	0.0000000	2.5302745
I	0.0000000	0.0000000	-0.8074474
C	0.0000000	0.0000000	-2.8840578
C	0.0000000	-1.1921829	-3.5930835
C	0.0000000	1.1921829	-3.5930835
C	0.0000000	-1.1998539	-4.9784583
F	0.0000000	-2.3564642	-2.9611454
C	0.0000000	1.1998539	-4.9784583
F	0.0000000	2.3564642	-2.9611454
C	0.0000000	0.0000000	-5.6714515
F	0.0000000	-2.3437287	-5.6440531
F	0.0000000	2.3437287	-5.6440531
F	0.0000000	0.0000000	-6.9921389

18A:C_6F5$_I$

C	2.5836451	-1.2461928	2.4918946

H	3.2193446	-2.1320284	2.4767665
C	3.2196634	0.0000000	2.4624528
C	2.5836451	1.2461928	2.4918946
H	3.2193446	2.1320284	2.4767665
O	1.3465462	-1.4552257	2.5366782
O	1.3465462	1.4552257	2.5366782
C	-2.5836451	1.2461928	2.4918946
H	-3.2193446	2.1320284	2.4767665
C	-3.2196634	0.0000000	2.4624528
C	-2.5836451	-1.2461928	2.4918946
H	-3.2193446	-2.1320284	2.4767665
O	-1.3465462	1.4552257	2.5366782
O	-1.3465462	-1.4552257	2.5366782
N	-4.6705929	0.0000000	2.4076280
N	4.6705929	-0.0000000	2.4076280
O	-5.2341881	-1.0746837	2.3862086
O	-5.2341881	1.0746837	2.3862086
O	5.2341881	-1.0746837	2.3862086
O	5.2341881	1.0746837	2.3862086
Pt	0.0000000	0.0000000	2.5414128
I	0.0000000	0.0000000	-0.9016221
C	0.0000000	0.0000000	-2.9799440
C	-0.0000000	1.1925081	-3.6881441
C	-0.0000000	-1.1925081	-3.6881441
C	0.0000000	1.2000580	-5.0735299
F	0.0000000	2.3563660	-3.0558860
C	0.0000000	-1.2000580	-5.0735299
F	-0.0000000	-2.3563660	-3.0558860
C	0.0000000	0.0000000	-5.7662987
F	0.0000000	2.3436827	-5.7390428
F	0.0000000	-2.3436827	-5.7390428
F	0.0000000	0.0000000	-7.0866954

18B:C_6F5_I

C	3.0815352	-1.2627498	2.5237612

H	3.7886652	-2.0999343	2.5094837
C	3.7005791	0.0000000	2.4887986
C	3.0815352	1.2627498	2.5237612
H	3.7886652	2.0999343	2.5094837
C	-3.0815352	1.2627498	2.5237612
H	-3.7886652	2.0999343	2.5094837
C	-3.7005791	0.0000000	2.4887986
C	-3.0815352	-1.2627498	2.5237612
H	-3.7886652	-2.0999343	2.5094837
N	-5.0435107	-0.0000000	2.4277451
N	5.0435107	0.0000000	2.4277451
O	-5.7762654	-1.0531601	2.3970515
O	-5.7762654	1.0531601	2.3970515
O	5.7762654	-1.0531601	2.3970515
O	5.7762654	1.0531601	2.3970515
Pt	0.0000000	0.0000000	2.5833960
S	1.5110736	1.7053213	2.5829449
S	1.5110736	-1.7053213	2.5829449
S	-1.5110736	-1.7053213	2.5829449
S	-1.5110736	1.7053213	2.5829449
I	0.0000000	0.0000000	-0.9491427
C	0.0000000	0.0000000	-3.0328357
C	0.0000000	1.1922397	-3.7405445
C	0.0000000	-1.1922397	-3.7405445
C	0.0000000	1.2002551	-5.1259233
F	0.0000000	2.3559125	-3.1065728
C	0.0000000	-1.2002551	-5.1259233
F	0.0000000	-2.3559125	-3.1065728
C	-0.0000000	0.0000000	-5.8185009
F	0.0000000	2.3436559	-5.7919584
F	0.0000000	-2.3436559	-5.7919584
F	0.0000000	0.0000000	-7.1389711

19A:C_6F5_I

C	2.5905767	-1.2214670	2.4777133

H	3.2075268	-2.1267495	2.4680902
C	3.2569591	0.0000000	2.4180941
C	2.5905767	1.2214670	2.4777133
H	3.2075268	2.1267495	2.4680902
O	1.3459387	-1.4514419	2.5485930
O	1.3459387	1.4514419	2.5485930
C	-2.5905767	1.2214670	2.4777133
H	-3.2075268	2.1267495	2.4680902
C	-3.2569591	0.0000000	2.4180941
C	-2.5905767	-1.2214670	2.4777133
H	-3.2075268	-2.1267495	2.4680902
O	-1.3459387	1.4514419	2.5485930
O	-1.3459387	-1.4514419	2.5485930
N	-4.6826245	0.0000000	2.4071994
N	4.6826245	0.0000000	2.4071994
H	-5.0496209	0.8134989	1.9328561
H	-5.0496209	-0.8134989	1.9328561
H	5.0496209	0.8134989	1.9328561
H	5.0496209	-0.8134989	1.9328561
Pt	0.0000000	0.0000000	2.5492180
I	0.0000000	0.0000000	-0.7246656
C	0.0000000	0.0000000	-2.8147921
C	0.0000000	1.1898220	-3.5270971
C	0.0000000	-1.1898220	-3.5270971
C	-0.0000000	1.1990379	-4.9126043
F	-0.0000000	2.3571325	-2.8979534
C	-0.0000000	-1.1990379	-4.9126043
F	-0.0000000	-2.3571325	-2.8979534
C	0.0000000	0.0000000	-5.6062838
F	-0.0000000	2.3440409	-5.5795381
F	-0.0000000	-2.3440409	-5.5795381
F	-0.0000000	0.0000000	-6.9286878

19B:C_6F5_I

C	3.1023297	-1.2331182	2.5434612

H	3.7653264	-2.0978203	2.5326910
C	3.7379620	0.0000000	2.4740904
C	3.1023297	1.2331182	2.5434612
H	3.7653264	2.0978203	2.5326910
C	-3.1023297	1.2331182	2.5434612
H	-3.7653264	2.0978203	2.5326910
C	-3.7379620	-0.0000000	2.4740904
C	-3.1023297	-1.2331182	2.5434612
H	-3.7653264	-2.0978203	2.5326910
N	-5.1498385	0.0000000	2.4238958
N	5.1498385	0.0000000	2.4238958
H	-5.5313219	0.8231331	1.9825872
H	-5.5313219	-0.8231331	1.9825872
H	5.5313219	0.8231331	1.9825872
H	5.5313219	-0.8231331	1.9825872
Pt	0.0000000	0.0000000	2.6542914
S	1.5155306	1.7143407	2.6554567
S	1.5155306	-1.7143407	2.6554567
S	-1.5155306	1.7143407	2.6554567
S	-1.5155306	-1.7143407	2.6554567
I	0.0000000	0.0000000	-0.8479367
C	0.0000000	0.0000000	-2.9304510
C	-0.0000000	1.1901897	-3.6430307
C	-0.0000000	-1.1901897	-3.6430307
C	0.0000000	1.1988996	-5.0284992
F	0.0000000	2.3571089	-3.0141774
C	0.0000000	-1.1988996	-5.0284992
F	0.0000000	-2.3571089	-3.0141774
C	0.0000000	0.0000000	-5.7223452
F	0.0000000	2.3440308	-5.6950713
F	0.0000000	-2.3440308	-5.6950713
F	0.0000000	0.0000000	-7.0447579

20A:C_6F5_I

C	2.6063395	-1.2181581	1.7237342

H	3.1903692	-2.1376551	1.7226392
C	3.2931840	0.0000000	1.6351605
C	2.6063395	1.2181581	1.7237342
H	3.1903692	2.1376551	1.7226392
O	1.3585708	-1.4318121	1.8102026
O	1.3585708	1.4318121	1.8102026
C	-2.6063395	1.2181581	1.7237342
H	-3.1903692	2.1376551	1.7226392
C	-3.2931840	-0.0000000	1.6351605
C	-2.6063395	-1.2181581	1.7237342
H	-3.1903692	-2.1376551	1.7226392
O	-1.3585708	1.4318121	1.8102026
O	-1.3585708	-1.4318121	1.8102026
N	-4.7026694	0.0000000	1.4499067
N	4.7026694	-0.0000000	1.4499067
C	-5.3969687	-1.2095031	1.8185042
H	-5.2136810	-1.5232516	2.8603236
H	-6.4675140	-1.0492616	1.6951084
H	-5.1254275	-2.0385533	1.1600482
C	-5.3969687	1.2095031	1.8185042
H	-5.1254275	2.0385533	1.1600482
H	-6.4675140	1.0492616	1.6951084
H	-5.2136810	1.5232516	2.8603236
C	5.3969687	1.2095031	1.8185042
H	5.2136810	1.5232516	2.8603236
H	6.4675140	1.0492616	1.6951084
H	5.1254275	2.0385533	1.1600482
C	5.3969687	-1.2095031	1.8185042
H	5.1254275	-2.0385533	1.1600482
H	6.4675140	-1.0492616	1.6951084
H	5.2136810	-1.5232516	2.8603236
Pt	0.0000000	0.0000000	1.8025656
I	-0.0000000	0.0000000	-1.4929152
C	-0.0000000	0.0000000	-3.5828168
C	0.0000000	1.1896376	-4.2957812

C	0.0000000	-1.1896376	-4.2957812
C	-0.0000000	1.1988060	-5.6813380
F	-0.0000000	2.3573758	-3.6674856
C	-0.0000000	-1.1988060	-5.6813380
F	-0.0000000	-2.3573758	-3.6674856
C	-0.0000000	0.0000000	-6.3751818
F	0.0000000	2.3440847	-6.3484235
F	0.0000000	-2.3440847	-6.3484235
F	0.0000000	0.0000000	-7.6979710

20B:C_6F5$_I$

C	3.1217154	-1.2270377	1.7821414
H	3.7479003	-2.1115949	1.7884598
C	3.7864389	0.0000000	1.7269888
C	3.1217154	1.2270377	1.7821414
H	3.7479003	2.1115949	1.7884598
C	-3.1217154	1.2270377	1.7821414
H	-3.7479003	2.1115949	1.7884598
C	-3.7864389	0.0000000	1.7269888
C	-3.1217154	-1.2270377	1.7821414
H	-3.7479003	-2.1115949	1.7884598
N	-5.1891076	0.0000000	1.6126117
N	5.1891076	0.0000000	1.6126117
C	-5.8972057	-1.2205493	1.9088727
H	-5.6983585	-1.6072633	2.9209204
H	-6.9663237	-1.0342834	1.8233906
H	-5.6576353	-2.0079882	1.1887076
C	-5.8972057	1.2205493	1.9088727
H	-5.6576353	2.0079882	1.1887076
H	-6.9663237	1.0342834	1.8233906
H	-5.6983585	1.6072633	2.9209204
C	5.8972057	1.2205493	1.9088727
H	5.6983585	1.6072633	2.9209204
H	6.9663237	1.0342834	1.8233906
H	5.6576353	2.0079882	1.1887076

C	5.8972057	-1.2205493	1.9088727
H	5.6576353	-2.0079882	1.1887076
H	6.9663237	-1.0342834	1.8233906
H	5.6983585	-1.6072633	2.9209204
Pt	0.0000000	0.0000000	1.8235096
S	-1.5268778	-1.6961716	1.8375093
S	-1.5268778	1.6961716	1.8375093
S	1.5268778	-1.6961716	1.8375093
S	1.5268778	1.6961716	1.8375093
I	0.0000000	0.0000000	-1.6963809
C	0.0000000	0.0000000	-3.7793039
C	0.0000000	-1.1899492	-4.4925404
C	-0.0000000	1.1899492	-4.4925404
C	0.0000000	-1.1986464	-5.8780075
F	-0.0000000	-2.3573379	-3.8645211
C	0.0000000	1.1986464	-5.8780075
F	0.0000000	2.3573379	-3.8645211
C	0.0000000	0.0000000	-6.5720541
F	0.0000000	-2.3439112	-6.5449657
F	0.0000000	2.3439112	-6.5449657
F	0.0000000	0.0000000	-7.8949091

21A:C_6F5_I

C	2.5971017	-1.2145166	-1.5955126
H	3.1888274	-2.1311878	-1.5831904
C	3.2793009	-0.0000000	-1.5076418
C	2.5971017	1.2145166	-1.5955126
H	3.1888274	2.1311878	-1.5831904
O	1.3553531	-1.4405186	-1.6969761
O	1.3553531	1.4405186	-1.6969761
C	-2.5971017	1.2145166	-1.5955126
H	-3.1888274	2.1311878	-1.5831904
C	-3.2793009	-0.0000000	-1.5076418
C	-2.5971017	-1.2145166	-1.5955126
H	-3.1888274	-2.1311878	-1.5831904

O	-1.3553531	1.4405186	-1.6969761
O	-1.3553531	-1.4405186	-1.6969761
C	-4.8120276	-0.0000000	-1.4137091
C	4.8120276	0.0000000	-1.4137091
C	-5.3234228	1.2308701	-0.6577546
H	-5.1568429	2.1614052	-1.2038839
H	-6.4021153	1.1443651	-0.5085169
H	-4.8508342	1.3187321	0.3237422
C	-5.3234228	-1.2308701	-0.6577546
H	-6.4021153	-1.1443651	-0.5085169
H	-5.1568429	-2.1614052	-1.2038839
H	-4.8508342	-1.3187321	0.3237422
C	-5.4089427	-0.0000000	-2.8248062
H	-6.5021847	0.0000000	-2.7865514
H	-5.0881622	0.8827117	-3.3840412
H	-5.0881622	-0.8827117	-3.3840412
C	5.4089427	0.0000000	-2.8248062
H	5.0881622	0.8827117	-3.3840412
H	6.5021847	0.0000000	-2.7865514
H	5.0881622	-0.8827117	-3.3840412
C	5.3234228	1.2308701	-0.6577546
H	6.4021153	1.1443651	-0.5085169
H	5.1568429	2.1614052	-1.2038839
H	4.8508342	1.3187321	0.3237422
C	5.3234228	-1.2308701	-0.6577546
H	4.8508342	-1.3187321	0.3237422
H	5.1568429	-2.1614052	-1.2038839
H	6.4021153	-1.1443651	-0.5085169
Pt	0.0000000	0.0000000	-1.6973561
I	0.0000000	0.0000000	1.5638839
C	0.0000000	0.0000000	3.6545635
C	-0.0000000	-1.1897050	4.3669807
C	-0.0000000	1.1897050	4.3669807
C	-0.0000000	-1.1989786	5.7524835
F	0.0000000	-2.3571670	3.7380360

C	-0.0000000	1.1989786	5.7524835
F	0.0000000	2.3571670	3.7380360
C	0.0000000	0.0000000	6.4462162
F	0.0000000	-2.3441310	6.4194209
F	0.0000000	2.3441310	6.4194209
F	-0.0000000	0.0000000	7.7688007

21B:C_6F5_I

C	-3.1149246	1.2236644	-1.5118504
H	-3.7500019	2.1039988	-1.5197069
C	-3.7727885	0.0000000	-1.5062830
C	-3.1149246	-1.2236644	-1.5118504
H	-3.7500019	-2.1039988	-1.5197069
C	3.1149246	-1.2236644	-1.5118504
H	3.7500019	-2.1039988	-1.5197069
C	3.7727885	0.0000000	-1.5062830
C	3.1149246	1.2236644	-1.5118504
H	3.7500019	2.1039988	-1.5197069
C	5.3129236	0.0000000	-1.5932318
C	-5.3129236	0.0000000	-1.5932318
C	5.9210112	-1.2305730	-0.9140445
H	5.6927428	-2.1604832	-1.4385431
H	7.0091559	-1.1383841	-0.9053994
H	5.5801721	-1.3252190	0.1198529
C	5.9210112	1.2305730	-0.9140445
H	7.0091559	1.1383841	-0.9053994
H	5.6927428	2.1604832	-1.4385431
H	5.5801721	1.3252190	0.1198529
C	5.7081252	-0.0000000	-3.0739830
H	6.7964154	0.0000000	-3.1848776
H	5.3132626	-0.8827827	-3.5828611
H	5.3132626	0.8827827	-3.5828611
C	-5.7081252	0.0000000	-3.0739830
H	-5.3132626	-0.8827827	-3.5828611
H	-6.7964154	0.0000000	-3.1848776

H	-5.3132626	0.8827827	-3.5828611
C	-5.9210112	-1.2305730	-0.9140445
H	-7.0091559	-1.1383841	-0.9053994
H	-5.6927428	-2.1604832	-1.4385431
H	-5.5801721	-1.3252190	0.1198529
C	-5.9210112	1.2305730	-0.9140445
H	-5.5801721	1.3252190	0.1198529
H	-5.6927428	2.1604832	-1.4385431
H	-7.0091559	1.1383841	-0.9053994
Pt	0.0000000	0.0000000	-1.5085409
S	1.5240963	-1.7045744	-1.5128426
S	1.5240963	1.7045744	-1.5128426
S	-1.5240963	1.7045744	-1.5128426
S	-1.5240963	-1.7045744	-1.5128426
I	0.0000000	0.0000000	2.0136056
C	0.0000000	0.0000000	4.0952176
C	0.0000000	1.1903718	4.8078379
C	0.0000000	-1.1903718	4.8078379
C	0.0000000	1.1988355	6.1933695
F	-0.0000000	2.3572681	4.1791702
C	0.0000000	-1.1988355	6.1933695
F	-0.0000000	-2.3572681	4.1791702
C	-0.0000000	0.0000000	6.8872487
F	-0.0000000	2.3438509	6.8601450
F	-0.0000000	-2.3438509	6.8601450
F	0.0000000	0.0000000	8.2097554
